# Supplementary material for: LeMaRns: A Length-based Multi-species analysis by numerical simulation in R
Source: PLoS One. 2020 Feb 3;15(2):e0227767. doi: 10.1371/journal.pone.0227767 (PMC6996808; doi:10.1371/journal.pone.0227767)
Supplement: S2 File — Contains the description of the model and further explanation of the package. (PDF) [file pone.0227767.s002.pdf]

LeMaRns

Length-based Multispecies Analysis by Numerical Simulation in R

Michael A. Spence, Hayley J. Bannister, Johnathan E. Ball and Robert B. Thorpe

2019-10-09

Introduction

LeMans (Length-based Multispecies analysis by numerical simulation) is a length-structured fish community model, with the fish community being represented in terms of both species and length (Hall et al. 2006). By representing many processes, including fishing, natural mortality, and predation as functions of length, it is possible to reproduce many aspects of the community dynamics (including the tendency of diet to change with increasing predator size) with a relatively small number of parameters, and modest requirement for data in model set up. This makes the framework particularly suitable for use in data-limited fisheries.

Most assessment models have a single-species focus, which is appropriate for assessing the current state of the stock and making a very short term forecast, but in the longer-term, stock mortality and abundance will depend on its interactions with other parts of the food-web, and so in general any longer-term projections require the use of a multispecies model.

Assessment models are typically age-structured, i.e. they split the stock(s) being modelled into age classes (e.g. 1 year or 2 year old cod). This is the natural time frame for assimilating annual recruitment into a single-stock, but it is not useful for multispecies interactions, which are typically based upon the relative sizes of predator and prey, rather than being age-dependent. Consequently, LeMans represents stocks in terms of their length. This allows predation to emerge as a function of the relative lengths of predator and prey, and enables the observed shifts in diet as fish grow larger to emerge naturally rather than having to be ignored or hardwired (as for example happens in bulk biomass models such as surplus-production models or indeed Ecopath (Polovina 1984)). Size-structuring can be done either by weight (e.g. mizer; Scott, Blanchard, and Andersen (2014)), or length (e.g. FishSUMS; Speirs et al. (2010)). In LeMans we choose length because a) it is generally easier to measure than weight in the field (Conner, Matson, and Kelly 2017), and b) fisheries selectivity is normally characterised in terms of a relationship with length, which can more easily be related to the structure of fishing nets, and hence it is easier to relate to the parameterisation of mixed fisheries.

Unlike many size-based models, LeMans models predation in terms of a simple type II functional response rather than through the determination of encounter probabilities, and growth is assumed to follow the von Bertalanffy relationship rather than being food-limited, so the model would not be suitable for use in strongly food-limited ecosystems (e.g. the Baltic) unless modified. Where possible, generic relationships with length are applied to the key model processes, and this together with the assumption of von Bertalanffy growth makes the model data requirements relatively modest, facilitating its use in data-limited situations. Another key strength of the model is its ability to accommodate fleet dynamics (due to its length-based structure, simplicity, and speed of execution).

No model structure is perfect, and all involve making some trade-offs between comprehensiveness, run-time, flexibility and transparency, but LeMans is well suited for use whenever there is a need for multispecies or mixed fisheries analysis, but insufficient data to support the use of more complicated model, such as Ecopath or Atlantis (Fulton et al. 2011), provided that growth is not strongly food-limited.

Setting up the model

Dataset for the North Sea

In the `LeMaRns` package we supply data for the North Sea model (Thorpe et al. 2015). This includes the species parameters `NS_par` and the interaction matrix `NS_tau`.

```
NS_par
#>      species_names   Linf    W_a    W_b     k  Lmat           a
#> 1            Sprat  16.55 0.0059 3.1088 0.681 12.14 885.6667731
#> 2            Npout  23.75 0.0075 3.0244 0.849 17.21 225.0988055
#> 3          Sandeel  23.57 0.0049 2.7826 1.000 12.16 231.6879245
#> 4         Poor cod  23.00 0.0082 3.0865 0.520 15.00 254.2279144
#> 5  Long rough dab  25.00 0.0053 3.1434 0.340 15.00 185.3071561
#> 6              Dab  32.40 0.0159 2.8639 0.536 12.96  69.3184724
#> 7          Herring  33.35 0.0048 3.1984 0.606 23.39  62.1228287
#> 8  Horse mackerel  28.00 0.0316 2.6520 0.380 19.00 120.5695838
#> 9       Lemon sole  37.00 0.0123 2.9713 0.420 27.00  41.8978427
#> 10            Sole  46.41 0.0089 3.0172 0.284 20.96  17.7411663
#> 11        Mackerel  38.00 0.0052 3.1674 0.510 26.00  37.8676686
#> 12         Whiting  52.50 0.0099 2.9433 0.323 21.39  11.1149557
#> 13           Witch  44.00 0.0017 3.3887 0.200 29.00  21.7175060
#> 14         Gurnard  42.97 0.0160 2.8151 0.266 17.67  23.7587503
#> 15          Plaice  65.32 0.0125 2.9425 0.122 22.22   4.8535417
#> 16      Starry ray  66.00 0.0107 2.9400 0.230 46.00   4.6666095
#> 17         Haddock  70.65 0.0092 3.0126 0.271 26.91   3.6046815
#> 18      Cuckoo ray  92.00 0.0026 3.2169 0.110 59.00   1.3242529
#> 19        Monkfish 106.00 0.0297 2.8410 0.180 61.00   0.7738734
#> 20             Cod 149.87 0.0081 3.0502 0.216 54.39   0.2080939
#> 21          Saithe 118.94 0.0085 3.0242 0.175 48.61   0.4999956
#>                b
#> 1  8.038261e+03
#> 2  7.322485e+02
#> 3  8.249558e+04
#> 4  1.756348e+02
#> 5  1.848772e+02
#> 6  2.599582e+02
#> 7  7.507404e+03
#> 8  5.379469e+03
#> 9  2.518504e+01
#> 10 6.446078e+01
#> 11 2.872764e+03
#> 12 7.492533e+01
#> 13 8.760097e+00
#> 14 2.423226e+01
#> 15 9.366251e+01
#> 16 1.774109e+00
#> 17 6.599579e+01
#> 18 4.997712e-02
#> 19 2.105690e-01
#> 20 7.656718e-01
#> 21 1.066107e+01
```

`NS_par` contains several variables that are required to set up the model: `Linf`, the von-Bertalanffy asymptotic length of each species (cm); `W_a` and `W_b`, length-weight conversion parameters; `k`, the von-Bertalanffy growth parameter; `Lmat` the length at which 50% of the individuals are mature (cm); `a`, the density-independent part of the hockey-stick recruitment function and `b`, the density-dependent part of the hockey-stick recruitment function.

`NS_tau` is the interaction matrix for the species in the model. Each row of the matrix represents a prey species and each column represents a predator species. If element `i,j` is equal to `1` it means that the `j`th

species predates on the `ith` species.

`NS_other` is other food for the North Sea.

Setting the species-independent parameters

The LeMans model is based on a discrete time discrete length system where there are n_s species and n_l length classes with the l_j being the lower length of the jth length class and l_{j+1} being the upper limit. The mid point of the jth length class is

$$L_j = l_j + \frac{l_{j+1} - l_j}{2}$$

for $j = 1 \dots n_l$.

To run LeMans model we must a number of species-independent parameters before it can be run. These parameters include: `nfish`, the number of species in the model; `nsc`, the number of length classes in the model and is set to 32 by default; and `l_bound`, `u_bound` and `mid`, which are the lower, upper and mid-points of the length classes respectively. The values of these parameters can be calculated by running:

```
nfish <- nrow(NS_par)
nsc <- 32
maxsize <- max(NS_par$Linf)*1.01 # the maximum size is 1% bigger than the largest Linf.
l_bound <- seq(0, maxsize, maxsize/nsc); l_bound <- l_bound[-length(l_bound)]
u_bound <- seq(maxsize/nsc, maxsize, maxsize/nsc)
mid <- l_bound+(u_bound-l_bound)/2
```

`phi_min` is the time step of the model and can either be specified explicitly, with a default of 0.1, or calculated (see `calc_phi` in the next section). The LeMans model also has a numerical precision parameter `eps`, which is set to `1e-05` by default.

Setting the species-specific parameters

Size, growth and maturity

In addition to the species-independent parameters described above, the LeMans model also requires a number of species-specific parameters. As a minimum the model requires:

```
Linf <- NS_par$Linf # the von-Bertalanffy asymptotic length of each species (cm)
W_a <- NS_par$W_a # length-weight conversion parameter
W_b <- NS_par$W_b # length-weight conversion parameter
k <- NS_par$k # the von-Bertalanffy growth parameter
Lmat <- NS_par$Lmat # the length at which 50\% of individuals are mature (cm)
```

The length-weight equation is defined for species i in length class j as

$$W_{j,i} = a_i L_j^{b_i}.$$

In `LeMaRns` a_i is represented by `W_a` and b_i is represented by `W_b`. The LeMans model assumes that fish grow continuously throughout their lives, with the length of species i at age t being

$$L_{\infty,i}(1 - e^{-k_i(t - t_{0,i})}),$$

where $L_{\infty,i}$ is the asymptotic length (cm) of the ith species, k_i is the von Bertalanffy growth rate and $t_{0,i}$ is the theoretical age at which the ith species would be at length 0. In `LeMaRns` $t_{0,i}$ is not required, $L_{\infty,i}$ is represented by `Linf` and k_i is represented by `k`.

The amount of time t that species i spends in length class j is defined as:

$$t_{j,i} = \frac{1}{k_i} \times \log\left(\frac{L_{\infty,i} - l_j}{L_{\infty,i} - l_{j+1}}\right). \quad (1)$$

Assuming that individuals are evenly distributed across the length class, the proportion of individuals of species i that leave length class j due to growth over time δt is

$$\phi_{j,i} = \frac{\delta t}{t_{j,i}}.$$

In `LeMaRns` $\phi_{j,i}$, $\forall i, j$, is represented by `phi`. In the example below we set `phi_min` equal to 0.1:

```
tmp <- calc_phi(k, Linf, nsc, nfish, u_bound, l_bound, calc_phi_min=FALSE, phi_min=0.1)
phi <- tmp$phi
phi_min <- tmp$phi_min
```

The amount that a fish of species i in length class j will grow according to the von Bertalanffy growth curve in a time step, δt, is

$$\delta L_{j,i} = (L_{\infty,i} - L_{j,i})(1 - \exp(-k_i \delta t)).$$

This is then converted into weight increments, $\delta W_{j,i} = a_i(\delta L_{j,i})^{b_i}$, and then divided by the growth efficiency,

$$G_{j,i} = \left(1 - \left(\frac{W_{j,i}}{W_{\infty,i}}\right)^{\pi}\right) \times g_0,$$

where $W_{\infty,i}$ is the weight of a fish of species i of length $L_{\infty,i}$, π is the rate at which the growth efficiency decays and g_0 is the growth efficiency of a fish of length zero. The amount of food required for the fish to grow according von Bertalanffy growth curve in a time step is then

$$I_{j,i} = \frac{\delta W_{j,i}}{G_{j,i}}.$$

This is calculated using the `calc_ration_growthfac` function. This calculates: `ration`, the food required (g) for a fish of average size in each length class to grow according von Bertalanffy growth curve in a time step; `sc_Linf`, the length class at which each species reaches `Linf`; `wgt`, a matrix with dimensions `nsc` and `nfish` representing the weight of each species in each length class; and `g_eff`, a matrix with dimensions `nsc` and `nfish` representing the growth efficiency of each species in each length class.

```
tmp <- calc_ration_growthfac(k, Linf, nsc, nfish, l_bound, u_bound, mid, W_a, W_b
                             , phi_min)
ration <- tmp$ration
sc_Linf <- tmp$sc_Linf
wgt <- tmp$wgt
g_eff <- tmp$g_eff
```

For species i in length class j, the proportion of individuals that are mature is defined as:

$$M_{j,i} = \frac{1}{1 + \exp\{-\kappa_i(L_j - L_i^{(mat)})\}}$$

where $L_i^{(mat)}$ is the length at which 50% of individuals are mature and κ_i is the rate of change from immaturity to maturity. In `LeMaRns` $L_i^{(mat)}$ is represented by `Lmat` and κ_i is represented by `kappa`. In Thorpe et al.

(2015), κ_i is equal to 10 for all species. The proportion of individuals that are mature may thus be calculated by running:

```
mature <- calc_mature(Lmat, nfish, mid, kappa=rep(10, nfish), sc_Linf)
```

In the LeMans model, fish may feed on each other and on other food (g), which is referred to in LeMaRns as other.

```
other <- NS_other
```

Recruitment

There are several different options for recruitment in LeMaRns. The recruitment function implemented in Hall et al. (2006) is the Ricker stock-recruitment function, which gives a dome-shaped relationship between spawning stock biomass (SSB) and the number of recruits and is defined,

$$R_i = \alpha_i S_i e^{-\beta_i S_i},$$

where the species-specific parameters α_i and β_i are the density-dependent and density-independent parameters respectively (Ogle 2016) and S_i is the SSB in kilo-tonnes, i.e.

$$S_i = \frac{1}{10^9} \sum_{j=1}^{n_l} M_{j,i} N_{j,i} W_{j,i},$$

where $N_{j,i}$ is the number of individuals of species i in length class j.

In LeMaRns α_i is represented by a and β_i is represented by b. Below is an example with α_i set to 1.5 and β_i set to 0.5:

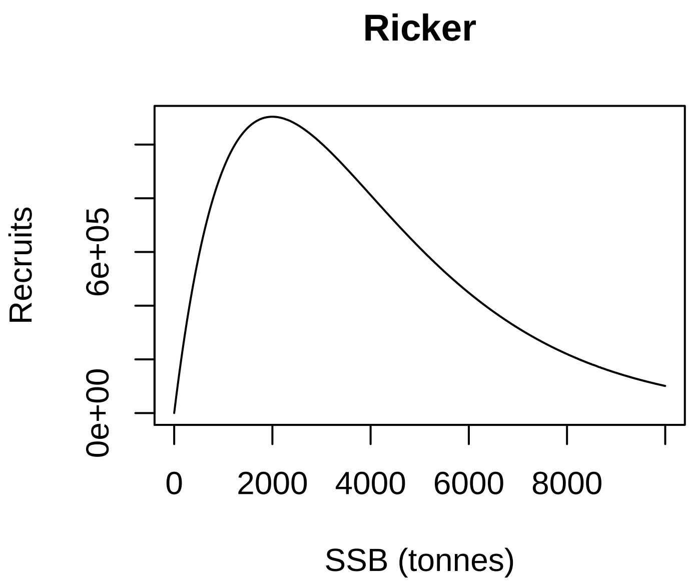

It is also possible to implement the Beverton-Holt recruitment function:

$$R_i = \frac{\alpha_i S_i}{1 + \beta_i S_i},$$

a `linear` recruitment function:

$$R_i = \alpha_i S_i,$$

a `hockey-stick` recruitment function:

$$R_i = \min(\alpha_i S_i, R_{max,i})$$

where $R_{max,i}$ is the maximum recruitment, or `constant` recruitment R_i. Below are examples of these recruitment functions:

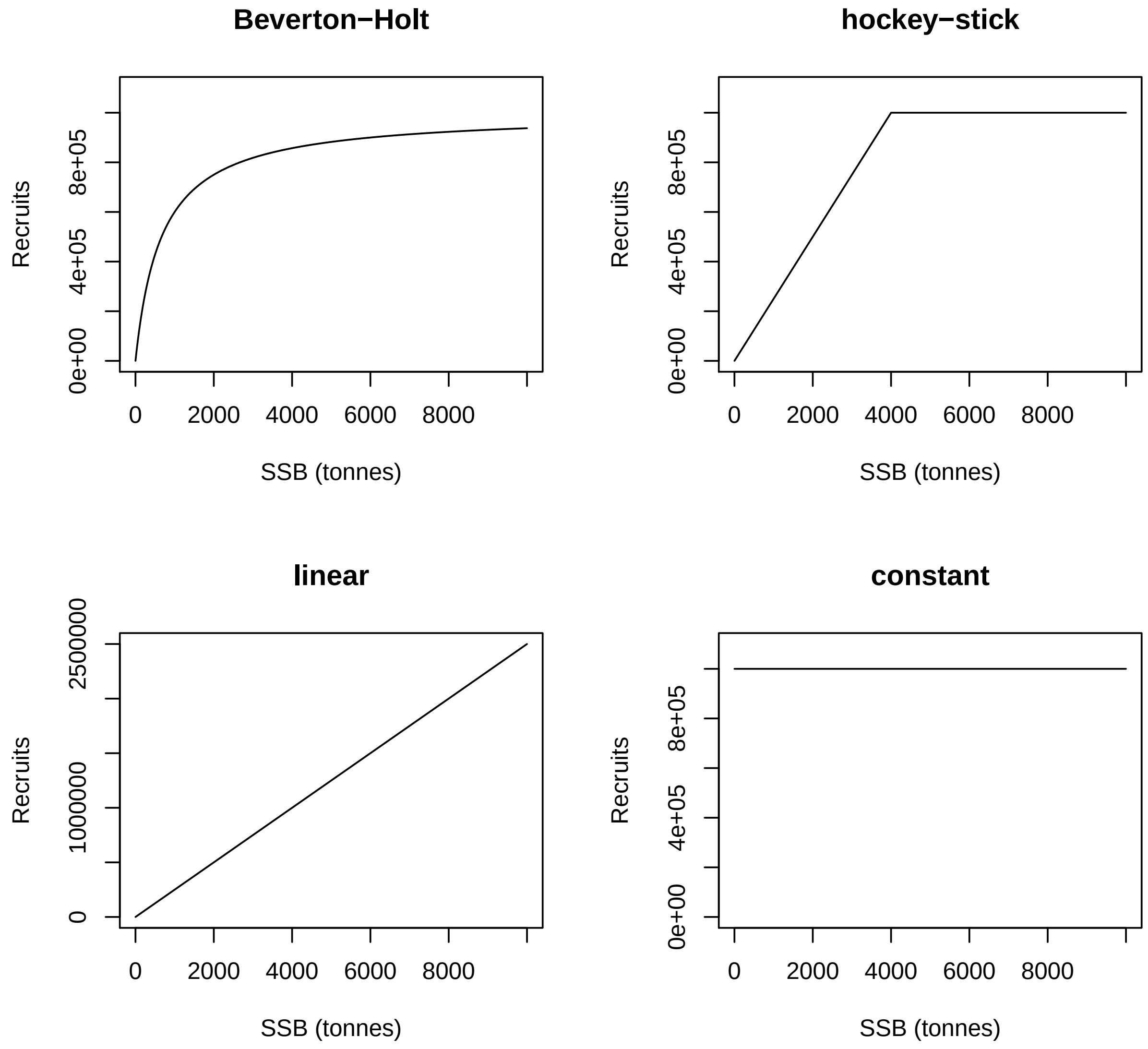

To set up the `hockey-stick` function:

```
stored_rec_funs <- get_rec_fun(rep("hockey-stick", nfish))
recruit_params <- do.call("Map", c(c, list(a=NS_par$a, b=NS_par$b)))
```

For more details about recruitment see `help(calc_recruits)`.

Mortality

There are three types of mortality in the LeMans model: background mortality ($M1$), predation mortality ($M2$) and fishing mortality (F).

In `LeMaRns`, there are three different options for background mortality functions: `std_RNM`, `constant` and `linear`. `std_RNM` is the default option and gives a constant mortality rate, defined by `Nmort`, for the largest length classes and zero for the smallest. When using the `std_RNM` option, the parameter `prop` is used to define which length classes are considered to have a non-zero mortality rate. `constant` gives a constant mortality rate, defined by `Nmort`, for all length classes and `linear` gives `M1=0` for the first length class, followed by a linear increase in $M1$ up to and including the `sc_Linf`-th length class where `M1=Nmort`.

Below gives examples of the different `M1` functions for a species with an L_∞ of 125:

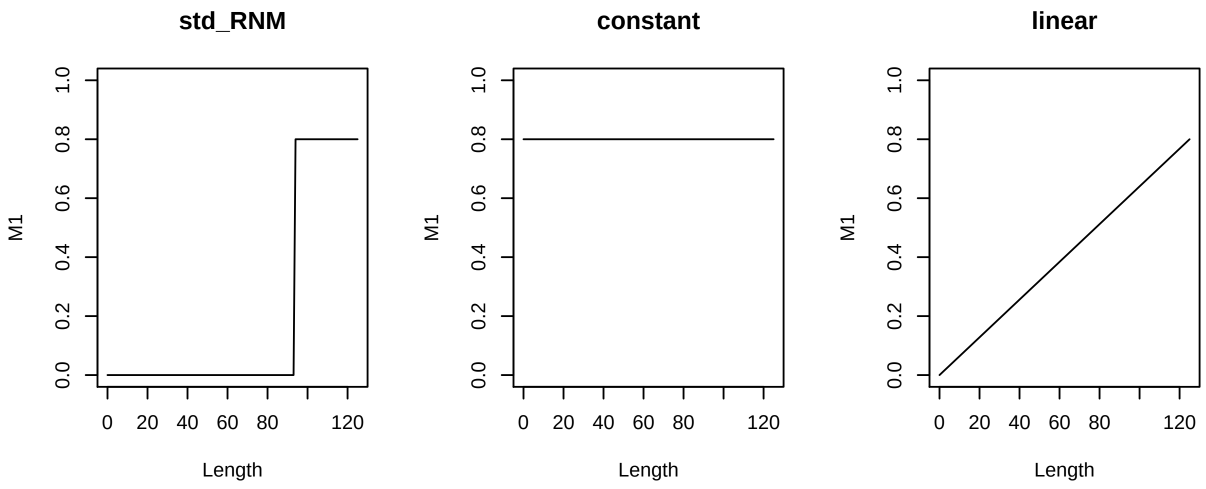

To set up the `std_RNM` natural mortality function with a `prop` of 0.75 and an `Nmort` of 0.8:

```
M1 <- calc_M1(nsc, sc_Linf, phi_min,
              natmort_opt=rep("std_RNM", length(sc_Linf)),
              Nmort=rep(0.8, length(sc_Linf)),
              prop=rep(0.75, length(sc_Linf)))
```

The species in the model interact with each other via predator-prey interactions. The prey weight preference w_p for a predator of weight w is defined as:

$$p(w, w_p) = \exp\left\{-\frac{\left(\log_{10}\left(\frac{w_p}{w}\right) - \mu_p\right)^2}{2\sigma_p}\right\},$$

where μ_p is the preferred predator-prey mass ratio and σ_p is the width of the weight preference function. In `LeMaRns` μ_p is represented by `pred_mu`, σ_p is represented by `pred_sigma` and prey weight preferences are calculated by running:

```
prefs <- calc_prefs(pred_mu=-2.25, pred_sigma=0.5, wgt, sc_Linf)
```

The suitability of prey of weight w_p and species j for a predator of weight w of species i is defined as:

$$U(w, w_p, i, j) = p(w, w_p)\tau_{i,j},$$

where $\tau_{i,j}$ is a value between 0 and 1 that represents the vulnerability of prey species j to predator species i (Hall et al. 2006). In `LeMaRns` prey suitability is calculated by running:

```
suit_M2 <- calc_suit_vect(nsc, nfish, sc_Linf, prefs, NS_tau)
```

This returns a list object of length `nfish`. Each element in the list is an array of dimensions `nsc`, `nsc` and `nfish` containing a value between 0 and 1 that represents a combination of prey preference and prey suitability for each species and length class, which is then used to determine predation mortality.

In the LeMans model, fishing mortality is dependent on effort and gear catchability. The instantaneous fishing mortality for species i of in length class j is

$$F_{j,i} = \sum_{k=1}^{H} e_k q_{i,k}(L_j),$$

where e_k is the effort of the kth gear, for $k = 1 \ldots H$, and $q_{i,k}(L_j)$ is the catchability of a fish of length L_j and species i by gear k. Effort is added to the model during initialisation (see the next section for more details), whilst catchability is added when setting up the model. `LeMaRns` provides three in-built options for catchability curves: `logistic`, `log_gaussian` and `knife-edge`, but it also allows users to input their own catchability values using the `custom` input. The `logistic` curve is defined as:

$$q(L) = \frac{1}{1 + \exp(-\eta(L - L_{50}))},$$

where η is the steepness of the slope of the catchability curve and L_{50} is the length at 50% of the maximum catchability. This function gives a catchability of zero for the smallest length classes, a catchability of 1 for the largest length classes, and a smooth increase in catchability from zero to one around l_{50}. An example of a `logistic` catchability curve is shown below with an η of 0.25 and an L_{50} of 50:

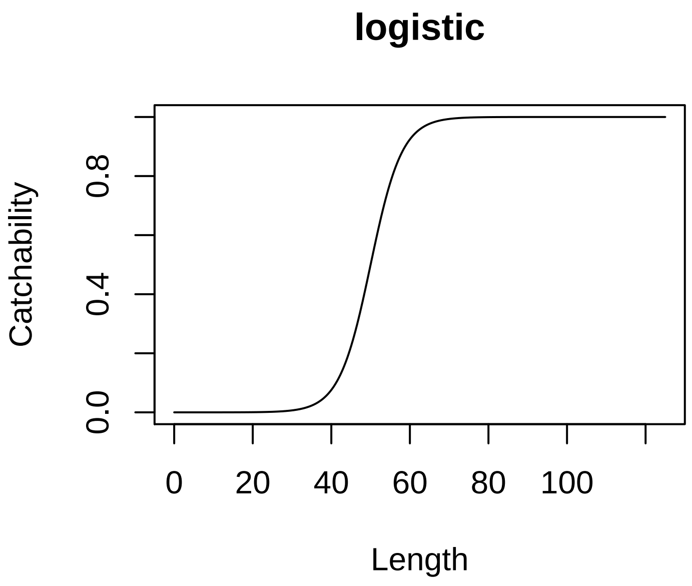

The `log_gaussian` catchability function is defined as:

$$q(L) = \frac{1}{L\sigma\sqrt{2\pi}} \exp\left\{-\frac{(\log(L) - \log(\mu_{50}))^2 + \log(\mu_{50}) - \sigma^2}{2\sigma^2}\right\}$$

where z is ..., μ_{50} is ... and σ is the standard deviation of the catchability curve. This function gives a dome-shaped relationship between length and catchability. An example of a `log-gaussian` catchability curve is shown below with a μ_{50} of 50 and a σ of 1.

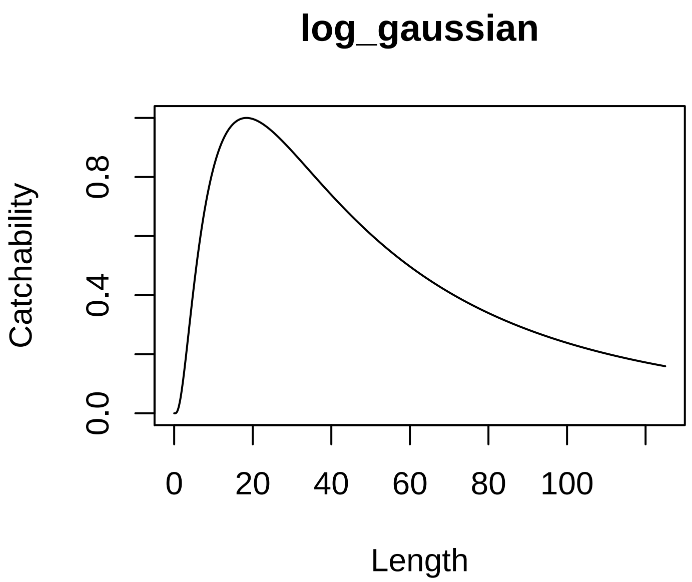

The `knife-edge` function is

$$q(L) = \begin{cases} 1 & \text{if } L \geq L_\mu \\ 0 & \text{otherwise,} \end{cases}$$

where L_μ is the smallest length that is caught by the gear. An example of a `knife-edge` catchability curve is shown below with a L_μ of 50:

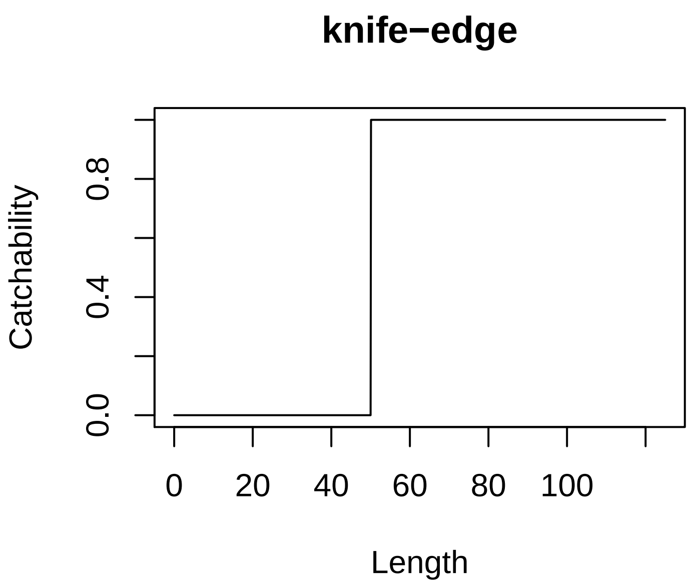

We provide a case study that explores the use of multiple different gears at the end of the vignette, but for now we will set up a gear for each species that follows a `logistic` catchability curve with a L_{50} at `Lmat`. In `LeMaRns` η is represented by `eta` and L_{50} is represented by `L50`:

```
Qs <- calc_Q(nsc=nsc, nfish=nfish, mid=mid, l_bound=l_bound, u_bound=u_bound,
             species_names=NS_par$species_names,curve=rep("logistic", nfish),
             species=NS_par$species_names, max_catchability=rep(1, nfish),
             gear_name=NS_par$species_names,eta=rep(0.25, nfish), L50=Lmat)
```

`LeMans_param` objects

A `Lemans_param` object can be used to store the model parameters (see `help(Lemans_param)` for further details). It is possible to create a `Lemans_param` object by running:

```
NS_params <- new("LeMans_param",
                 nsc=nsc,
                 nfish=nfish,
                 phi_min=phi_min,
                 l_bound=l_bound,
                 u_bound=u_bound,
                 mid=mid,
                 species_names=NS_par$species_names,
                 Linf=Linf,
                 W_a=W_a,
                 W_b=W_b,
                 k=k,
                 Lmat=Lmat,
                 mature=mature,
                 sc_Linf=sc_Linf,
                 wgt=wgt,
                 phi=phi,
                 ration=ration,
                 other=other,
```

```
                      M1=M1,
                      suit_M2=suit_M2,
                      Qs=Qs,
                      stored_rec_funs=stored_rec_funs,
                      eps=1e-05,
                      recruit_params=recruit_params)
```

Alternatively a `Lemans_param` object can be created using the `LeMansParam` function. This function can be used in several ways. For example, the parameters can be given individually:

```
NS_params <- LeMansParam(species_names=NS_par$species_names,
                              Linf=Linf, k=k, W_a=W_a, W_b=W_b,
                              Lmat=Lmat, tau=NS_tau,
                              recruit_params=list(a=NS_par$a, b=NS_par$b),
                              eta=rep(0.25, 21), L50=Lmat)
```

the species parameters can be provided as a `data.frame` (referred to as `df`):

```
NS_params <- LeMansParam(NS_par, tau=NS_tau, eta=rep(0.25, 21), L50=NS_par$Lmat, other=NS_other)
```

and/or the gear types can be provided as a `data.frame` (referred to as `gdf`; see the gear examples below). As a minimum the species data frame must contain `Linf`, `k`, `W_a`, `W_b` and `Lmat`. It is also possible to include `species_names`, `kappa`, `rec_fun`, `natmort_opt`, `Nmort` and `prop`. Any additional variables are assumed to be recruitment parameters and a warning is shown to highlight this, i.e.

```
#> Warning in .local(df, gdf, ...): The following columns of df do not match any of the species argumen
#> a, b
```

Running the model

The LeMans model works by projecting the number of individuals in each length class for each species forward in time. `N` is therefore a `nsc` by `nfish` matrix where `N[j,i]` is the number of individuals of species `i` that are in length class `j`. We will refer to $N_{j,i,t}$ as the number of individuals of species i in length class j and time t.

Initialising the model

Given all of the aforementioned parameters, the minimum requirement for the model to be run is an initial matrix `N`. `N` can be calculated using an `intercept` of e.g. 1e10 and a `slope` of e.g. -5 by running:

```
N <- get_N0(nsc, nfish, mid, wgt, sc_Linf, intercept=1e10, slope=-5)
```

In this function the total number of individuals in the community in each length class is equal to `intercept*mid^slope`. Within each length class, the number of individuals of each species is determined using the proportion of each species' biomass that is found in that length class. We can now plot the initial size spectrum of the model:

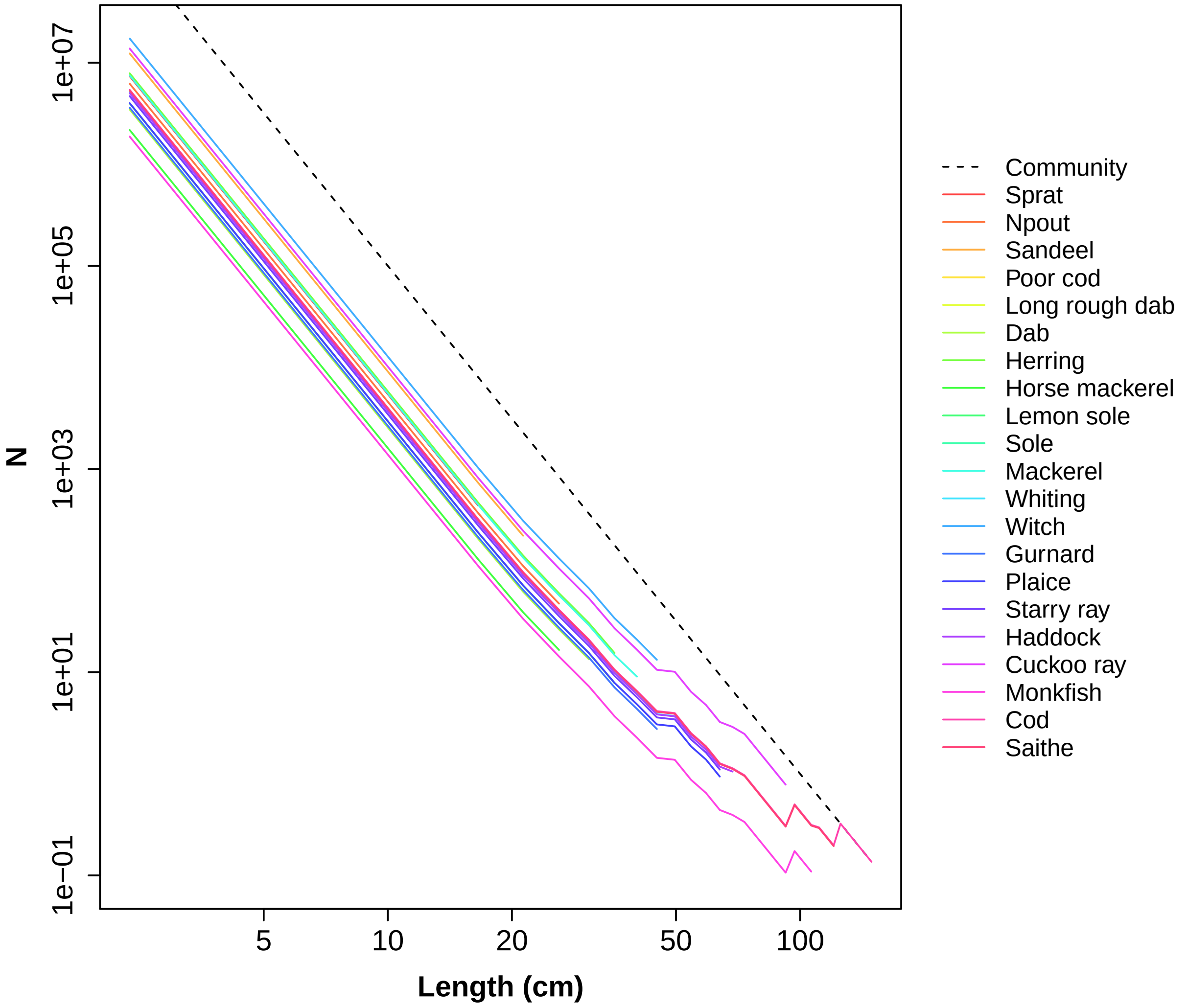

The fishing mortality, `Fs`, must also be defined. To do this we need to define the `effort` for each of the gears `Qs`. In this example we will set the effort to be 0.5 for all species:

```
effort <- rep(0.5, dim(Qs)[3])
Fs <- matrix(0, nsc, nfish)
for (j in 1:length(effort)) {
   Fs <- Fs+effort[j]*Qs[,,j]
}
```

The model run

The model is run for many time steps, with the length of each time step being equal to `phi_min`. At each time step three processes occur: recruitment, mortality and growth.

Recruitment

In Thorpe et al. (2015) and Hall et al. (2006) recruitment only occurs in the first time step of a new year. The number of individuals after the recruitment phase of the model $N'_{j,i,t}$ is thus defined as $N'_{j,i,t} = N_{j,i,t-1}$

for $j = 2, \ldots, n_l$ and

$$N'_{1,i,t} = N_{1,i,t-1} + R_{i,t},$$

where $R_{i,t}$ is the number of recruits of species i at time t. When the recruitment phase is implemented in `LeMaRns` the SSB is first calculated in grams and then the number of recruits is calculated:

```
SSB <- calc_SSB(mature, N, wgt)
R <- calc_recruits(SSB, stored_rec_funs, recruit_params)
N[1, ] <- N[1,]+R
```

Mortality

The number of individuals after the mortality phase of the model $N''_{j,i,t}$ is defined as:

$$N''_{j,i,t} = N'_{j,i,t} \exp(-(M1_{j,i} + M2_{j,i,t} + F_{j,i,t})\delta t).$$

where $M1$ is the background mortality, $M2$ is the predation mortality and F is the fishing mortality (represented by `Fs` in `LeMaRns`). The predation mortality of a fish of species i in length class j is

$$M2_{j,i,t} = \sum_{m=1}^{n_s} \sum_{n=1}^{n_l} I_{j,i} N'_{j,i,t} \frac{U(W_{n,m}, W_{j,i}, m, i)}{\sum_{k=1}^{n_s} \sum_{l=1}^{n_l} U(W_{n,m}, W_{l,k}, m, k) W_{l,k} N_{l,k} + o}$$

where o is other food.

The catch of species i in length class j is

$$C_{j,i,t} = N'_{j,i,t}(1 - \exp(-F_{j,i,t}\delta t)).$$

In `LeMaRns`, `M2` is calculated and then added to `Fs`, `M1` and a numerical stability parameter known as `eps`. Total mortality `Z` and the `Catch` for each species and length class (g) is calculated by running:

```
M2 <- calc_M2(N, ration, wgt, nfish, nsc, other, sc_Linf, suit_M2)
Z <- Fs+M1+M2+1e-05
Catch <- (Fs/Z)*N*(1-exp(-Z))*wgt
N <- N*exp(-Z)
```

Growth

The number of individuals after the growth phase $N_{j,i,t}$ is defined as:

$$N_{j,i,t} = \begin{cases} N''_{j,i,t}(1 - \phi_{j,i}) & \text{if } j = 1 \\ N''_{j,i,t}(1 - \phi_{j,i}) + N''_{j-1,i,t}\phi_{j-1,i} & \text{otherwise} \end{cases}$$

In `LeMaRns`, growth is calculated by running:

```
N <- calc_growth(N, phi, nfish, nsc)
```

`run_LeMans` function

Alternatively, the `run_LeMans` function can be used to run a dynamic version of the model over a given number of years (as defined by `years`).

There are several ways to use the `run_LeMans` function. All of the parameters can be added to the function explicitly. To do this, we first need to define the initial population and set the correct number of time steps. Below we run the model for 50 years:

```
N0 <- get_N0(nsc, nfish, mid, wgt, sc_Linf, intercept=1e10, slope=-5) # initialise N
years <- 50 # run for 10 years
tot_time <- years*phi_min # calculate the total number of time steps
```

We then need to define the fishing mortality. In the example below we fish for the duration of the model run with e_k equal to 0.5 for all gears:

```
effort <- matrix(0.5, tot_time, dim(Qs)[3])
# Calculate F
Fs <- array(0, dim=c(nsc, nfish, tot_time))
for (j in 1:ncol(effort)) {
  for (ts in 1:tot_time) {
    Fs[,,ts] <- Fs[,,ts]+effort[ts, j]*Qs[,,j]
  }
}
```

We are then in a position to run the model:

```
model_run <- run_LeMans(N0=N0, tot_time=tot_time, Fs=Fs, nsc=nsc, nfish=nfish, phi_min=phi_min,
                        mature=mature, sc_Linf=sc_Linf, wgt=wgt, phi=phi, ration=ration,
                        other=other, M1=M1, suit_M2=suit_M2, stored_rec_funs=stored_rec_funs,
                        recruit_params=recruit_params, eps=1e-05)
```

Alternatively we can pass a `LeMans_param` object to the `run_LeMans` function:

```
model_run <- run_LeMans(NS_params, N0=N0, Fs=Fs, tot_time=tot_time)
```

Another alternative is to use the `run_LeMans` function to initialise the model as well. To do this, `effort` must be defined as a `years` by `dim(Qs)[3]` matrix where the fishing effort remains the same for a whole year:

```
effort_mat <- matrix(0.5, years, dim(Qs)[3])
model_run <- run_LeMans(NS_params, years=50, effort=effort_mat)
```

Exploring the outputs

The `run_LeMans` function returns a `LeMans_output` object. A `LeMans_output` object has four slots: `N`, an array with dimensions `nsc`, `nfish` and `tot_time` representing the number of individuals in each length class at each time step of the model; `Catch`, an array with dimensions `nsc`, `nfish` and `tot_time` representing the biomass (g) of each species that is caught in each length class at each time step of the model; `M2`, an array with dimensions `nsc`, `nfish` and `tot_time` representing the predation mortality of each species in each length class at each time step of the model; and `R`, a matrix with dimensions `tot_time` and `nfish` representing the number of recruits of each species at each time step of the model.

The `LeMaRns` package includes a number of in-built functions that enable users to explore the outputs of a model run in more detail. These functions can be used to calculate and plot community and species-specific biomass and SSB, as well as several ecosystem indicators including the Large Fish Indicator (LFI), Mean Maximum Length (MML), Typical Length (TyL) and Length Quantiles (LQ). There are also functions to calculate Catch Per Unit Effort (CPUE) and Catch Per Gear (CPG).

Biomass

The biomass of each species at time t is defined as:

$$B_{i,t} = \sum_{j=1}^{n_l} N_{j,i,t} W_{j,i}.$$

In LeMaRns the biomass of each species can be calculated using the get_biomass function. There are several ways in which this function can be used. For example, users can pass N and wgt to the function directly:

```
biomass <- get_biomass(N=N, wgt=wgt)
```

or they can provide both LeMans_param and a LeMans_output objects:

```
biomass <- get_biomass(inputs=NS_params, outputs=model_run)
```

Options also exist that allow users to specify the species and time_steps they would like the to calculate the biomass for. species can be specified either as a numeric vector:

```
biomass <- get_biomass(inputs=NS_params, outputs=model_run, time_steps=1:500, species=1:4)
```

or as a character vector:

```
biomass <- get_biomass(inputs=NS_params, outputs=model_run, time_steps=1:500,
                       species=c("Herring", "Whiting", "Haddock", "Cod"))
```

If more than one species is provided, the get_biomass() function returns a matrix with dimensions length(time_steps) by length(species) where the i, jth element is the biomass (g) of the jth species in the ith time_steps. If only one species is provided, the function returns a numeric vector of length length(time_steps).

A similar set of methods can be used to plot the biomass of each species using the plot_biomass function. For example, running either:

```
plot_biomass(N=N, wgt=wgt, time_steps=1:500, species=1:4)
```

or

```
plot_biomass(inputs=NS_params, outputs=model_run, time_steps=1:500, species=1:21)
```

returns the following plot:

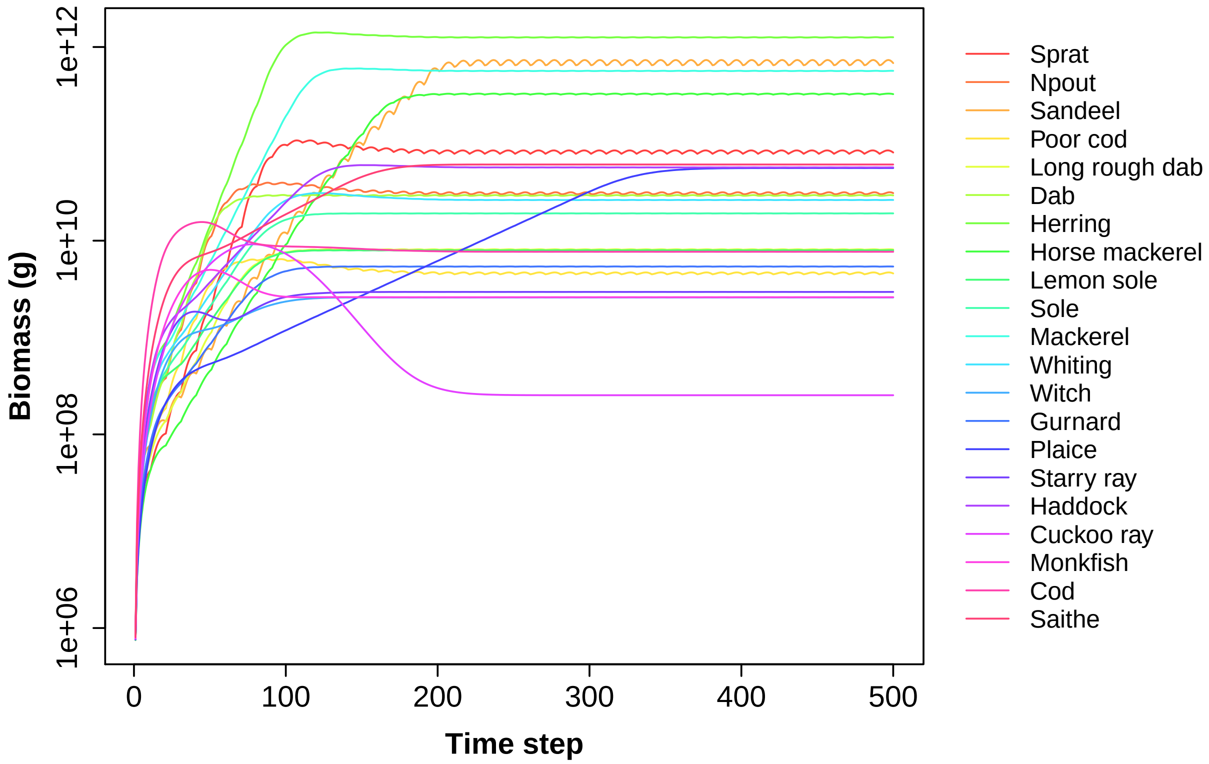

Alternatively, users may pass the outputs of `get_biomass` to the `plot_biomass` function directly as shown below:

```
biomass <- get_biomass(inputs=NS_params, outputs=model_run)
plot_biomass(biomass=biomass, time_steps=1:500, species=1:4)
```

The `full_plot_only` option allows users to specify whether individual plots should also be produced for each species. For example, the following plots are returned by setting `full_plot_only` to `FALSE`:

```
plot_biomass(inputs=NS_params, outputs=model_run, time_steps=1:500, species=1:4,
             full_plot_only=FALSE)
```

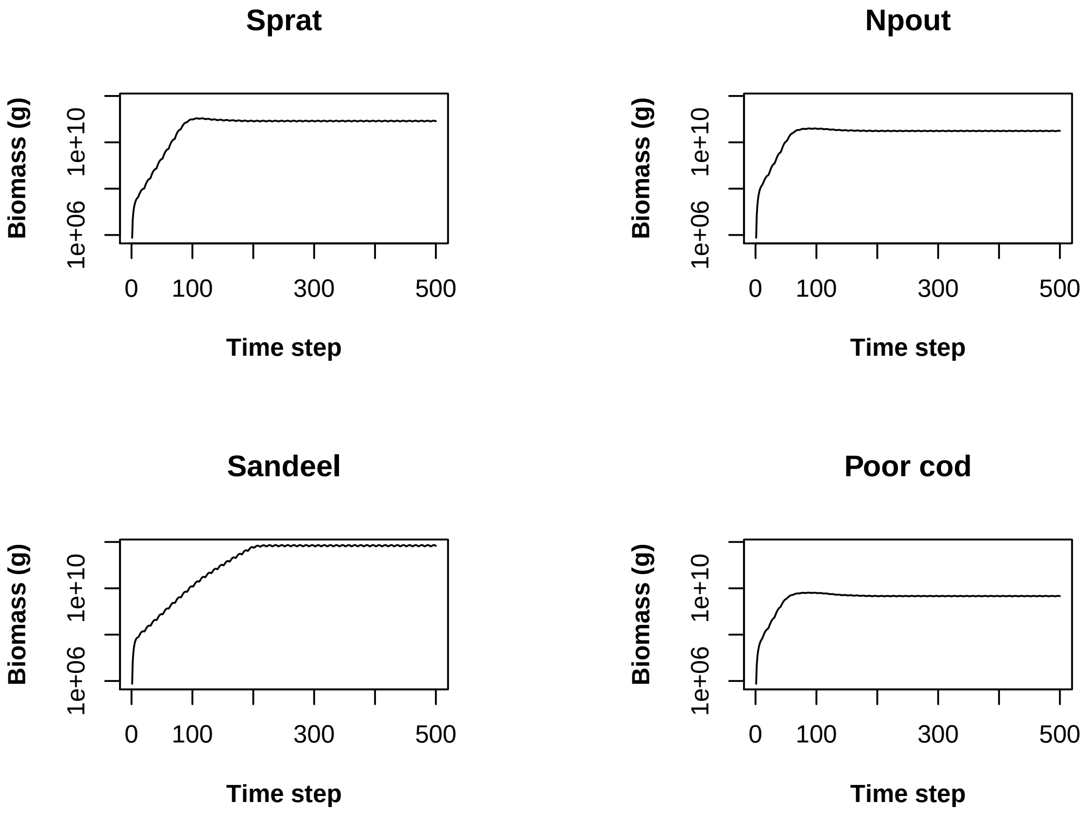
Sprat
Npout
Sandeel
Poor cod
Biomass (g)
1e+06
1e+10
0
100
300
500
Time step

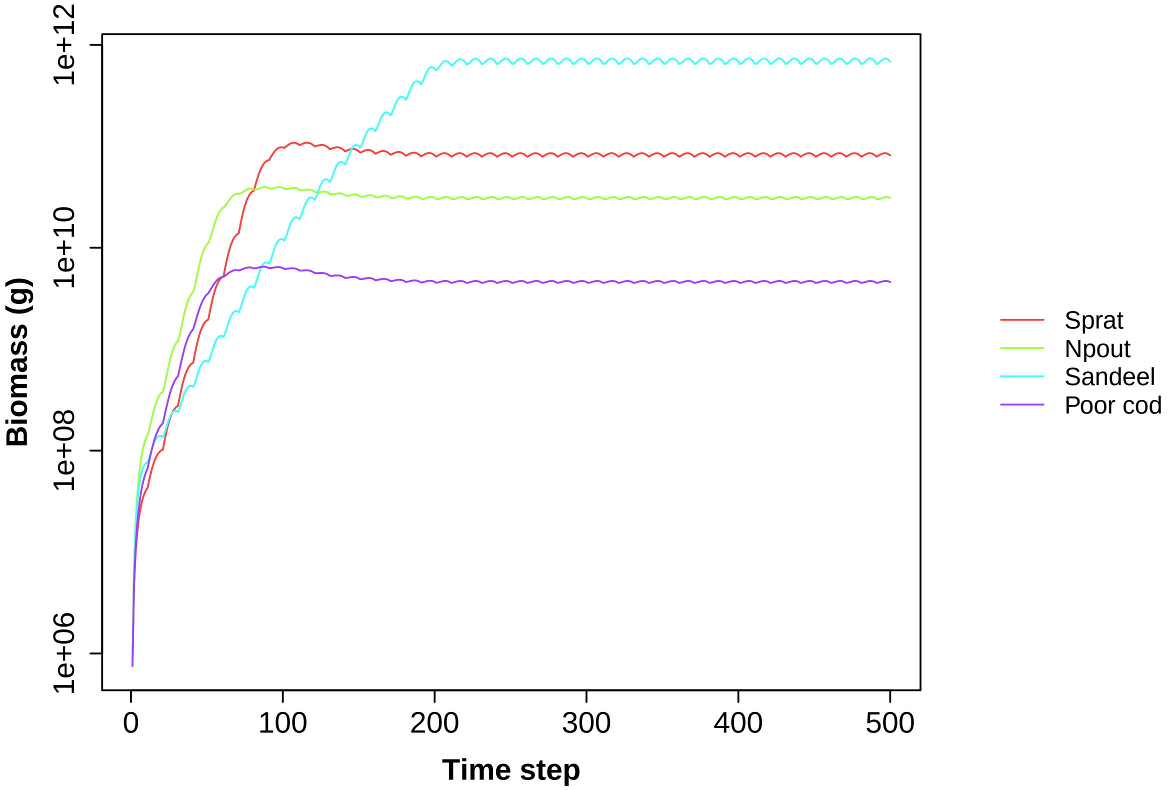

Note that if a `LeMans_param` object is passed to the `plot_biomass` function, the plot will be labelled with the correct species names. However, if a `LeMans_param` object is not provided, the species will be labelled as "Species 1", "Species 2" etc. unless users specify `species_names` directly.

Spawning Stock Biomass (SSB)

The SSB of each species at time t is defined as:

$$SSB_{i,t} = \sum_{j=1}^{n_l} M_{j,i} N_{j,i,t} W_{j,i}$$

where $M_{j,i}$ is the proportion of mature individuals of species i in length class j.

In `LeMaRns` the SSB of each species can be calculated using the `get_SSB` function and plotted using the `plot_SSB` function, both of which work in exactly the same way as `get_biomass` and `plot_biomass` as described above. For example, species-specific SSB may be calculated and plotted by running:

```
SSB <- get_SSB(inputs=NS_params, outputs=model_run, time_steps=1:500, species=1:21)
plot_SSB(SSB=SSB, time_steps=1:500, species=1:21, species_names=NS_params@species_names)
```

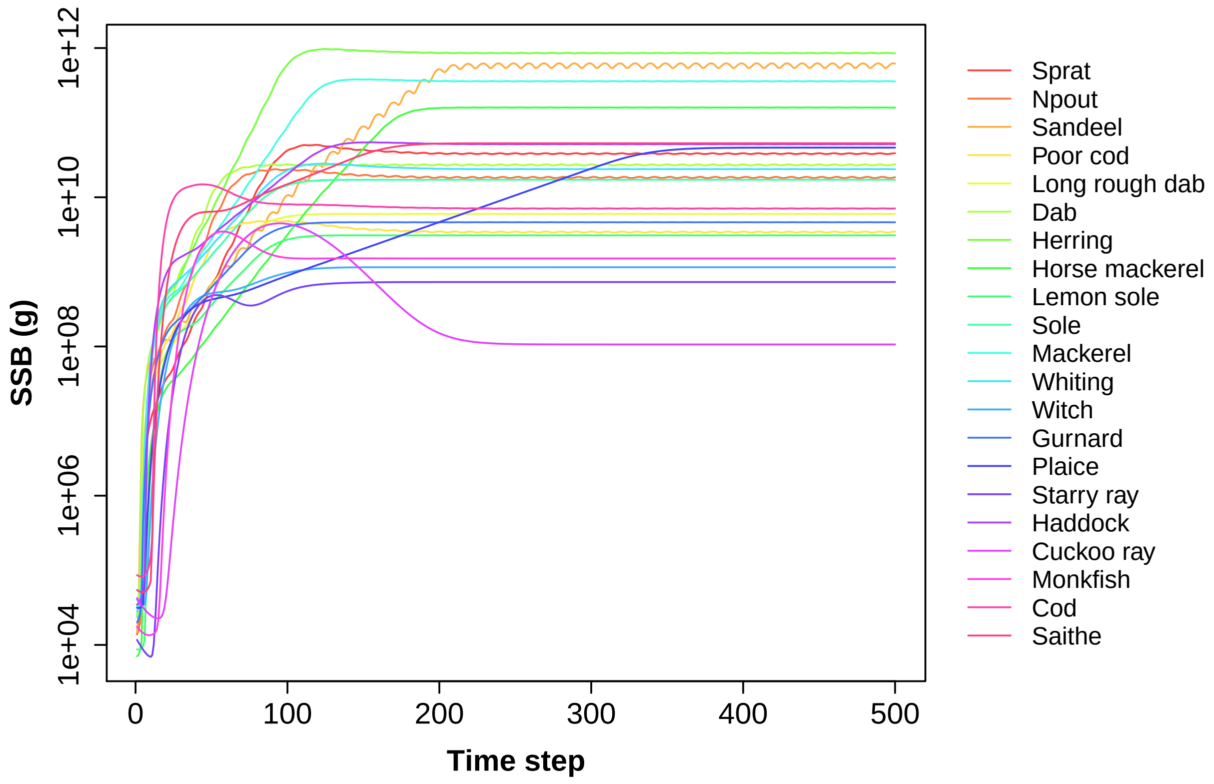

Indicators

As previously mentioned, `LeMaRns` also includes in-built functions that can be used to quantify and plot several proposed indicators of ecosystem health, including the Large Fish Indicator (LFI), Mean Maximum Length (MML), Typical Length (TyL) and Length Quantiles (LQ).

The `LFI` represents the proportion of the biomass with a length larger than L_{LFI} (cm) at time t:

$$LFI_t = \frac{\sum_{i=1}^{n_s} \sum_{j=1}^{n_l} N_{j,i,t} W_{j,i} I(L_i \geq L_{LFI})}{\sum_{i=1}^{n_s} B_{i,t}},$$

where I is an indicator function that takes a value of one if $L_i \geq L_{LFI}$ is `TRUE` and zero if it is `FALSE`. In `LeMaRns` L_{LFI} is represented by `length_LFI` and is set to 40cm by default, but it can take any length and can also take on multiple values if required (see below for example).

`MML` represents the biomass-weighted mean of $L_{\infty,i}$ at time t:

$$MML_t = \frac{\sum_{i=1}^{n_s} B_{i,t} L_{\infty,i}}{\sum_{i=1}^{n_s} B_{i,t}}$$

where $L_{\infty,i}$ represents the asymptotic length for the ith species in a community of m species and is denoted as `Linf` in `LeMaRns`.

`TyL` represents the biomass-weighted geometric mean length of the community at time t:

$$TyL_t = \exp\left[\frac{\sum_{i=1}^{n_s} N_{j,i,t} W_{j,i} \log(L_j)}{\sum_{i=1}^{n_s} B_{i,t}}\right]$$

`LQ` represents the length $L_{p,t}$ at which biomass exceeds a given proportion p of the total biomass at time t. This is calculated by solving:

$$p = \frac{\sum_{i=1}^{n_s} \sum_{j=1}^{n_l} N_{j,i,t} W_{j,i} I(L_i \leq L_{p,t})}{\sum_{i=1}^{n_s} B_{i,t}}$$

for $L_{p,t}$. In `LeMaRns` p is represented by `prob` and is set to 0.5 by default, but it can take any value between 0 and 1 and can also take multiple values if required (see below for example).

The LFI, MML, TyL and LQ can be calculated using the `get_LFI`, `get_MML`, `get_TyL` and `get_LQ` functions respectively, which again work in a similar way to the `get_biomass` function. For example, each indicator may be calculated by running:

```
LFI <- get_LFI(inputs=NS_params, outputs=model_run, time_steps=1:500, species=1:21,
               length_LFI=c(30, 40))
MML <- get_MML(inputs=NS_params, outputs=model_run, time_steps=1:500, species=1:21)
TyL <- get_TyL(inputs=NS_params, outputs=model_run, time_steps=1:500, species=1:21)
LQ <- get_LQ(inputs=NS_params, outputs=model_run, time_steps=1:500, species=1:21,
             prob=c(0.5, 0.95))
```

If more than one `length_LFI` is specified, the `get_LFI` function will return a matrix with dimensions `length(time_steps)` by `length(length_LFI)` where the i,jth element is the LFI given by the jth `length_LFI` in the ith `time_steps`. If only one species is provided, the function will return a numeric vector of length `length(time_steps)`. The same is also true when more than one `prob` is passed to the `get_LQ` function. Both the `get_MML` and `get_TyL` functions always return a numeric vector of length `length(time_steps)`.

The LFI, MML, TyL and LQ can be plotted using the `plot_LFI`, `plot_MML`, `plot_TyL` and `plot_LQ` functions respectively. Users can either provide `LeMans_param` and `LeMans_output` objects to the plot functions:

```
plot_LFI(inputs=NS_params, outputs=model_run, time_steps=1:500, species=1:21,
               length_LFI=c(30, 40))
plot_MML(inputs=NS_params, outputs=model_run, time_steps=1:500, species=1:21)
plot_TyL(inputs=NS_params, outputs=model_run, time_steps=1:500, species=1:21)
plot_LQ(inputs=NS_params, outputs=model_run, time_steps=1:500, species=1:21,
             prob=c(0.5, 0.95))
```

or they can provide the outputs of `get_LFI`, `get_MML`, `get_TyL` and `get_LQ` to the plot functions directly:

```
plot_LFI(LFI, length_LFI=c(30, 40))
plot_MML(MML)
plot_TyL(TyL)
plot_LQ(LQ, prob=c(0.5, 0.95))
```

In both cases, the following plots will be returned:

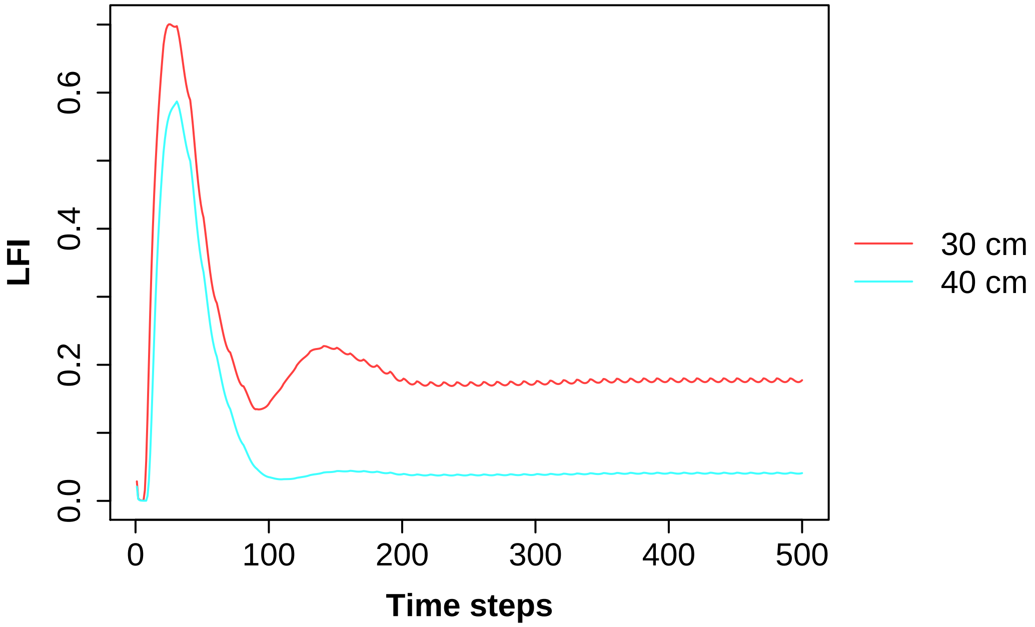

LFI
0.0
0.2
0.4
0.6
0
100
200
300
400
500
Time steps
30 cm
40 cm

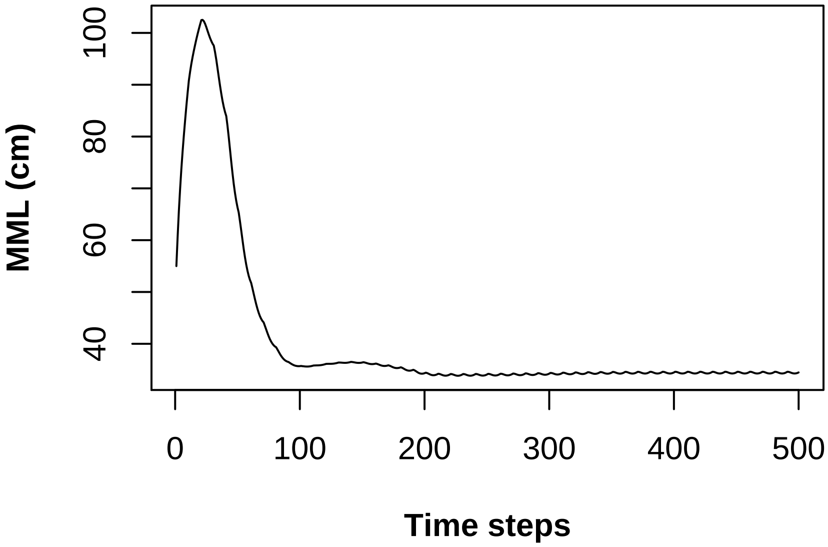

MML (cm)
40
60
80
100
0
100
200
300
400
500
Time steps

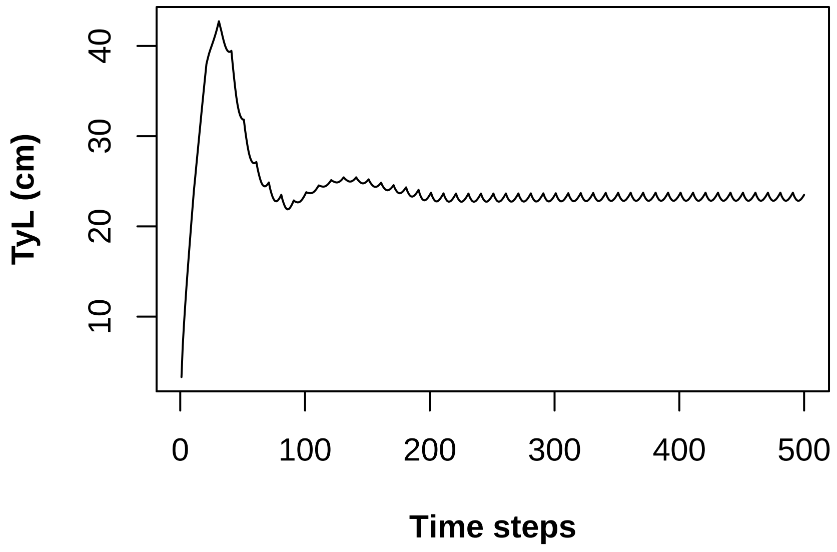

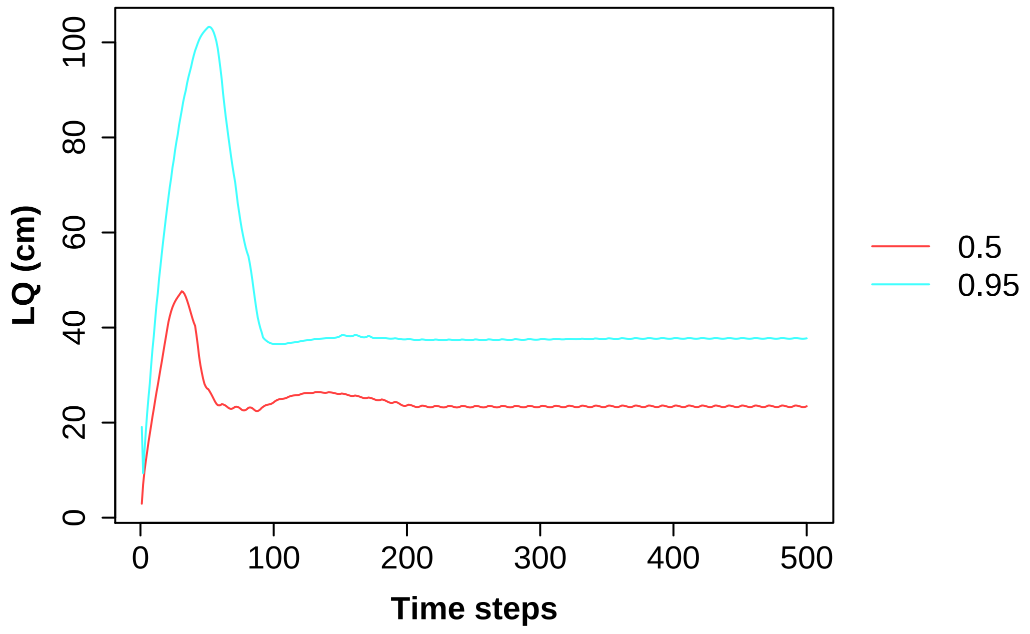

`LeMaRns` also includes the `get_indicators` and `plot_indicators` functions, which can be used to calculate and plot all four indicators using a single function call. For example, the indicators can be calculated by running:

```
indicators <- get_indicators(inputs=NS_params, outputs=model_run, time_steps=1:500,
                             species=1:21,length_LFI=c(30, 40), prob=c(0.5, 0.95))
```

which returns a list object with the names `LFI`, `MML`, `TYL` and `LQ`.

To plot the indicators, users can again provide either `LeMans_param` and `LeMans_output` objects:

```
plot_indicators(inputs=NS_params, outputs=model_run, time_steps=1:500, species=1:21,
                length_LFI=c(30, 40), prob=c(0.5, 0.95))
```

or they can provide the output of `get_indicators` to the plot function directly:

```
plot_indicators(LFI=indicators[['LFI']], MML=indicators[['MML']],
                TyL=indicators[['TyL']], LQ=indicators[['LQ']],
                time_steps=1:500, species=1:21,
                length_LFI=c(30, 40), prob=c(0.5, 0.95))
```

In both cases, the following plot will be returned:

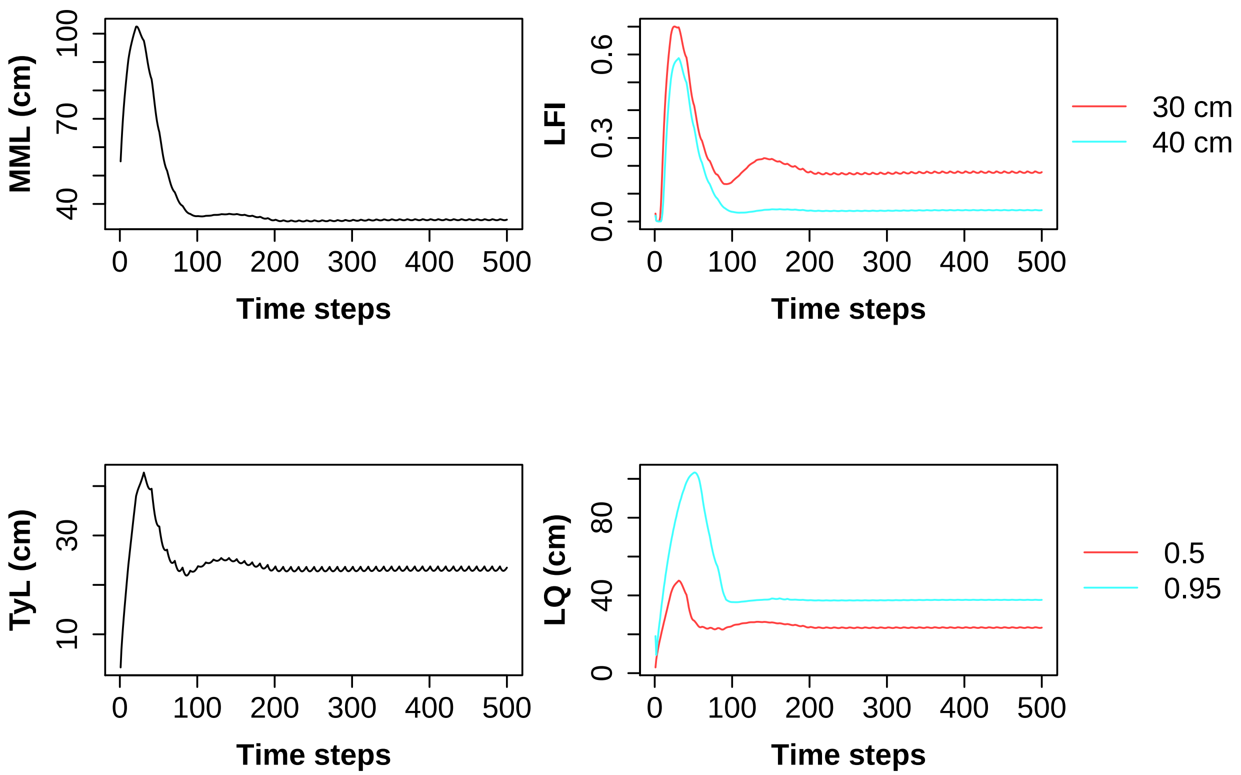

Catch

Finally, `LeMaRns` includes in-built functions that can be used to explore catch. Catch Per Unit Effort (`CPUE`) and Catch Per Gear (`CPG`) can be calculated using the `get_CPUE()` and `get_CPG()` functions in the same way as described previously:

```
CPUE <- get_CPUE(inputs=NS_params, outputs=model_run, time_steps=1:500, species=1:21)
CPG <- get_CPG(inputs=NS_params, outputs=model_run, time_steps=1:500, species=1:21)
```

Case Study

In this section we give two examples of mixed fisheries and an example of finding long-term fishing targets. In the first we examine the effect on the fish species of various fishing efforts on different fleets and in the second demonstrate an example with dynamic fishing mortality. The final example calculates a Nash equilibrium.

Please note that the parameters in the example dataset in `LeMaRns` have not been calibrated to observations. Therefore the case studies are demonstrations of the package and the results should not be interpreted. For methods on calibrating these kinds of models see Thorpe et al. (2015) or Spence, Blackwell, and Blanchard (2016).

Fishing fleets

In this case study we explore the mixed fishery example described in Thorpe et al. (2016), which involves four different fishing fleets. Here, we use idealised fleets, i.e. a single fish is caught by only one gear type. The data frame `NS_mixed_fish` contains the information on which fleet catches which species:

```
NS_mixed_fish
#>     catch_species    curve  gear_name max_catchability
#> 1           Sprat logistic Industrial            1.060
#> 2           Npout logistic Industrial            0.477
#> 3         Sandeel logistic Industrial            0.573
#> 4        Poor cod logistic      Otter            0.548
#> 5  Long rough dab logistic       Beam            0.548
#> 6             Dab logistic       Beam            0.548
#> 7         Herring logistic    Pelagic            0.247
#> 8  Horse mackerel logistic    Pelagic            0.627
#> 9      Lemon sole logistic       Beam            0.548
#> 10           Sole logistic       Beam            0.534
#> 11       Mackerel logistic    Pelagic            0.627
#> 12        Whiting logistic      Otter            0.365
#> 13          Witch logistic       Beam            0.548
#> 14        Gurnard logistic      Otter            0.548
#> 15         Plaice logistic       Beam            0.548
#> 16     Starry ray logistic       Beam            0.548
#> 17        Haddock logistic      Otter            0.577
#> 18     Cuckoo ray logistic       Beam            0.548
#> 19       Monkfish logistic      Otter            0.548
#> 20            Cod logistic      Otter            0.882
#> 21         Saithe logistic      Otter            0.377
```

In this example, the selectivity of each species follows the `logistic` curve and requires the parameters `NS_eta` and `NS_L50`, which are provided as part of `LeMaRns`.

We first set up the `LeMans_params` object:

```
NS_params <- LeMansParam(df=NS_par, gdf = NS_mixed_fish, tau=NS_tau, eta=NS_eta, L50=NS_L50, other=NS_o
#> Warning in .local(df, gdf, ...): The following columns of df do not match any of the species argumen
#> a, b
```

Initially we will examine the virgin biomass by running the model to equilibrium with no fishing. To do this, we set up effort and then run the model for 50 years:

```
effort_mat <- matrix(0, 50, dim(NS_params@Qs)[3])
colnames(effort_mat) <- c("Industrial", "Otter", "Beam", "Pelagic")
model_run <- run_LeMans(NS_params, years=50, effort=effort_mat)
```

We plot Spawning Stock Biomass (SSB) to ensure it has reached equilibrium:

```
plot_SSB(inputs=NS_params, outputs=model_run, full_plot_only=TRUE)
```

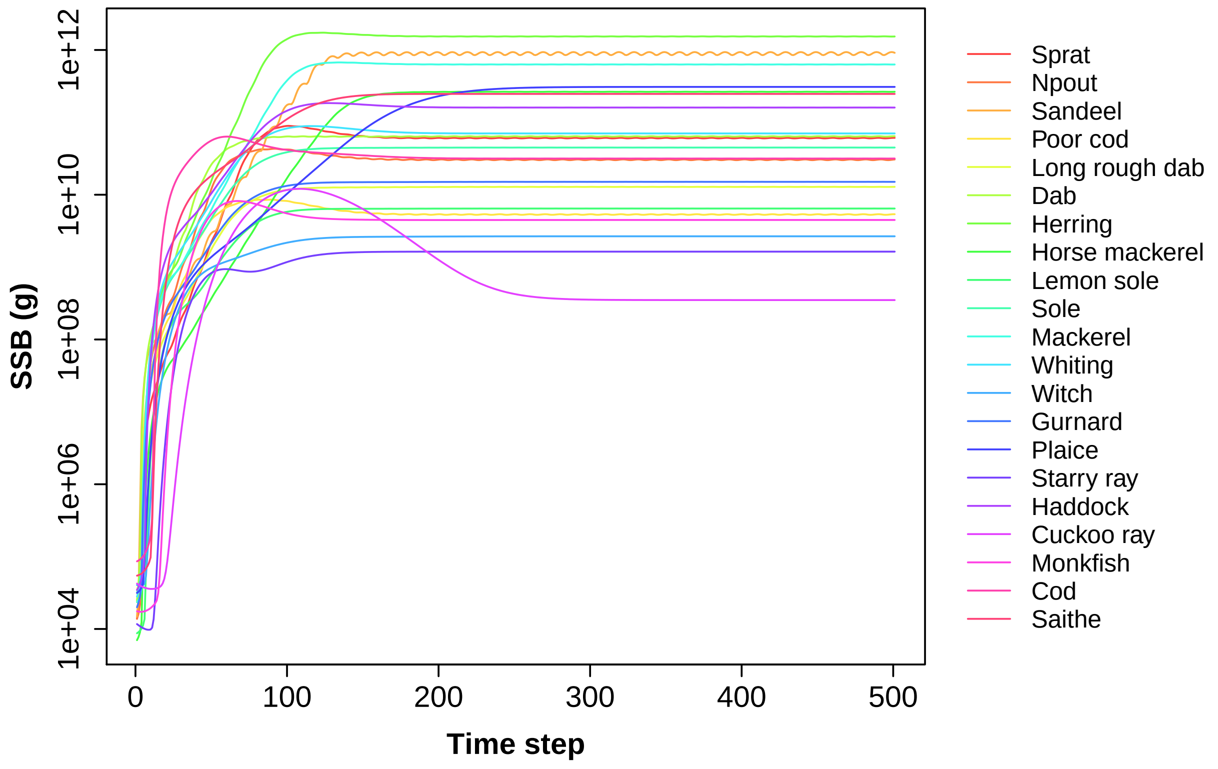

and then check the indicators:

```
plot_indicators(inputs=NS_params, outputs=model_run)
```

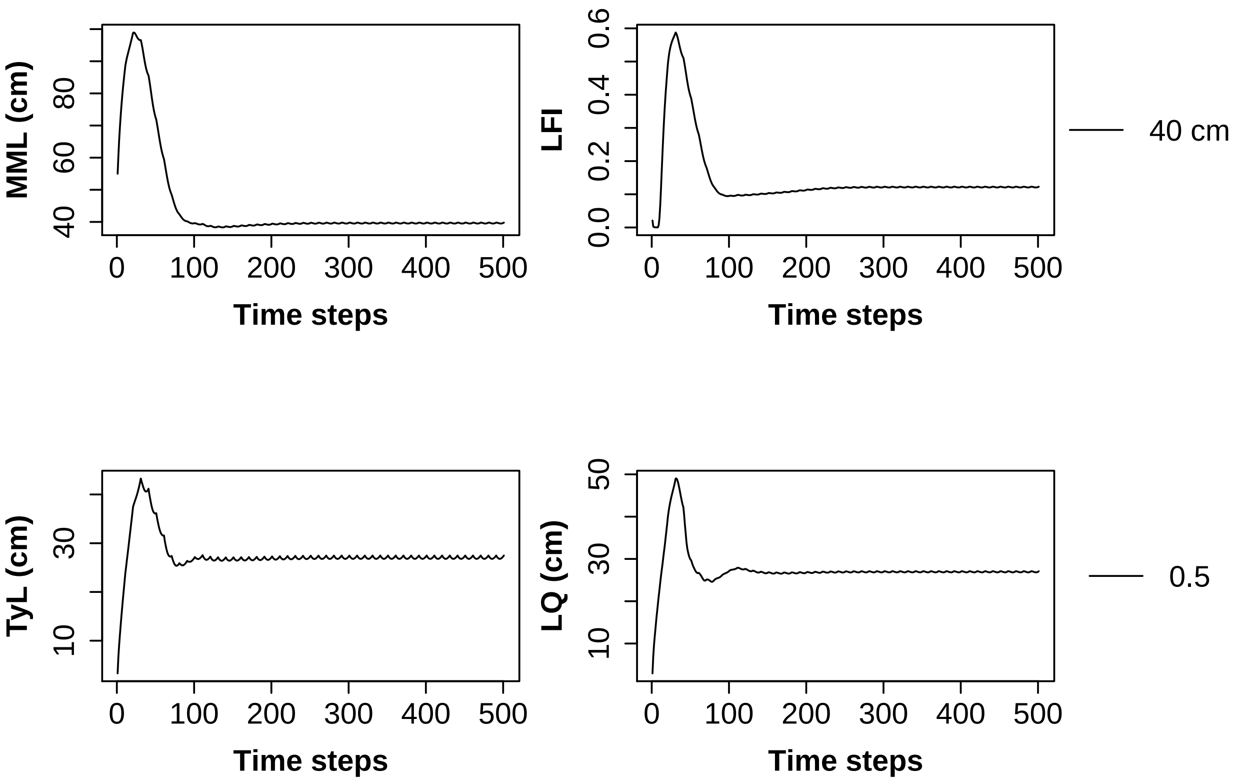

We take the mean of the final year of the simulation to calculate the virgin SSB:

```
v_SSB <- colMeans(get_SSB(inputs=NS_params, outputs=model_run, time_steps=492:501))/1e6
```

In Thorpe et al. (2016) a factorial design was used to explore the effects of changing the fishing effort of different fleets. In this example we allow each of the four fleets to fish at one of five effort levels and set up a factorial design:

```
ef_lvl <- c(0, 0.5, 1, 1.5, 2)
efs <- expand.grid(Industrial=ef_lvl, Otter=ef_lvl, Beam=ef_lvl, Pelagic=ef_lvl)
```

We run the LeMans model for 50 years from the final state of the model in the `model_run` above. For efficiency we create a function that takes in the factorial design and then runs the LeMans model:

```
run_the_model<- function(ef){
  effort_mat <- matrix(ef, 50, dim(NS_params@Qs)[3], byrow=T)
  colnames(effort_mat) <- c("Industrial", "Otter", "Beam", "Pelagic")
  model_run <- run_LeMans(params=NS_params, N0=model_run@N[,,501],
                          years=50, effort=effort_mat)
  return(model_run)
}
```

We then run the model over the factorial design (warning this may take a few minutes):

```
sce <- apply(efs, 1, run_the_model)
```

We then calculate the Large Fish Indicator (LFI), Typical Length (TyL) and Mean Maximum Length (MML) for all of the model runs:

```r
LFI <- unlist(lapply(lapply(sce, FUN=get_LFI, time_steps=492:501, inputs=NS_params),
                     mean))
TyL <- unlist(lapply(lapply(sce, FUN=get_TyL, time_steps=492:501, inputs=NS_params),
                     mean))
MML <- unlist(lapply(lapply(sce, FUN=get_MML, time_steps=492:501, inputs=NS_params),
                     mean))
```

We can now investigate the effect of changing the effort of each of the fishing gears on the above indicators using box-plots:

```r
par(mfrow=c(2, 2))
boxplot(LFI~efs[, 1], main="Industrial", xlab="Effort", ylab="LFI")
boxplot(LFI~efs[, 2], main="Otter", xlab="Effort", ylab="LFI")
boxplot(LFI~efs[, 3], main="Beam", xlab="Effort", ylab="LFI")
boxplot(LFI~efs[, 4], main="Pelagic", xlab="Effort", ylab="LFI")
```

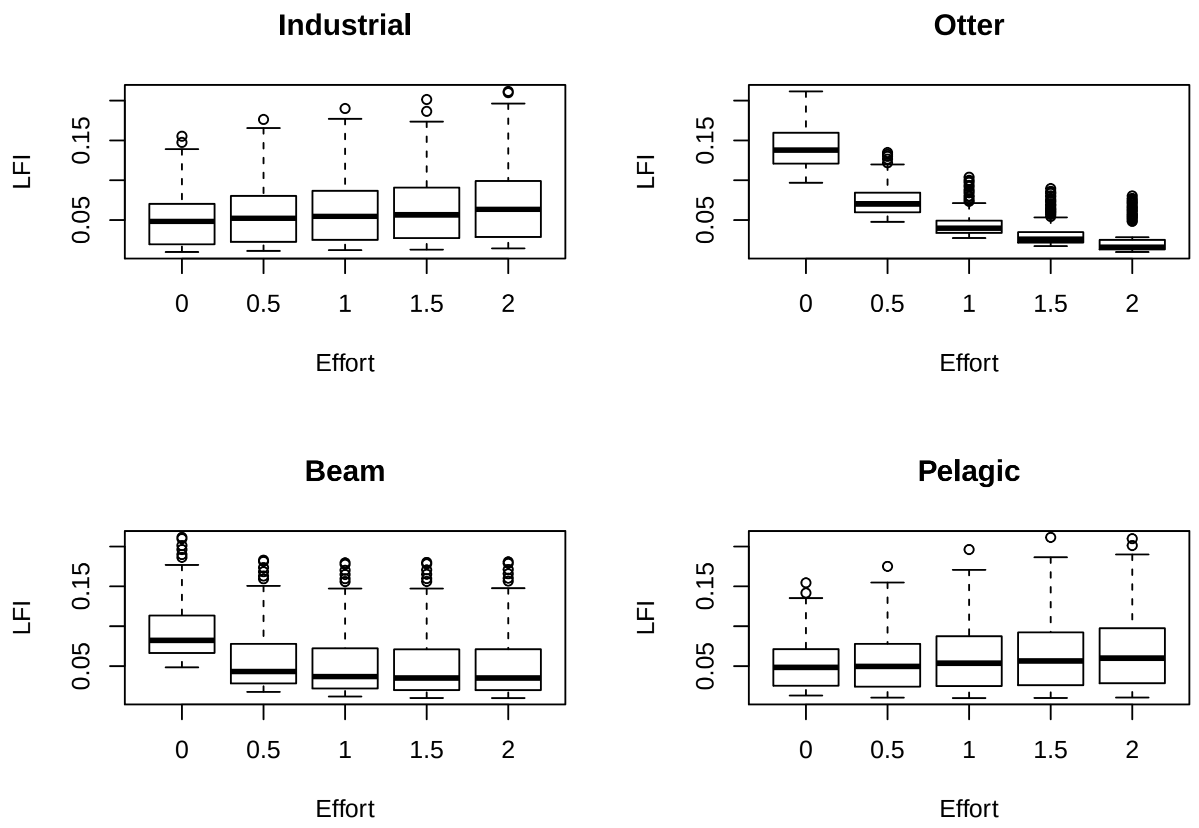


```r
par(mfrow=c(2, 2))
boxplot(TyL~efs[, 1], main="Industrial", xlab="Effort", ylab="TyL")
boxplot(TyL~efs[, 2], main="Otter", xlab="Effort", ylab="TyL")
boxplot(TyL~efs[, 3], main="Beam", xlab="Effort", ylab="TyL")
boxplot(TyL~efs[, 4], main="Pelagic", xlab="Effort", ylab="TyL")
```

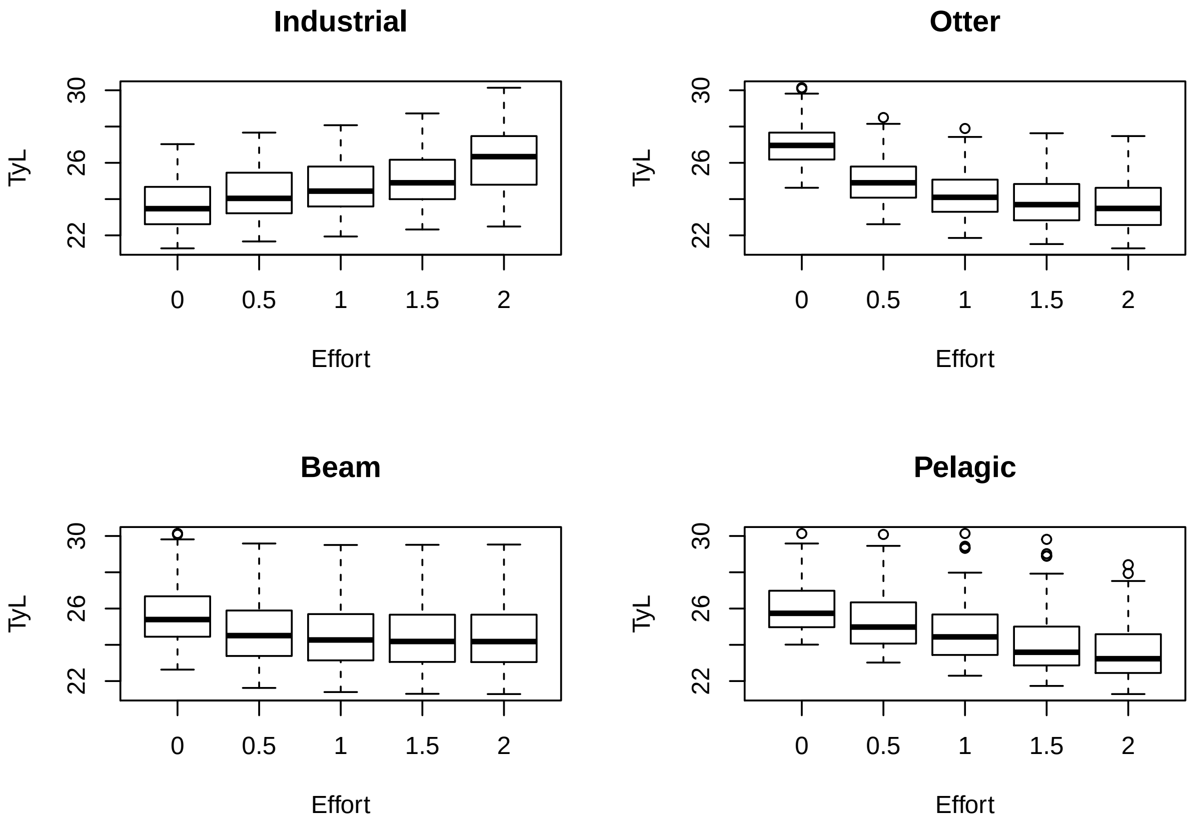


```r
par(mfrow=c(2, 2))
boxplot(MML~efs[, 1], main="Industrial", xlab="Effort", ylab="MML")
boxplot(MML~efs[, 2], main="Otter", xlab="Effort", ylab="MML")
boxplot(MML~efs[, 3], main="Beam", xlab="Effort", ylab="MML")
boxplot(MML~efs[, 4], main="Pelagic", xlab="Effort", ylab="MML")
```

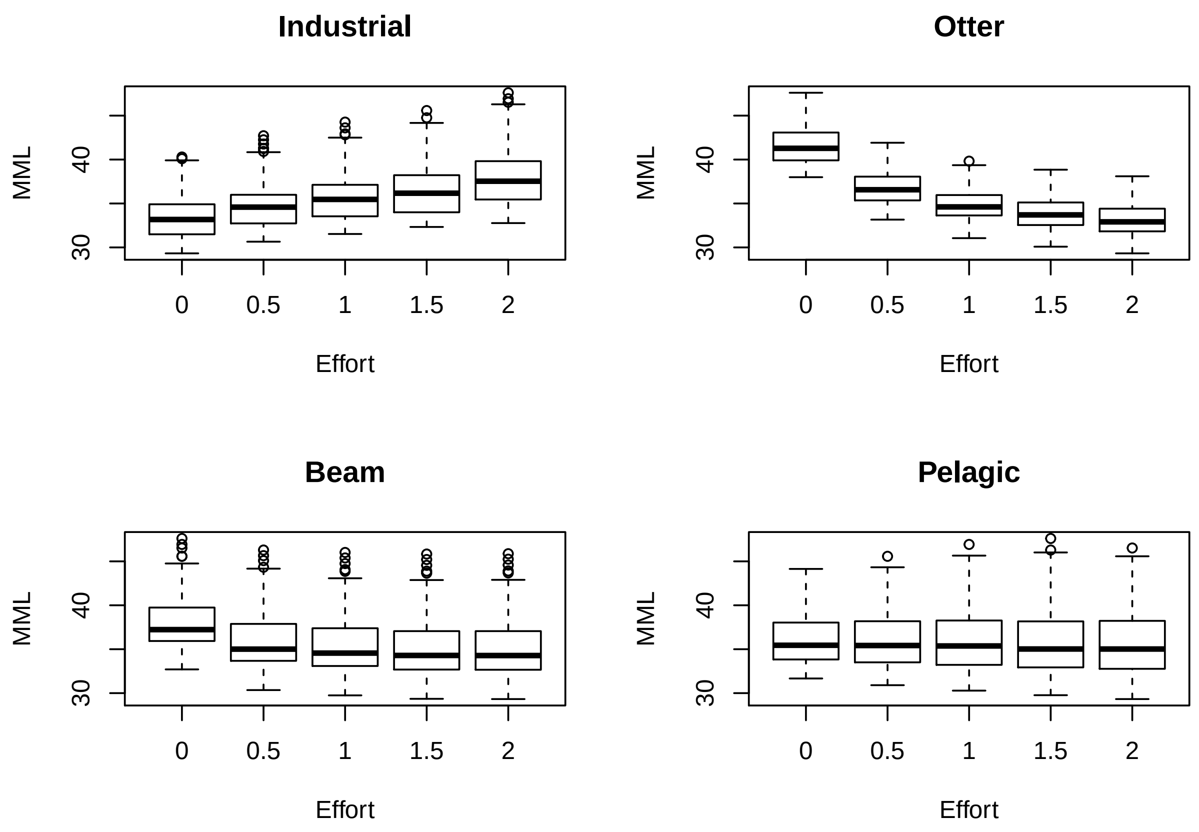

All three indicators appear to be mostly driven by the otter gear, which targets larger fish species such as cod and saithe. This suggests that the three indicators are predominantly metrics of the health of the larger species rather than the smaller species.

We also investigate the state of the stocks relative to the virgin SSB:

```
# SSB of the final years (tonnes)
new_SSB <- do.call(rbind, lapply(lapply(sce, FUN=get_SSB, time_steps=492:501,
                                        inputs=NS_params), colMeans))/1e6
# Relative SSB
rel_SSB <- t(t(new_SSB)/v_SSB)
colnames(rel_SSB) <- NS_params@species_names
```

Changes in the relative SSB of saithe based on fishing effort is shown below:

```
par(mfrow=c(2,2))
boxplot(rel_SSB[, "Saithe"]~efs[, 1], main="Industrial", xlab="Effort",
        ylab="Relative SSB")
boxplot(rel_SSB[, "Saithe"]~efs[, 2], main="Otter", xlab="Effort",
        ylab="Relative SSB")
boxplot(rel_SSB[, "Saithe"]~efs[, 3], main="Beam", xlab="Effort",
        ylab="Relative SSB")
boxplot(rel_SSB[, "Saithe"]~efs[, 4], main="Pelagic", xlab="Effort",
        ylab="Relative SSB")
```

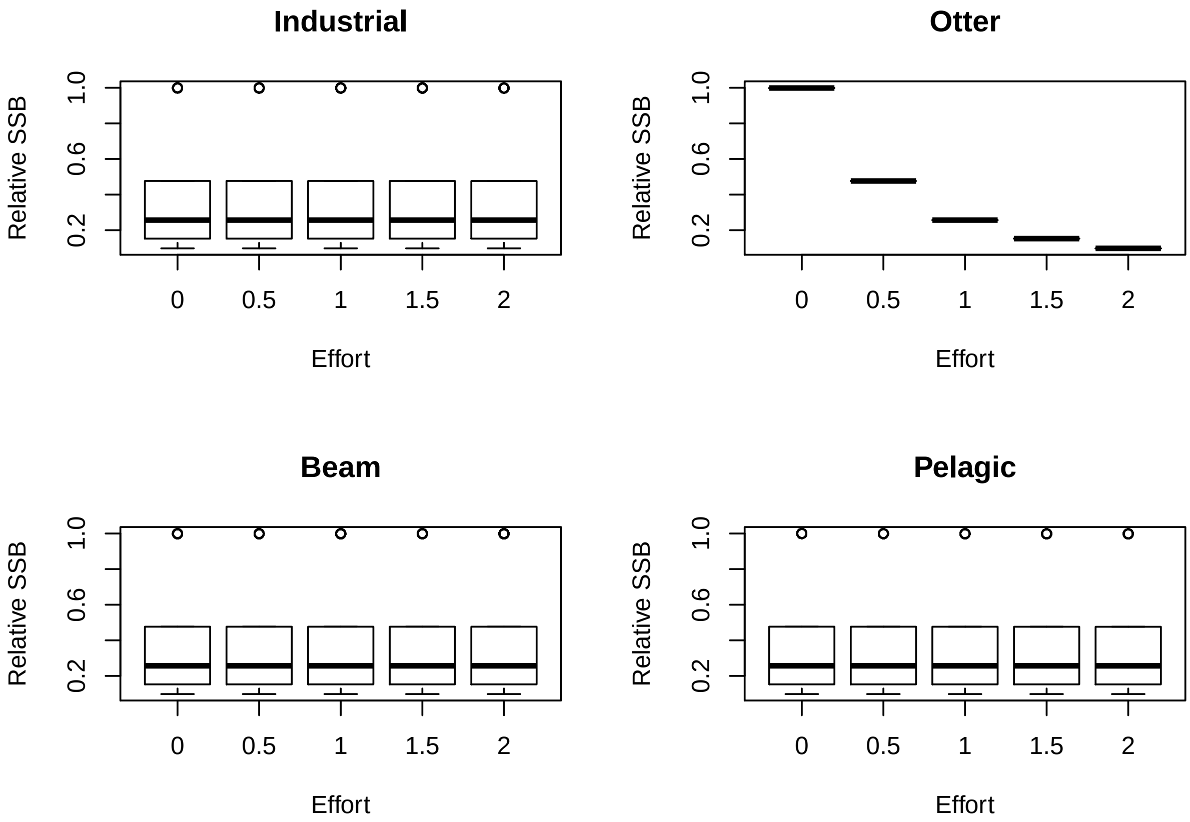

As expected, the SSB of saithe is most sensitive to the otter gear, which is the fleet that catches it. We now examine the effects of changes in fishing effort on horse mackerel:

```
par(mfrow=c(2,2))
boxplot(rel_SSB[, "Horse mackerel"]~efs[, 1], main="Industrial", xlab="Effort",
        ylab="Relative SSB")
boxplot(rel_SSB[, "Horse mackerel"]~efs[, 2], main="Otter", xlab="Effort",
        ylab="Relative SSB")
boxplot(rel_SSB[, "Horse mackerel"]~efs[, 3], main="Beam", xlab="Effort",
        ylab="Relative SSB")
boxplot(rel_SSB[, "Horse mackerel"]~efs[, 4], main="Pelagic", xlab="Effort",
        ylab="Relative SSB")
```

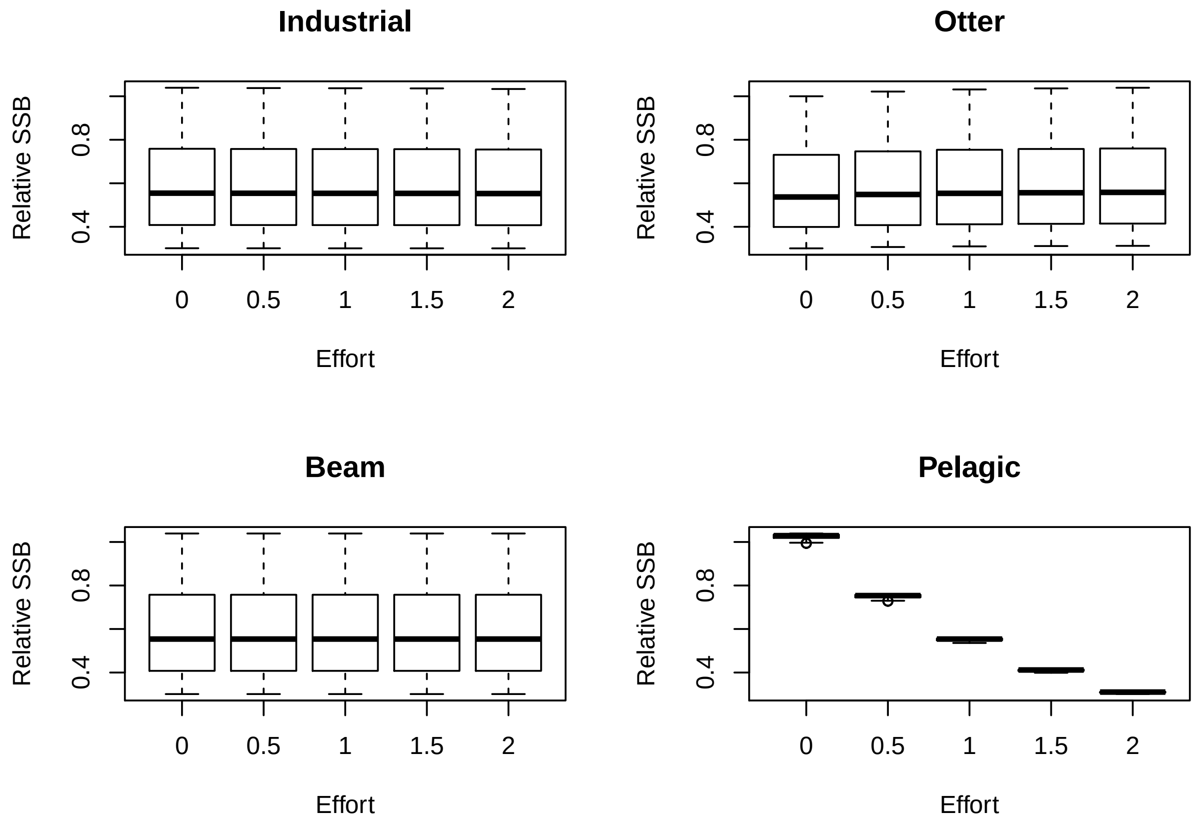

The SSB of horse mackerel is most sensitive to the pelagic fleet, which is the only fleet that catches this species. However, horse mackerel SSB is also sensitive to the otter fleet. It is also interesting to note that under zero pelagic fishing, the SSB of horse mackerel can exceed that of the virgin biomass depending on the fishing effort of the other fleets. This is because of predator-prey interactions and the changing dynamics of the other species, particularly a decrease in the predators of horse mackerel.

In Thorpe et al. (2016), a stock is said to be at risk of collapse if the SSB falls below 10% of the virgin SSB. Below we show the number of stocks that are at risk of collapse for each of the fishing scenarios:

```
risk <- apply(rel_SSB, 1, function(x){return(sum(x<0.1))})

par(mfrow=c(2, 2))
boxplot(risk~efs[, 1], main="Industrial", xlab="Effort", ylab="Stocks at risk")
boxplot(risk~efs[, 2], main="Otter", xlab="Effort", ylab="Stocks at risk")
boxplot(risk~efs[, 3], main="Beam", xlab="Effort", ylab="Stocks at risk")
boxplot(risk~efs[, 4], main="Pelagic", xlab="Effort", ylab="Stocks at risk")
```

The number of stocks at risk of collapse is sensitive to the otter fleet and the beam fleet. Below we plot the mean number of stocks at risk by varying the effort of the otter and the beam fleets:

```
z_mat <- outer(ef_lvl, ef_lvl, FUN=function(x, y, efs) {
  mapply(function(x, y, efs) {
  mean(risk[intersect(which(efs[, 2]==x), which(efs[, 3]==y))])}, x=x, y=y,
  MoreArgs=list(efs=efs))
}, efs=efs)
layout(matrix(c(1,1,1,1,1,2), nrow=1, byrow=TRUE))
image(z=-z_mat, x=ef_lvl, y=ef_lvl, axes=T, cex.lab=1.5, xlab="Otter effort", ylab="Beam effort")
```

```
axis(1); axis(2); box()
image(z=-matrix(sort(unique(as.numeric(z_mat))), nrow=1),
      y=sort(unique(as.numeric(z_mat))), axes=F, cex.lab=1.5,
      ylab="Number of stocks at risk")
axis(2); box()
box()
```

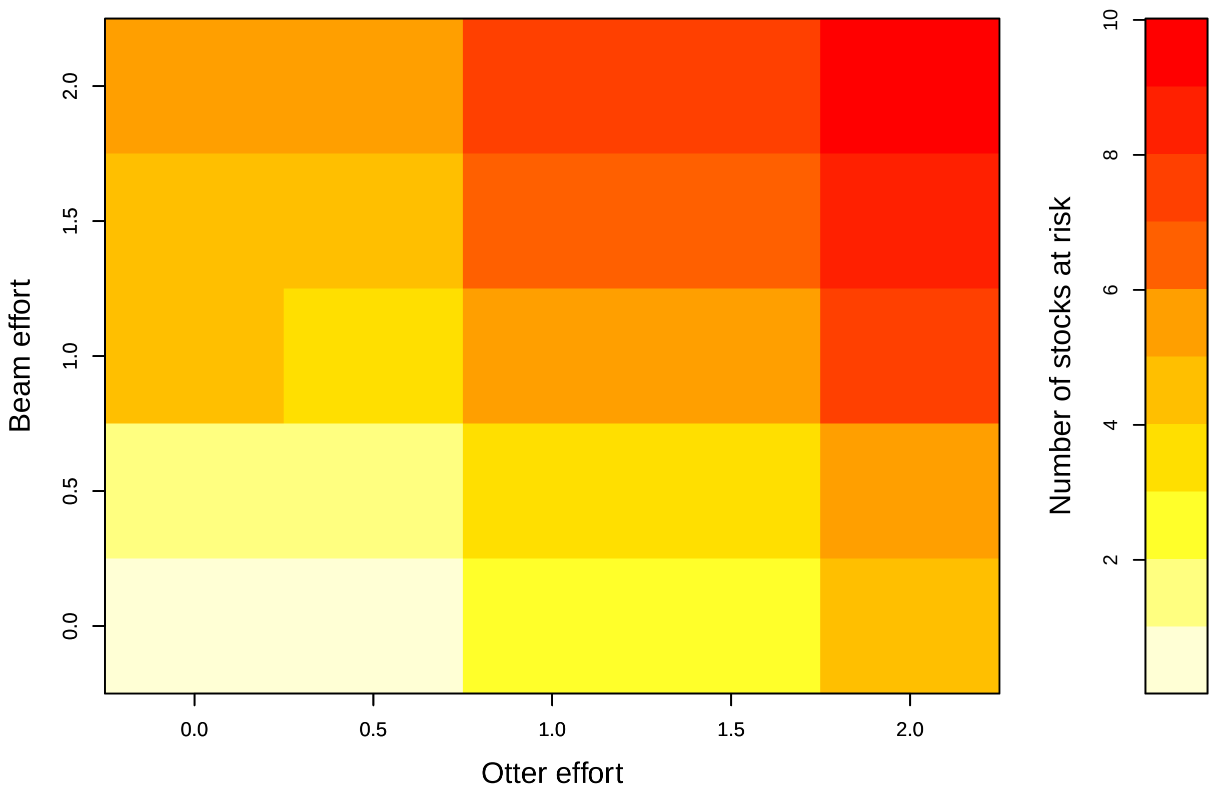

Dynamic fishing

We now investigate a dynamical fishing scenario. We first set up effort for the four gears with varying fishing effort, shown below:

```
Industrial <- rep(1.5, 20)
Otter <- -1/100*1:20*(1:20-20)
Beam <- 1:20*1/20+0.25
Pelagic <- 1+c(1:5*1/5, 5:1*1/5, 1:5*1/5, 5:1*1/5)
par(mfrow=c(2, 2))
plot(1:20, Industrial, ylab="Effort", main="Industrial", xlab="Year",
     ylim=c(0, 2), type="s")
plot(1:20, Otter, ylab="Effort", main="Otter", xlab="Year",
     ylim=c(0, 2), type="s")
plot(1:20, Beam, ylab="Effort", main="Beam", xlab="Year",
     ylim=c(0, 2), type="s")
plot(1:20, Pelagic, ylab="Effort", main="Pelagic", xlab="Year",
```

```
     ylim=c(0, 2), type="s")
```

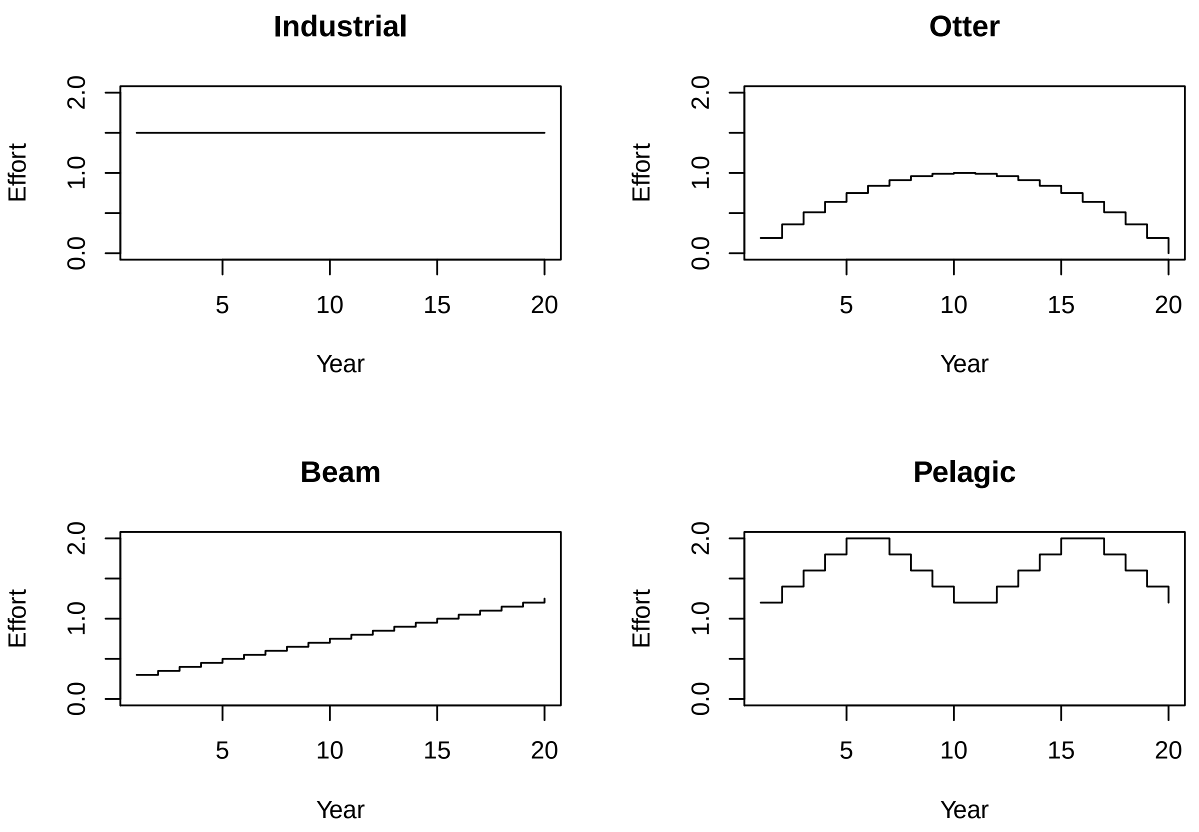

We then run the model for 20 years from the unfished state (`model_run`) and calculate the annual catches for those 20 years:

```
# Set up effort for the model run
effort_mat <- cbind(Industrial, Otter, Beam, Pelagic)
colnames(effort_mat) <- c("Industrial", "Otter", "Beam", "Pelagic")
model_run_dyn <- run_LeMans(params=NS_params, N0=model_run@N[,,501],
                            years=20, effort=effort_mat)
catches <- get_annual_catch(inputs=NS_params, outputs=model_run_dyn)/1e6 # in tonnes
colnames(catches) <- NS_params@species_names
```

We can then plot the annual catches for different species:

```
par(mfrow=c(2, 2))
plot(1:20, catches[, "Sprat"], type="l", main="Sprat", xlab="Year",
     ylab="Catch (tonnes)", ylim=c(0, max(catches[, "Sprat"])))
plot(1:20, catches[, "Cod"], type="l", main="Cod", xlab="Year",
     ylab="Catch (tonnes)", ylim=c(0, max(catches[, "Cod"])))
plot(1:20, catches[, "Sole"], type="l", main="Sole", xlab="Year",
     ylab="Catch (tonnes)", ylim=c(0,max(catches[, "Sole"])))
plot(1:20, catches[, "Herring"], type="l", main="Herring", xlab="Year",
     ylab="Catch (tonnes)", ylim=c(0, max(catches[, "Herring"])))
```

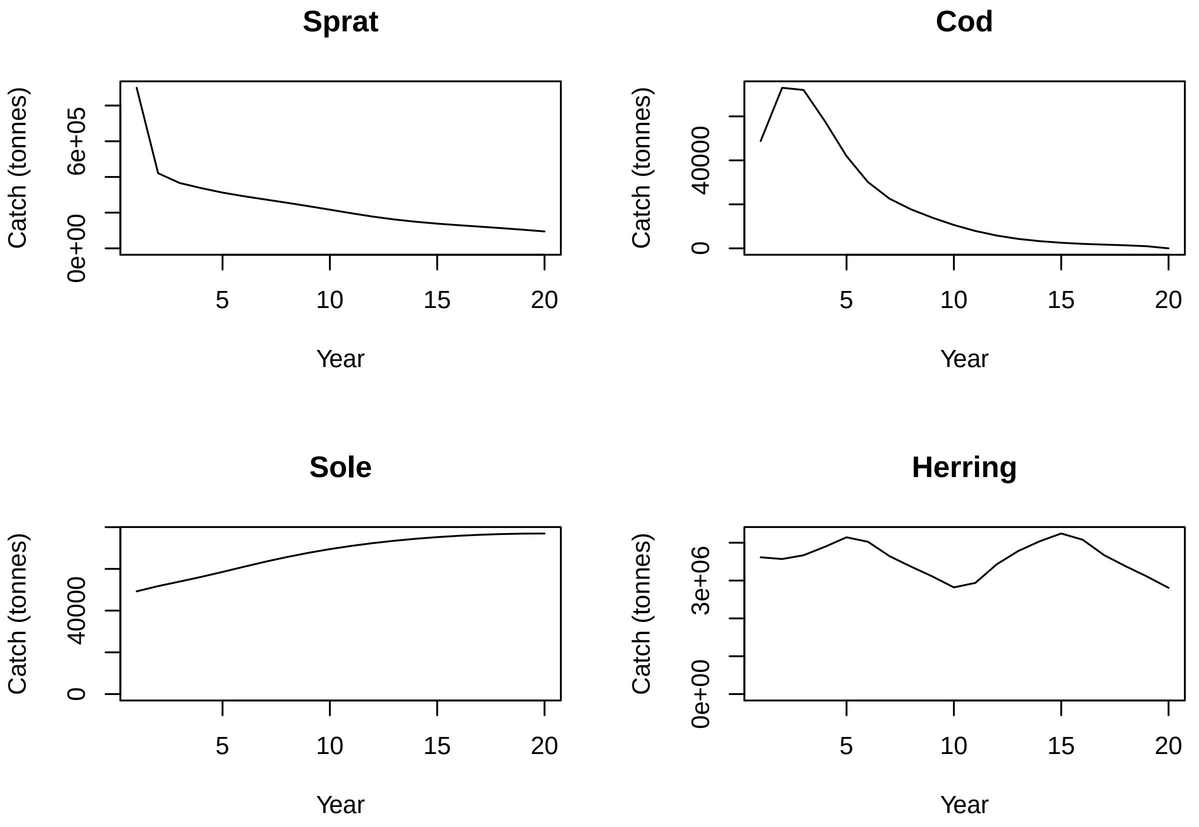

We can see that sprat and cod is very quickly overfished. We know this because increases in effort beyond a certain point result in a decrease in the total catch of cod. This occurs as the stock size continually decreases for much of the model run, which we can see below. The catch of sole increases as effort increases and the catch of herring also follows the effort dynamics. Below we plot the SSB (in grams):

```
plot_SSB(inputs=NS_params, outputs=model_run_dyn,
         species=c("Sprat", "Cod", "Sole", "Herring"),
         full_plot_only=FALSE)
```

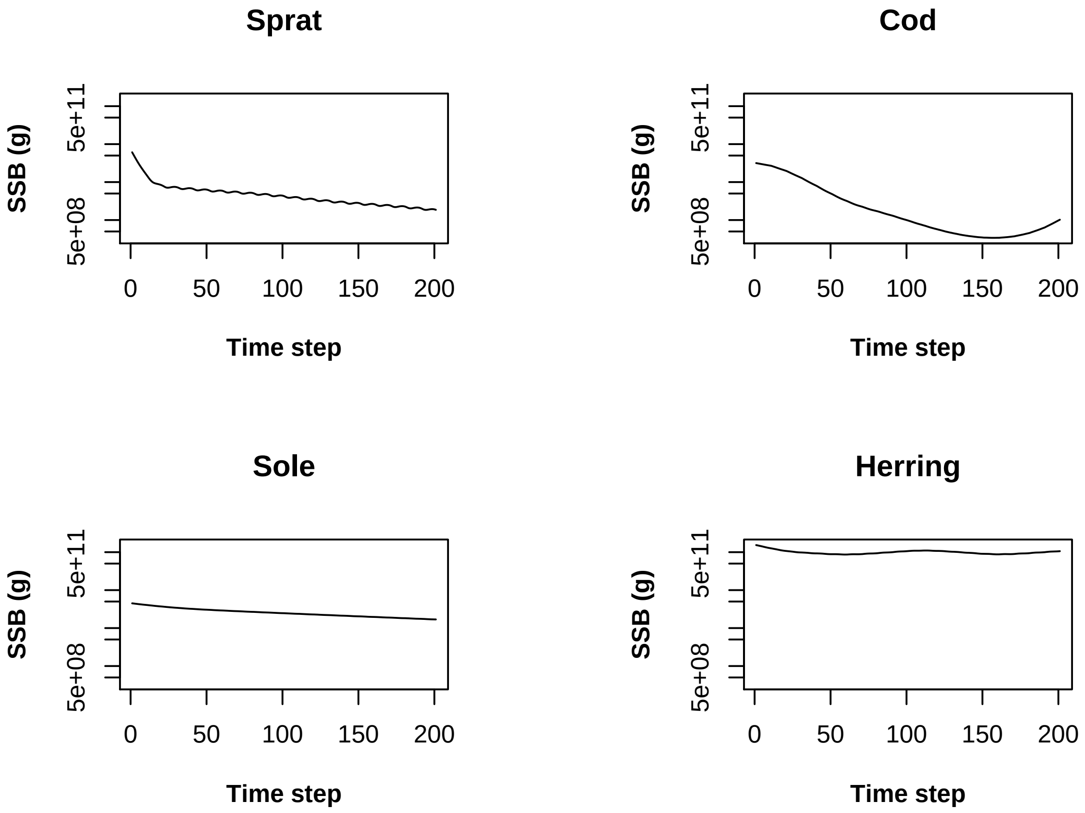
Sprat
SSB (g)
5e+08
5e+11
0
50
100
150
200
Time step
Cod
SSB (g)
5e+08
5e+11
0
50
100
150
200
Time step
Sole
SSB (g)
5e+08
5e+11
0
50
100
150
200
Time step
Herring
SSB (g)
5e+08
5e+11
0
50
100
150
200
Time step

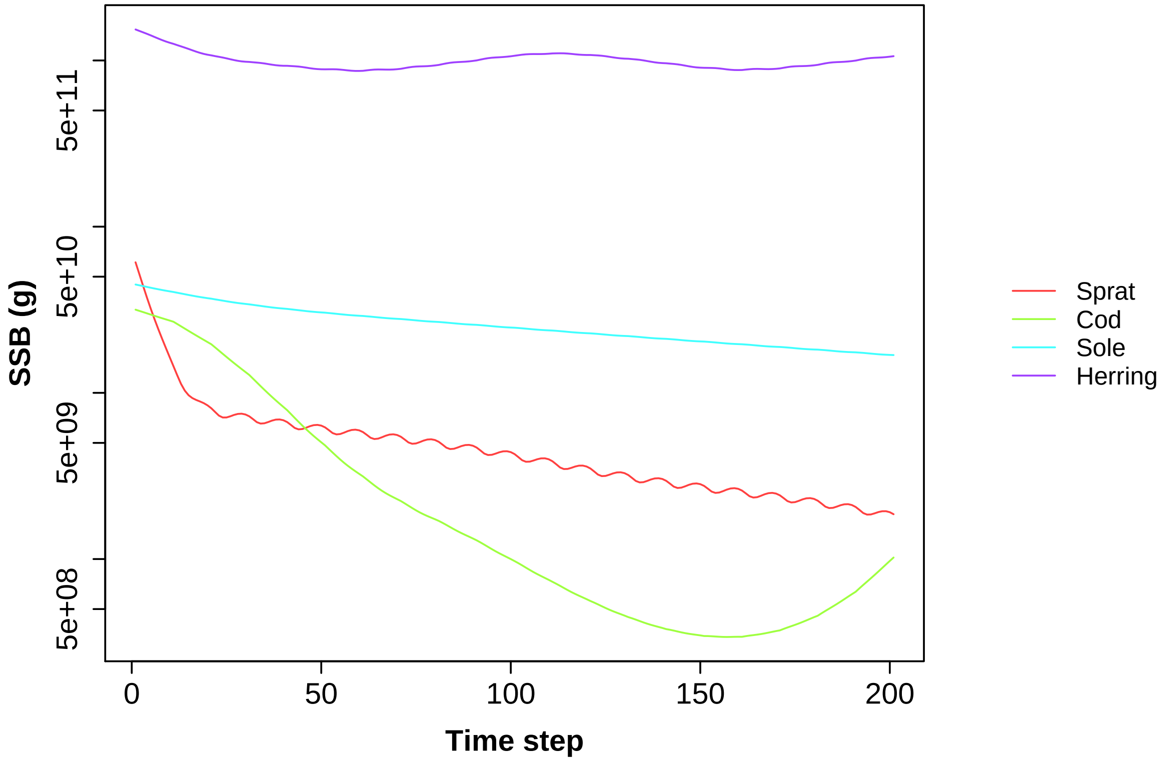

We also calculate the total catch for each gear and plot it for each year:

```
catch_per_gear <- get_CPG(inputs=NS_params, outputs=model_run_dyn, effort=effort_mat)
tot_pg <- apply(catch_per_gear, c(2, 3), sum)/1e6
year <- rep(1:20, each=10)
# Total catch per gear per year
tot_pgpy <- t(sapply(1:20, function(x, year){
  tele <- which(year==x)
  if (length(tele)>1){
    return(rowSums(tot_pg[, tele]))
  }
  return(tot_pg[, tele])
}, year=year))

par(mfrow=c(2, 2))
plot(1:20, tot_pgpy[, 1], type="l", ylim=c(0, max(tot_pgpy[, 1])), xlab="Year",
     main="Industrial", ylab="Catch (tonnes)")
plot(1:20, tot_pgpy[, 2], type="l", ylim=c(0, max(tot_pgpy[, 2])), xlab="Year",
     main="Otter", ylab="Catch (tonnes)")
plot(1:20, tot_pgpy[, 3], type="l", ylim=c(0, max(tot_pgpy[, 3])), xlab="Year",
     main="Beam", ylab="Catch (tonnes)")
plot(1:20, tot_pgpy[, 4], type="l", ylim=c(0, max(tot_pgpy[, 4])), xlab="Year",
     main="Pelagic", ylab="Catch (tonnes)")
```

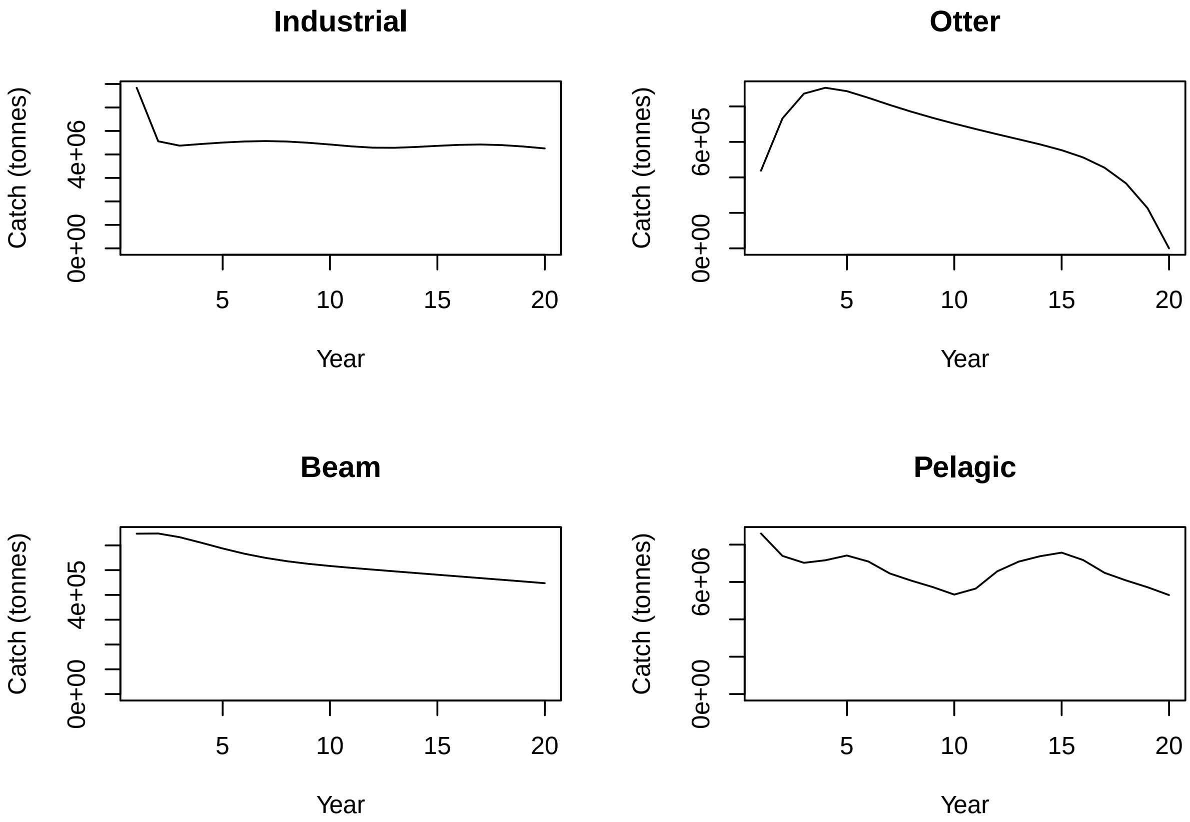

We can also investigate the Catch Per Unit Effort (CPUE) for each species. We calculate the mean CPUE across each year:

```
CPUE <- get_CPUE(inputs=NS_params, outputs=model_run_dyn, effort=effort_mat)/1e6
cpue_py <- t(sapply(1:20, function(x, year){
  tele <- which(year==x)
  if (length(tele)>1){
    return(colMeans(CPUE[tele, ]))
  }
  return(CPUE[tele, ])
}, year=year))

colnames(cpue_py) <- NS_params@species_names

par(mfrow=c(2, 2))
plot(1:20, cpue_py[, "Sprat"], type="l", main="Sprat", xlab="Year",
     ylab="CPUE (tonnes)", ylim=c(0, max(cpue_py[, "Sprat"])))
plot(1:20, cpue_py[, "Cod"], type="l", main="Cod", xlab="Year",
     ylab="CPUE (tonnes)", ylim=c(0, max(cpue_py[, "Cod"])))
plot(1:20, cpue_py[, "Sole"], type="l", main="Sole", xlab="Year",
     ylab="CPUE (tonnes)", ylim=c(0, max(cpue_py[, "Sole"])))
plot(1:20, cpue_py[, "Herring"], type="l", main="Herring", xlab="Year",
     ylab="CPUE (tonnes)", ylim=c(0, max(cpue_py[, "Herring"])))
```

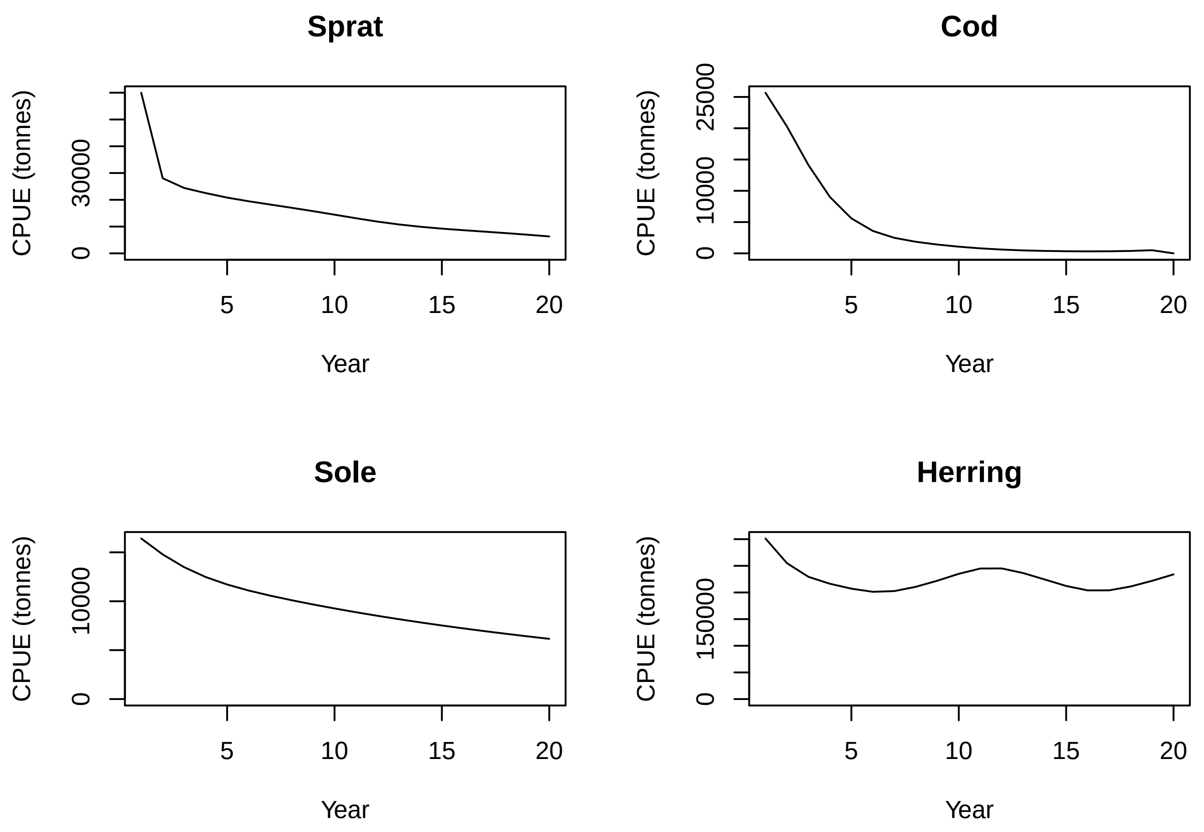

Sprat, Cod and sole are always overfished and herring is overfished in some years and underfished in others.

In `LeMaRns` it is possible for a species to be caught by multiple gears. To demonstrate this we add another fleet - a `Recreational` fleet. The selectivity for this fleet is `knife-edge` and it catches cod, haddock, herring, horse mackerel, mackerel, plaice, saithe and whiting, with all fish exceeding the minimum landing size `Lmin` being retained:

```
rec_fish <- data.frame(catch_species=c("Cod", "Haddock", "Herring", "Horse mackerel",
                                       "Mackerel", "Plaice", "Saithe", "Whiting"),
                       curve=rep("knife-edge"), gear_name="Recreational")
rec_fish$max_catchability <- c(0.01, 0.01, 0.005, 0.05, 0.05, 0.01, 0.01, 0.02)
Lmin <- c(35, 30, 20, 15, 30, 27, 35, 27)
```

To run the `LeMansParam` function `rec_fish` and `NS_mixed_fish` need to be combined:

```
gdf <- rbind(NS_mixed_fish, rec_fish)
```

and `L50`, `eta` and `Lmin` must be the same size as `nrow(gdf)`:

```
eta1 <- c(NS_eta, rep(0, 8))
L501 <- c(NS_L50, rep(0, 8))
Lmin1 <- c(rep(0, 21), Lmin)
```

We can then create the `LeMans_param` object and effort:

```
NS_params_rec <- LeMansParam(df=NS_par, gdf = gdf, tau=NS_tau, eta=eta1, L50=L501,
                             other=NS_other, Lmin=Lmin1)
#> Warning in .local(df, gdf, ...): The following columns of df do not match any of the species argumen
```

```
#> a, b
effort_mat1 <- cbind(effort_mat, 0.1+1:20*0.05/20)
colnames(effort_mat1)[5] <- "Recreational"
```

with the effort of the recreational fishing fleet being:

```
plot(1:20, effort_mat1[, "Recreational"], xlab="Years", ylab="Effort", type="l",
     ylim=c(0, 2), main="Recreational")
```

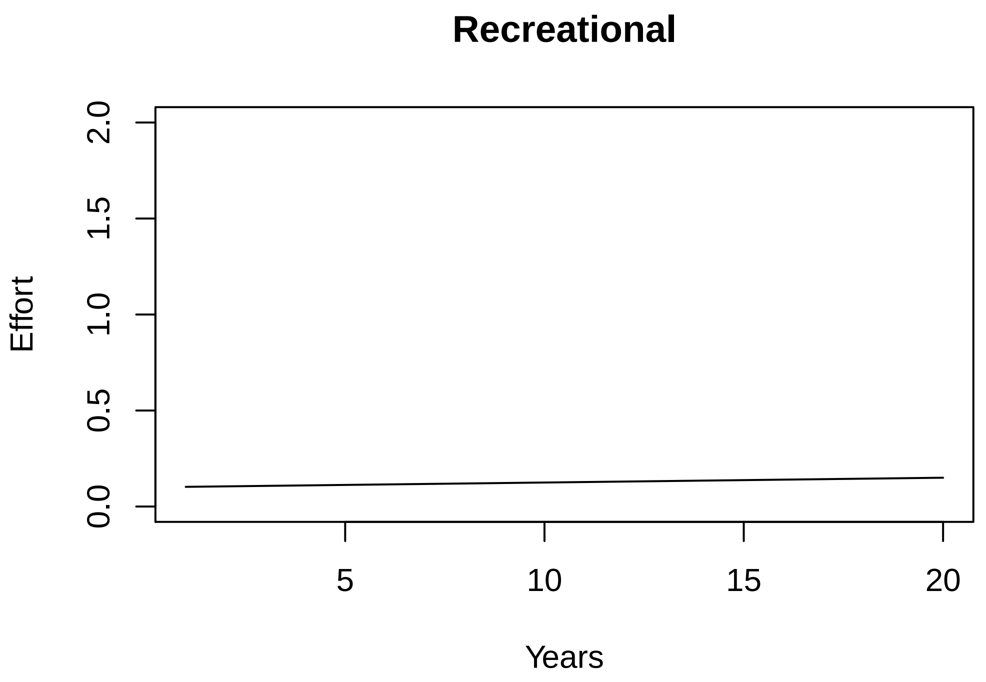

We then run the model:

```
model_run_rec <- run_LeMans(params=NS_params_rec, N0=model_run@N[,,501],
                            years=20, effort=effort_mat1)
```

and explore the catches for the recreational fleet:

```
catch_per_gear_rec <- get_CPG(inputs=NS_params_rec, outputs=model_run_rec, effort=effort_mat1)
rec_py <- t(sapply(1:20, function(x, year){
  tele <- which(year==x)
  if (length(tele)>1){
    return(rowSums(catch_per_gear_rec[, 5, tele]))
  }
  return(catch_per_gear_rec[, 5, tele])
}, year=year))/1e6
colnames(rec_py) <- NS_params_rec@species_names

par(mfrow=c(2, 2))
plot(1:20, rec_py[, "Herring"], type="l", main="Herring", xlab="Year",
     ylab="Catch (tonnes)")
plot(1:20, rec_py[, "Mackerel"], type="l", main="Horse mackerel", xlab="Year",
     ylab="Catch (tonnes)")
```

```r
plot(1:20, rec_py[, "Cod"], type="l", main="Cod", xlab="Year",
     ylab="Catch (tonnes)")
plot(1:20 ,rec_py[, "Saithe"], type="l", main="Saithe", xlab="Year",
     ylab="Catch (tonnes)")
```

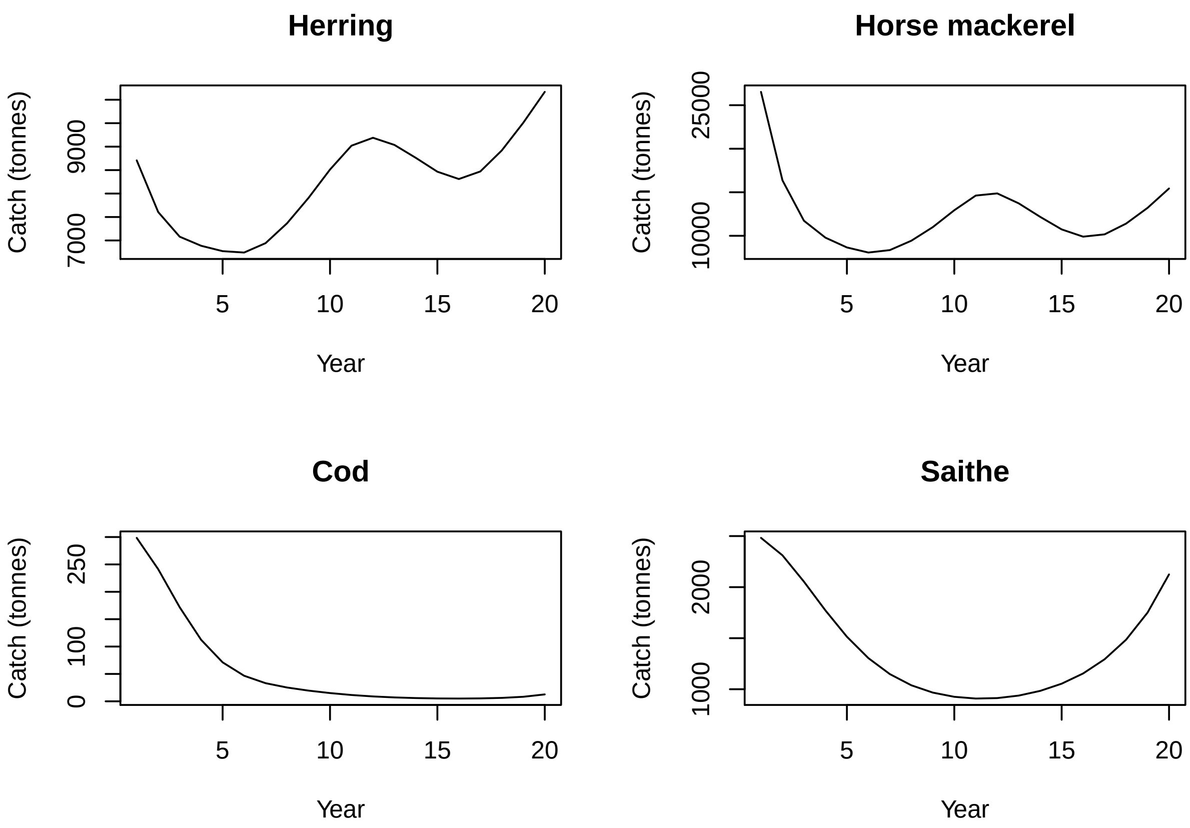

Nash equilibrium

Fish stocks are often managed by considering the fishing mortality that maximises the long-term yield, i.e. the Maximum Sustainable Yield (MSY). We can define $f_i(F_i, F_{-i})$ as the long-term catch of the ith stock, where F_i is the fishing mortality of the ith stock and F_{-i} is the fishing mortalities of the other stocks. Many stocks are managed on a stock by stock basis using single-species models. This means that

$$f_i(F_i, F_{-i}) = f_i(F_i, F'_{-i}),$$

$\forall F'_{-i}$ and we can define

$$F_{MSY,i} = \arg\max_{F_i} f_i(F_i, F'_{-i}).$$

In the single-species world this is commonly well defined. However, in the multispecies world stocks interact with one another and the fishing mortality of the jth stock affects the catch of the ith stock, i.e.

$$f_i(F_i, F_{-i}) \neq f_i(F_i).$$

We therefore need to define a multispecies maximum sustainable yield. One possibility is the Nash equilibrium Thorpe et al. (2016), which is defined as the point at which we are unable to increase $f_i(F_i, F_{-i})$ by changing F_i only, for all i. Formally, $F_{Nash,i}, \forall i$ is a Nash equilibrium when

$$\forall i, F_i : f_i(F_{Nash,i}, F_{Nash,-i}) \geq f_i(F_i, F_{Nash,-i}).$$

We use the `LeMaRns` package to calculate the Nash equilibrium. First we initialise a model run:

```
NS_params_n <- LeMansParam(NS_par, tau=NS_tau, eta=NS_eta, L50=NS_L50, other=NS_other)
#> Warning in .local(df, gdf, ...): The following columns of df do not match any of the species argumen
#> a, b
model_run_n_init <- run_LeMans(NS_params_n, years=50)
# Set the initial state
N0 <- model_run_n_init@N[,,501]
```

We then create a function that returns the negative mean catch of the `i`th species in the 20th year with fishing effort `x` for the `i`th species and `eff` for the rest. As we have set `apply(NS_params_n@Qs,3,max)` to be 1 using `max_catchability`, the maximum fishing mortality of each species is `eff`.

```
calc_catch<-function(x,i,eff){
  eff[i] <- x
  tmp <- run_LeMans(NS_params_n, N0=N0, years=20, effort=matrix(eff, nrow=20, ncol=21, byrow=T))
  return(mean(tail(colSums(tmp@Catch[,i,]), 10)))
}
```

Starting from F_{MSY} in Thorpe et al. (2016), we iteratively optimise the fishing effort for each species. We continue this process until the difference in the optimal effort and the effort at the start of the optimisation differ by less than `0.01` for all species.

```
eff <- fmsy <-c(1.3, 0.35, 0.35, 0.72, 0.6, 0.41, 0.25, 0.5, 0.33, 0.22, 0.32, 0.21, 0.27,
                0.27, 0.25, 0.15, 0.30, 0.11, 0.1, 0.19, 0.3)
stat <- rep(FALSE, 21)
while(any(stat==FALSE)){
  for (i in 1:21){
    opts <- optim(eff[i], calc_catch, i=i, eff=eff, method="Brent", lower=0, upper=2,control = list(fns
    stat[i] <- abs(eff[i]-opts$par)<0.01
    eff[i] <- opts$par
  }
}
```

`eff` is the Nash equilibrium. Below we plot the SSB for the Nash equilibrium:

```
nash <- run_LeMans(NS_params_n, N0=N0, years=20, effort=matrix(eff, nrow=20, ncol=21, byrow=T))
plot_SSB(inputs=NS_params_n, outputs=nash)
```

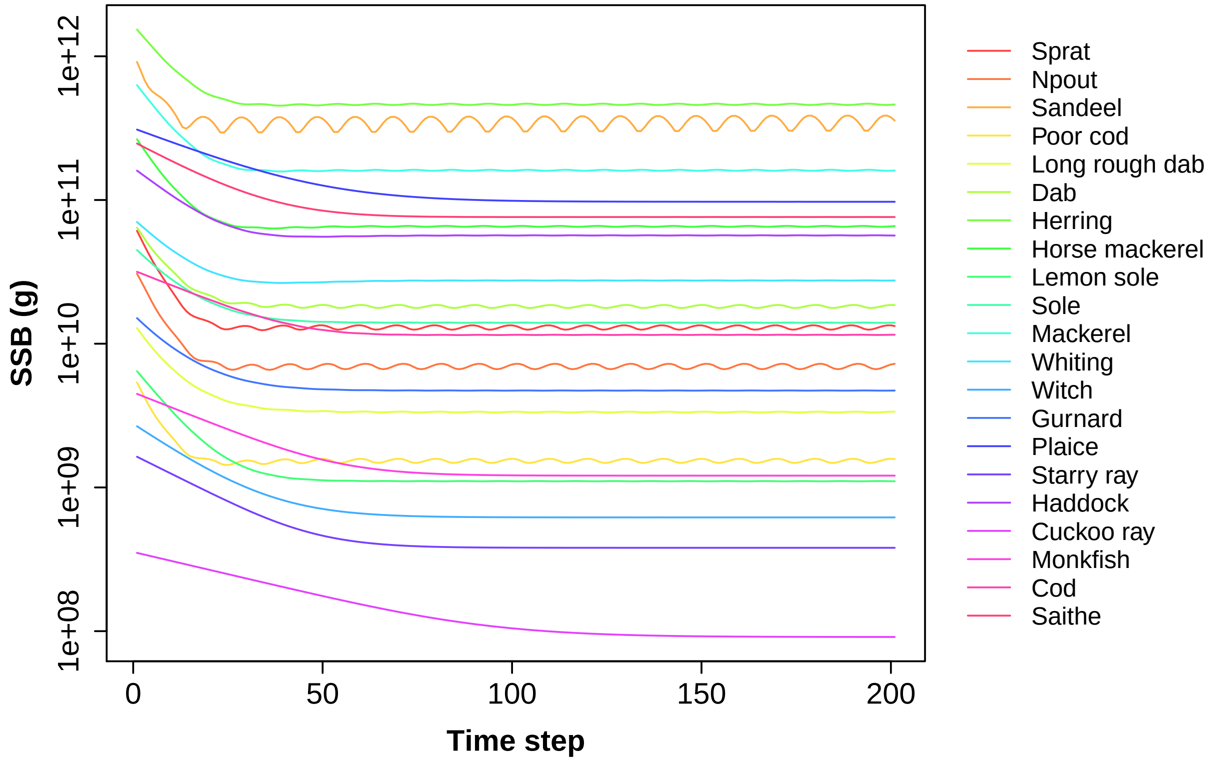

To compare this to the single-species MSY we have to change the rules slightly as we have to specify the fishing mortality rates for the other species. Arbitrarily we choose to set `effort=fmsy` for all the but the ith species when calculating $F_{MSY,i}$. This is:

```
fmsy_lm <- sapply(1:21, function(i, eff){optim(eff[i], calc_catch, i=i, eff=eff,
                                                   method="Brent", lower=0, upper=2,control = list(fnscale = -1
                eff=fmsy)
msy <- run_LeMans(NS_params_n, N0=N0, years=20, effort=matrix(fmsy_lm, nrow=20, ncol=21, byrow=T))
```

We then compare F_{MSY} and F_{Nash} in this study:

```
plot(fmsy_lm, eff, ylab=expression(F[Nash]), xlab=expression(F[MSY]))
abline(a=0, b=1)
```

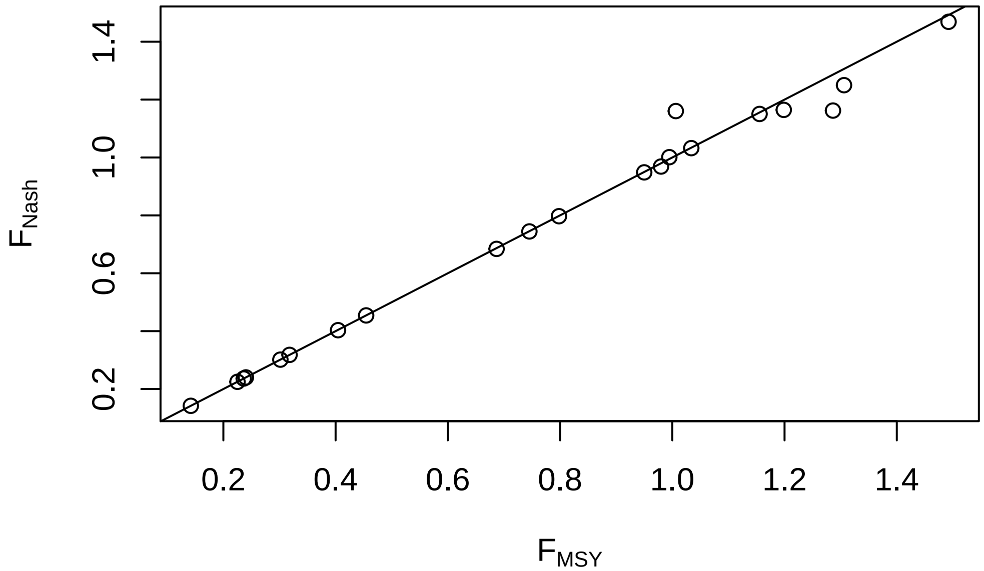

The two appear to be similar for most species, particularly those with lower values of F_{MSY} and F_{Nash}, however they appear to differ more for larger values.

We comment on the arbitrariness of the choice of fishing mortality by pointing out that had we set the other species fishing mortality values to be $F_{Nash,-i}$ when calculating $F_{MSY,i}$, then $F_{MSY,i} = F_{Nash,i}$ by definition.

References

Conner, L., R. Matson, and F.L. Kelly. 2017. "Length-Weight Relationships for Common Freshwater Fish Species in Irish Lakes and Rivers." *Biology and Environment, Proceedings of the Royal Irish Academy* 117B (2): 65–75.

Fulton, E.A., J. Link, I.C. Kaplan, P. Johnson, P. Savina-Rolland, C. Ainsworth, P. Horne, et al. 2011. "Lessons in Modelling and Management of Marine Ecosystems the Atlantis Experience." *Fish and Fisheries* 12: 171–88.

Hall, S.J., J.S. Collie, D.E. Duplisea, S. Jennings, M. Bravington, and J. Link. 2006. "A Length-Based Multispecies Model for Evaluating Community Responses to Fishing." *Canadian Journal of Fisheries and Aquatic Sciences* 63 (6): 1344–59.

Ogle, D.H. 2016. *Introductory Fisheries Analyses with R*. CRC Press.

Polovina, J.J. 1984. "Model of a Coral Reef Ecosystem. I, the ECOPATH Model and Its Application to French Frigate Shoals." *Coral Reefs* 3 (1): 1–11.

Scott, F., J. L. Blanchard, and K.H. Andersen. 2014. "Mizer an R Package for Multispecies, Trait-Based and Community Size Spectrum Ecological Modelling." *Methods in Ecology and Evolution* 5: 1121–5.

Speirs, D., E. Guirey, W. Gurney, and M. Heath. 2010. "A Length-Structured Partial Ecosystem Model for Cod in the North Sea." *Fisheries Research* 106 (3): 474–94.

Spence, M.A., P.G. Blackwell, and J.L. Blanchard. 2016. "Parameter Uncertainty of a Dynamic Multi-Species

Size Spectrum Model." *Canadian Journal of Fisheries and Aquatic Sciences* 73 (4): 589–97.

Thorpe, R.B., P.J. Dolder, S. Reeves, P. Robinson, and S. Jennings. 2016. "Assessing Fishery and Ecological Consequences of Alternate Management Options for Multispecies Fisheries." *ICES Journal of Marine Science* 73 (6): 1503–12.

Thorpe, R.B., W.J.F. LeQuesne, F. Luxford, J.S. Collie, and S. Jennings. 2015. "Evaluation and Management Implications of Uncertainty in a Multispecies Size-Structured Model of Population and Community Responses to Fishing." *Methods in Ecology and Evolution* 6: 49–58.
