# Supplementary material for: LeMaRns: A Length-based Multi-species analysis by numerical simulation in R
Source: PLoS One. 2020 Feb 3;15(2):e0227767. doi: 10.1371/journal.pone.0227767 (PMC6996808; doi:10.1371/journal.pone.0227767)
Supplement: S3 File — Contains an example of calibrating the model with robust uncertainty quantification using a Bayesian framework. (PDF) [file pone.0227767.s003.pdf]

Fitting the North Sea LeMans model to survey and landings data

Hayley J. Bannister and Michael A. Spence

24th September 2019

Introduction

Marine ecosystem models vary in complexity from single-species models with relatively few parameters to multispecies and whole ecosystem models that may include hundreds or even thousands of parameters [1]. Even marine ecosystem models that are considered to be of intermediate complexity may include many hundreds of parameters. For example, the North Sea LeMans model is a length-structured multispecies model of intermediate complexity that is used to simulate the dynamics of the North Sea fish community [2]. The model includes over 500 parameters that describe the 21 fish species that dominate the community [3]. Although many of these parameters can be estimated using empirical data and theoretical relationships, it is often difficult or impossible to identify the value that some of the parameters should take with any degree of certainty [4,5]. Uncertain parameters must therefore be estimated by fitting the model to data [5].

Parameter uncertainty has previously been explored in several marine ecosystem models [2,5,6]. In this document, we adopt the methods of [5] to fit uncertain parameters of the North Sea LeMans model in a Bayesian framework using the R-package `LeMaRns`. Within this framework, we use a Markov chain Monte Carlo (MCMC) algorithm [7] with parallel tempering [8] to sample from the posterior distribution. Before doing so, we use history matching [9] to conduct an initial exploration of the parameter space using a stratified sampling technique in order to identify a starting point for the MCMC algorithm.

The problem

The default values of the parameters of the North Sea LeMans model were originally determined based on life-history theory, stock assessments, trawl surveys, and expert elicitation (see [2] and [3] for further details). However, the model includes several uncertain parameters: the recruitment parameter `b`, which is often referred to as the density-dependent part of the hockey-stick recruitment curve, the 'spin-up' fishing mortality `F0`, and the amount of food (g) available that is not explicitly represented in the model `other`. Importantly, `F0` is typically fixed through time to allow the model to reach a best-fitting equilibrium before time-varying fishing mortalties are introduced based on empirical time series (see below) [5]. `b` and `F0` are species-specific parameters, whilst `other` is species-independent. As there are 21 species in the model, this means that there are 43 parameters (θ) that we would like to estimate using the MCMC algorithm.

In order to fit the LeMans model to data, we require empirical time series that can be used either to drive the model or to compare the model outputs with observations. In this example, we will use a time series of fishing mortalities at age between 1986 and 2017 [10] to drive the model. We will then use a time series of biomass taken from the Greater North Sea International Otter Trawl Q1 survey (extracted from DATRAS [11] and corrected for catchability [12]), as well as a time series of fisheries landings data (extracted from ICES [13]), for comparison purposes. These time series are referred to as $z_{i,t}$ and $w_{i,t}$ for the i-th species in the t-th year respectively. In both cases, we use only the observations recorded between 1986 and 2017 to allow for a direct comparison with the model outputs.

The likelihood of the model is thus:

$$\prod_{t=1986}^{2017} \prod_{i=1}^{21} N(\log(w_{i,t})|c(M_{i,t}(\theta)), \sigma^2_{c,i}) N(\log(z_{i,t})|b(M_{i,t}(\theta)), \sigma^2_{b,i}),$$

where $c(M_{i,t}(\theta))$ is the commercial catch of the i-th species in the t-th year, $b(M_{i,t}(\theta))$ is the commercial catch of the i-th species in the t-th year and N(a|d,e) is a normal density with expectation d and variance e evaluated at a. $\sigma^2_{c,i}$ and $\sigma^2_{b,i}$ will be estimated.

Methods

The model

The `LeMaRns` R-package can be used to set up and run the North Sea LeMans model (see the R-package vignette for further details). The package can be installed and loaded into R as follows:

```
# Install and load LeMaRns R package
install.packages("LeMaRns")
library(LeMaRns)
```

In the `LeMaRns` package, we provide the species-specific parameters of the North Sea model `NS_par` and an interaction matrix `NS_tau`. The `LeMansParam()` function can be used to set up the model:

```
# Set up the North Sea model
NS_params <- LeMansParam(NS_par, tau = NS_tau, eta = rep(0.25, 21),
                         L50 = NS_par$Lmat)
```

To run the model, we must first define the species-, length- and time-varying fishing mortalities `Fs` for each timestep, before calling the `run_LeMans()` function:

```
# Define the number of timesteps the model should be run for
ts <- 500

# Set the fishing mortality to 0 for all species, lengths and timesteps
Fs_array <- array(0, dim = c(NS_params@nsc, NS_params@nfish, ts))

# Run the model
model_run <- run_LeMans(NS_params, tot_time = ts, Fs = Fs_array)
```

Doing so produces outputs such as those shown below:

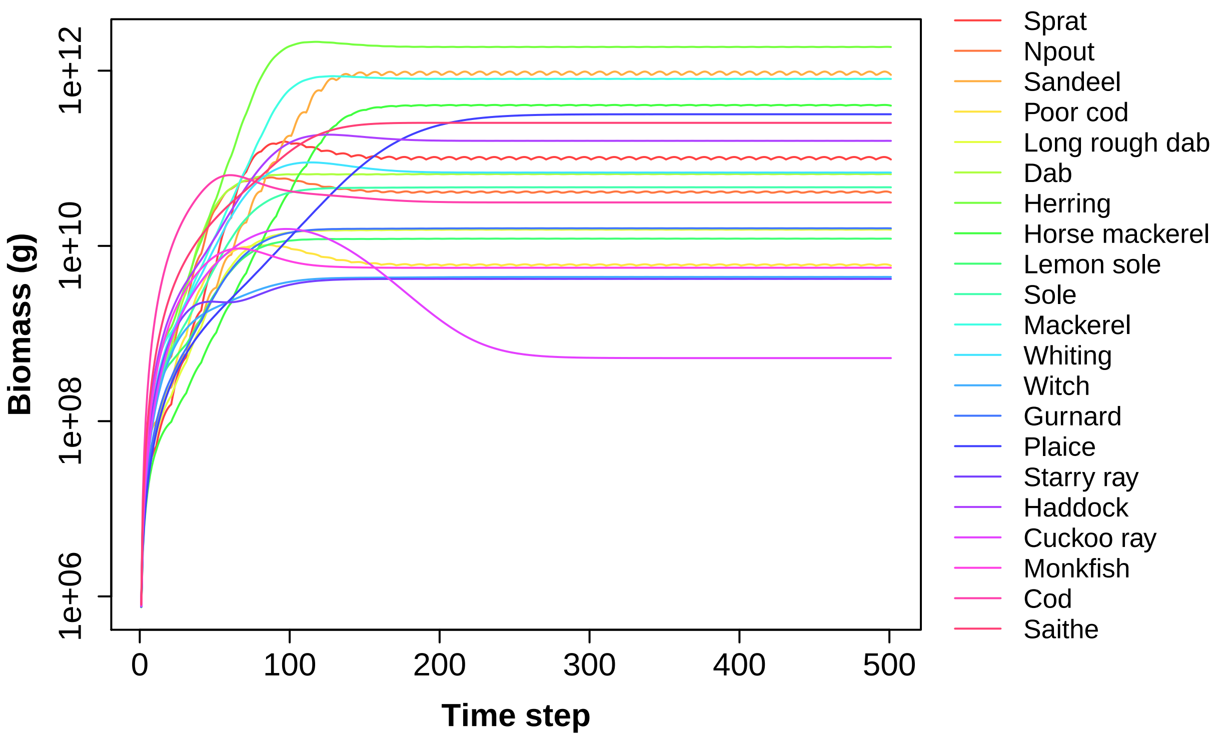

Uncertain parameters

In terms of the uncertain parameters in the model, we will begin with the `b`'s defined in [2], which may be found in `orig_NS_par.RData` (provided as Supplementary Material):

```r
# Load the North Sea parameters
load("orig_NS_par.RData")

# Print orig_NS_par
orig_NS_par
```

```
##      species_names  Linf    W_a    W_b     k  Lmat           a
## 1            Sprat 16.55 0.0059 3.1088 0.681 12.14 885.6667731
## 2            Npout 23.75 0.0075 3.0244 0.849 17.21 225.0988055
## 3          Sandeel 23.57 0.0049 2.7826 1.000 12.16 231.6879245
## 4         Poor cod 23.00 0.0082 3.0865 0.520 15.00 254.2279144
## 5   Long rough dab 25.00 0.0053 3.1434 0.340 15.00 185.3071561
## 6              Dab 32.40 0.0159 2.8639 0.536 12.96  69.3184724
## 7          Herring 33.35 0.0048 3.1984 0.606 23.39  62.1228287
## 8   Horse mackerel 28.00 0.0316 2.6520 0.380 19.00 120.5695838
## 9       Lemon sole 37.00 0.0123 2.9713 0.420 27.00  41.8978427
## 10            Sole 46.41 0.0089 3.0172 0.284 20.96  17.7411663
## 11        Mackerel 38.00 0.0052 3.1674 0.510 26.00  37.8676686
## 12         Whiting 52.50 0.0099 2.9433 0.323 21.39  11.1149557
## 13           Witch 44.00 0.0017 3.3887 0.200 29.00  21.7175060
```

```
## 14        Gurnard  42.97 0.0160 2.8151 0.266 17.67  23.7587503
## 15         Plaice  65.32 0.0125 2.9425 0.122 22.22   4.8535417
## 16     Starry ray  66.00 0.0107 2.9400 0.230 46.00   4.6666095
## 17        Haddock  70.65 0.0092 3.0126 0.271 26.91   3.6046815
## 18     Cuckoo ray  92.00 0.0026 3.2169 0.110 59.00   1.3242529
## 19       Monkfish 106.00 0.0297 2.8410 0.180 61.00   0.7738734
## 20            Cod 149.87 0.0081 3.0502 0.216 54.39   0.2080939
## 21         Saithe 118.94 0.0085 3.0242 0.175 48.61   0.4999956
##               b
## 1  67352.478225
## 2  20821.054248
## 3  57632.695589
## 4  40014.253426
## 5   1710.014437
## 6   3837.179493
## 7  35498.649232
## 8   4672.577765
## 9    208.776950
## 10   320.405464
## 11  1881.553572
## 12  1173.452560
## 13   165.613357
## 14   793.337934
## 15   486.130733
## 16    69.384083
## 17   378.035208
## 18     2.068218
## 19     2.321620
## 20     3.602605
## 21    32.769715
```

`F0` is not explicitly defined in the `LeMaRns` R-package, but it can be incorporated into the array of fishing mortalities `Fs`. A spin-up period is not required to run the model and therefore there are no default values for this parameter. The default value of `other` is `1e12` (based on [2]), although this can only be viewed by accessing the help file of the `LeMansParam()` function (i.e. by calling `?LeMansParam`).

Initial exploration

The first step in the fitting procedure involves using history matching [9] to conduct an initial exploration of the parameter space. The aim of this step is to identify a set of parameter values that may act as a starting point for the MCMC algorithm.

Generating the parameter sets

To do this, we use a Sobol' sequence that can be generated using the `randtoolbox` package. In this example, we generate a Sobol' sequence with 43 columns (i.e. the number of uncertain parameters that we want to estimate) and 5000 rows (i.e. the number of model evaluations that we would like to conduct during our initial exploration of the parameter space):

```r
# Install and load the randtoolbox package
install.packages("randtoolbox")
library(randtoolbox)
```

```r
# Set the seed for reproducibility
set.seed(710)

# Generate a Sobol' sequence
sobol_seq <- sobol(n = 5000, dim = 43)
```

By plotting the first two columns of the Sobol' sequence, we can see that it produces values between zero and one for each parameter:

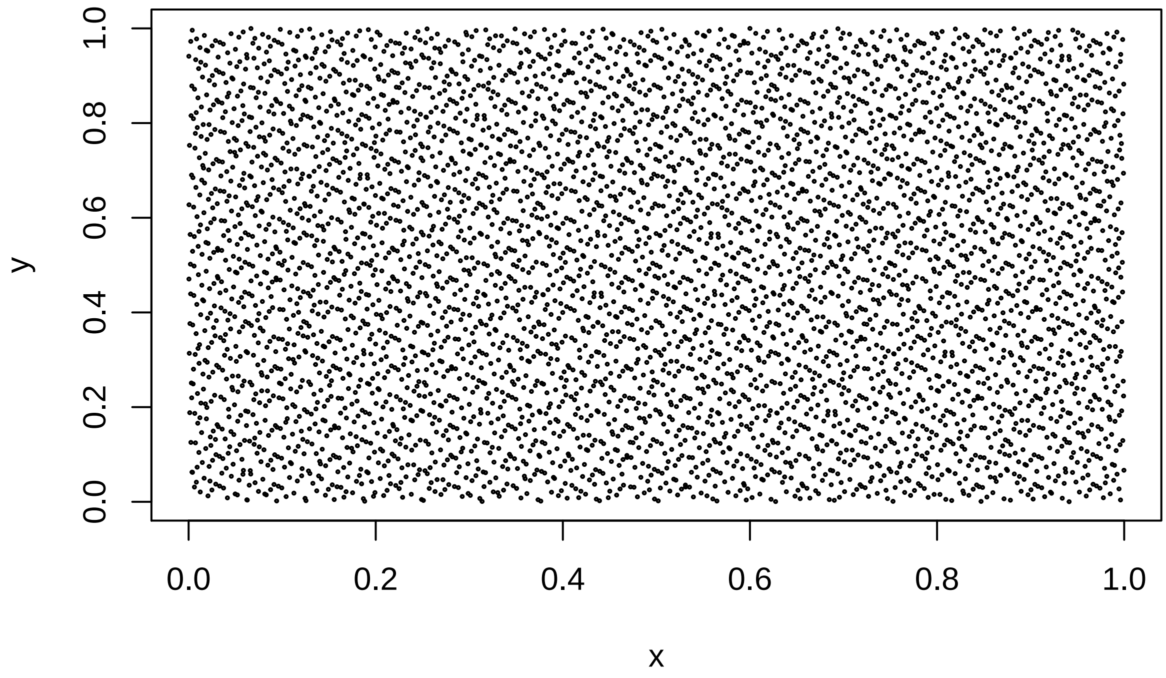

We must therefore transform the Sobol' sequence onto the scale of the parameters. Doing so requires us to assign a distribution to each of our uncertain parameters. In this example, we apply a uniform distribution to the recruitment parameter `b` with upper and lower limits of ± 100% of the default values. We define the upper and lower limits on the logarithmic scale in order to undersample combinations of extremely high parameter values:

```r
# Define the lower limits of b
b_lower <- log(orig_NS_par$b)+log(0.001)
b_lower
```

```
##  [1]  4.2099397  3.0359647  4.0540900  3.6892357  0.5365018  1.3447376
##  [7]  3.5694946  1.5417109 -1.5664888 -1.1381680  0.6320978  0.1599503
## [13] -1.7980994 -0.2315060 -0.7212777 -2.6680978 -0.9727679 -6.1810678
## [19] -6.0654900 -5.6260980 -3.4182505
```

```r
# Define the upper limits of b
b_upper <- log(orig_NS_par$b)+log(2)
b_upper
```

```
##  [1] 11.810842 10.636867 11.654992 11.290138  8.137404  8.945640 11.170397
##  [8]  9.142613  6.034414  6.462734  8.233000  7.760853  5.802803  7.369396
## [15]  6.879625  4.932805  6.628135  1.419835  1.535412  1.974805  4.182652
```

We can then rescale the first 21 columns of the Sobol' sequence to define the values of `b` that we will use during our initial exploration of the parameter space:

```r
# Rescale the first 21 columns of the Sobol' sequence based on the lower and upper
# limits of b
for (i in 1:21) {
  sobol_seq[, i] <- b_lower[i]+(b_upper[i]-b_lower[i])*sobol_seq[, i]
}
```

By again plotting the first two columns of the Sobol' sequence, we can see that we have successfully undersampled combinations of extremely high parameter values:

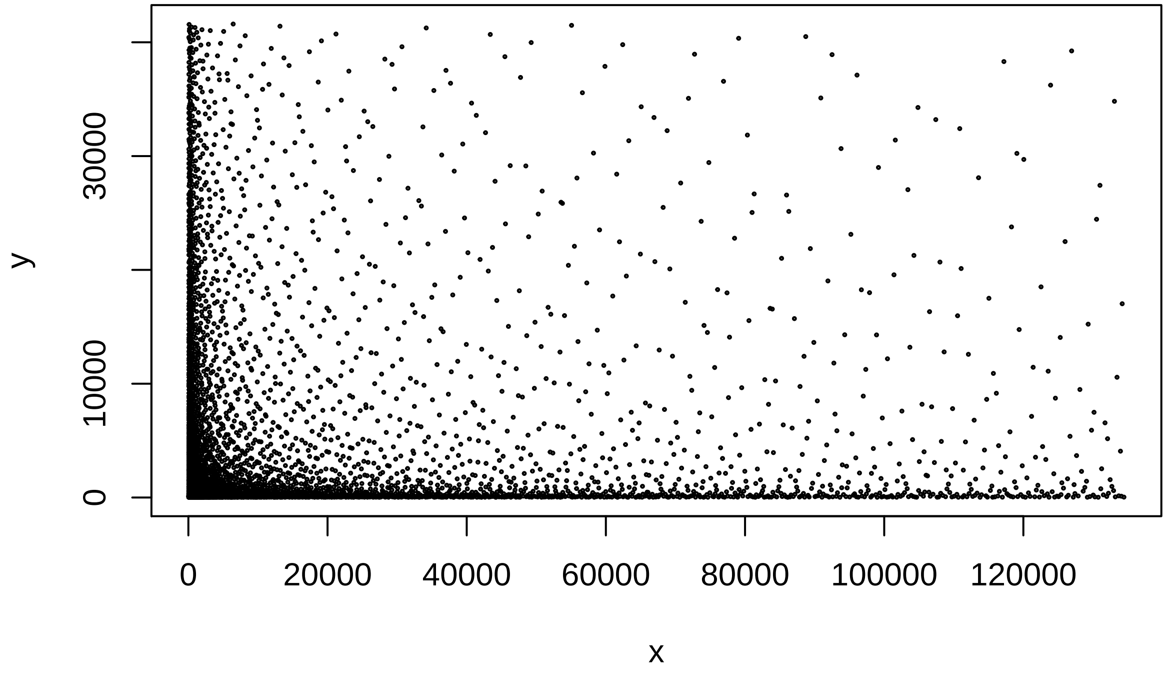

`F0` is not associated with any default values, but it does not make sense for this parameter to be negative [5]. Because of this, we will allow `F0` to vary between zero and two for each species. We can therefore rescale the columns of the Sobol' sequence that we will be assigning to `F0` using a multiplier of two:

```r
# Rescale the next 21 columns of the Sobol' sequence using a multiplier of 2
for (j in 22:42) {
  sobol_seq[, j] <- sobol_seq[, j]*2
}
```

Finally, we will assume that `other` may vary between 10 and 30 (on the logarithmic scale):

```r
# Rescale the last column of the Sobol' sequence based on the lower and upper
# limits of other
sobol_seq[, 43] <- 10+(30-10)*sobol_seq[, 43]
```

Running the model

We now have 5000 parameter sets with which to run the model. However, doing so requires us to create a function that takes the Sobol' sequence as input, runs the model, and extracts the outputs of interest. In this example, the function may be defined as:

```r
# Define a function to run the North Sea LeMans model based on the parameter values
# given by a Sobol' sequence
run_LeMaRns <- function(x, parameter_sets) {
  # Load required packages
  require(LeMaRns)
  require(dplyr) # NB: dplyr comes from the tidyverse package

  # Set up the model with the x-th value of other
  NS_params <- LeMansParam(NS_par, tau = NS_tau, eta = rep(0.25, 21),
                           L50 = NS_par$Lmat, other = exp(parameter_sets[x, 43]))

  # Insert the recruitment parameters using the b's given by the Sobol' sequence
  a <- NS_par$a
  b <- exp(parameter_sets[x, 1:21])
  NS_params@recruit_params <- do.call("Map", c(c, list(a = a, b = b)))

  ################################################################################
  # Pre-run the model with no fishing (50 years)
  ################################################################################
  # Define the number of timesteps in the no fishing period
  nofish_time <- 50*(1/NS_params@phi_min)

  # Set up the Fs for the no fishing period
  nofish_Fs <- array(0, dim = c(NS_params@nsc, NS_params@nfish, nofish_time))

  # Run the model
  pre_run <- run_LeMans(NS_params, Fs = nofish_Fs, tot_time = nofish_time)

  ################################################################################
  # Set up the main model run
  ################################################################################
  # Define the number of years in the spin-up period
  spin_up <- 50

  # Define the number of years in the observed fishing period
```

```r
fishing <- length(1986:2017)

# Calculate the total number of years
years <- spin_up + fishing

# Calculate the total number of timesteps
n_timesteps <- years*(1/NS_params@phi_min)

# Define the Fs for the main model run. To do this, first set up an empty array to
# save the Fs to and then load the ICES Fs that are provided alongside this tutorial
Fs_array <- array(0, dim = c(NS_params@nsc, NS_params@nfish, n_timesteps))
load("ICES_Fs.RData")

# Set the Fs for the observed fishing period
for (t in 1:fishing) {
  # Define the timesteps associated with the observed fishing period
  max_ts <- ((1/NS_params@phi_min)*spin_up)+(1/NS_params@phi_min*t)
  min_ts <- (max_ts - (1/NS_params@phi_min))+1

  # Set the Fs based on the ICES data
  Fs_array[,,min_ts:max_ts] <- as.matrix(ICES_Fs[[t]]/(1/NS_params@phi_min))
}

# Set the Fs for the spin-up fishing period
Fs_array[,,1:((1/NS_params@phi_min)*spin_up)] <-
  Fs_array[,,((1/NS_params@phi_min)*spin_up)+1] %*%
  diag(parameter_sets[x, 22:42])

##################################################################################
# Run the model and extract the outputs
##################################################################################
# Run the model (continuing on from the pre-run with no fishing)
model_run <- run_LeMans(N = pre_run@N[,,dim(pre_run@N)[3]], NS_params,
                        Fs = Fs_array, tot_time = n_timesteps)

# Combine the two model runs
model_run <- comb_LeMans_run(pre_run, model_run, cont = TRUE)

# Extract catch and biomass outputs
catch <- data.frame(t(colSums(model_run@Catch)))
biomass <- data.frame(get_biomass(NS_params, model_run))

# Add column names
names(catch) <- NS_params@species_names
names(biomass) <- NS_params@species_names

# Extract required rows and change units of catch to tonnes
catch <- catch[1002:nrow(catch), ]/1e6
biomass <- biomass[1002:nrow(biomass), ]

# Insert 'Year' column
catch$Year <- rep(1986:2017, each = 10)
biomass$Year <- rep(1986:2017, each = 10)
```

```
  # Calculate the total catch and biomass of each species per year
  catch <- catch %>%
    group_by(Year) %>%
    summarise_all(list(sum), na.rm = T)

  biomass <- biomass %>%
    group_by(Year) %>%
    summarise_all(list(sum), na.rm = T)

  # Combine the outputs into a list
  out <- list(catch = catch, biomass = biomass)
  return(out)
}
```

In this function, we take the parameter values from a single row of the Sobol' sequence (defined by the parameter `x`) and insert them into the model. We run the model with three different sets of fishing mortalities. Firstly, we run the model for 50 years with no fishing. We then continue the model evaluation with a 50 year spin-up period within which the fishing mortalities are held constant at the levels defined by `F0`. In the last part of the model evaluation we use the empirical time series of fishing mortalities in the North Sea between 1986 and 2017, which is stored in `ICES_Fs_for_LeMaRns.RData`. Once the model evaluation is complete, we extract the annual biomass and catch of each species between 1986 and 2017 from the model outputs.

Please note that although it is possible to increase the efficiency of this function, we have attempted to define it in a way that maximises both clarity and efficiency. The model evaluations may also be run in parallel to further increase efficiency:

```
# Install and load the parallel package
install.packages("parallel")
library(parallel)

# Set up cluster using the total number of cores on your machine minus 1
no_cores <- detectCores()-1
clus <- makeCluster(no_cores)

# Run the run_LeMaRns() function using the 5000 parameter sets given by sobol_seq
LeMaRns_out <- parLapply(cl = clus, 1:5000, run_LeMaRns, parameter_sets = sobol_seq)

# Stop the cluster
stopCluster(clus)
```

Comparing the model outputs with observations

Once the model evaluations are complete, we can compare the outputs of the model with observations. In this example, we use observations taken from the Greater North Sea International Otter Trawl Q1 survey ([11]; corrected for catchability [12]) and ICES fisheries landings data [13], which are stored in "`survey_biomass.RData`" and "`fisheries_landings.RData`" respectively:

```
# Load survey data and display the first 5 rows and columns
load("survey_biomass.RData")
head(survey_biomass[, 1:5])
```

```
##   Year     Sprat    Npout   Sandeel  Poor cod
```

```
## 1 1986  44078.04 234565.2 16084.781  45828.92
## 2 1987 165546.97 368737.7 16117.810 124436.06
## 3 1988 285344.13 153316.5  1949.848  48024.97
## 4 1989 528140.52 273912.6 21513.856  80069.31
## 5 1990 224601.26 267426.2 18213.445  73419.90
## 6 1991 230471.87 389646.7  5424.603 112793.19
```

```r
# Load fisheries landings data and display the first 5 rows and columns
load("fisheries_landings.RData")
head(fisheries_landings[, 1:5])
```

```
##   Year Sprat  Npout Sandeel Poor cod
## 1 1986 53854 226857  868204        0
## 2 1987 78056 215186  837970        0
## 3 1988 92995 187113  994556        0
## 4 1989 50269 275875 1098061        0
## 5 1990 50688 211574  720985        0
## 6 1991 91753 223473  987248        0
```

Please note that any observations with zero landings must be replaced with `NA`s to avoid issues when transforming the observations on to the logarithmic scale (see below). In this example, we also need to replace the 2005 landings data for Norway pout with a `NA` as the fishing mortality of Norway pout is set to zero in the model in 2005 and therefore the model always records a catch of zero during this period:

```r
# Change zero landings to NA
for (k in 1:ncol(fisheries_landings)) {
  fisheries_landings[which(fisheries_landings[, k] == 0), k] <- NA
}

# Change 2005 landings of Norway pout to NA as there are zero catches
fisheries_landings$Npout[20] <- NA
```

We can now calculate the sum of the squared differences between the observed and modelled biomass `biomass_SS` :

```r
# Calculate sum of squared differences for biomass
biomass_SS <- lapply(1:5000, function(x) {
  colSums((log(survey_biomass) - log(LeMaRns_out[[x]][["biomass"]]))^2, na.rm = T)
})
biomass_SS <-  data.frame(do.call("rbind", biomass_SS), check.names = FALSE)
```

and we can also do the same for catch `catch_SS`:

```r
# Calculate the sum of the squared differences for landings/catch
catch_SS <- lapply(1:5000, function(x) {
  colSums((log(fisheries_landings) - log(LeMaRns_out[[x]][["catch"]]))^2, na.rm = T)
})
catch_SS <-  data.frame(do.call("rbind", catch_SS), check.names = FALSE)
```

The product of `biomass_SS` and `catch_SS` can then be used to give an overall measure of model fit `prod_SS` for each model evaluation:

```r
# Calculate the product of biomass_SS and catch_SS
prod_SS <- biomass_SS*catch_SS
```

Comparing model fit

Next, we need to merge `prod_SS` with the corresponding parameter values that were used to run the model and then plot them against one another. We will do this separately for the species-specific parameters `b` and `F0` and for the species-independent parameter `other` for simplicity. To do this, we must first re-format `prod_SS` and `sobol_seq`:

```r
# Add in a column to prod_SS that specifies which parameter set each row relates to
prod_SS$parameter_set <- 1:nrow(prod_SS)

# Re-format prod_SS from wide to long format
prod_SS <- gather(select(prod_SS, -Year), species, SS, -parameter_set)

# Add in a column to sobol_seq that specifies which parameter set each row relates to
sobol_seq <- data.frame(sobol_seq)
sobol_seq$parameter_set <- 1:nrow(sobol_seq)

# Remove the last column of sobol_seq as this column relates to the species-independent
# parameter 'other', which we will deal with later
sobol_seq_subset <- sobol_seq[, -43]

# Rename the columns of sobol_seq_subset with the names of the species and parameters
# they relate to
names(sobol_seq_subset) <- c(paste(NS_params@species_names,
                             rep(c("b", "F0"), each = 21), sep = "_"), "parameter_set")

# Re-format sobol_seq_subset from wide to long format
sobol_seq_subset <- gather(sobol_seq_subset, sp_param, parameter_value, -parameter_set)

# Split the sp_param column into separate species and parameter columns
labels <- data.frame(do.call("rbind", str_split(sobol_seq_subset$sp_param, "_")))
names(labels) <- c("species", "param") # label the new columns
sobol_seq_subset <- cbind(sobol_seq_subset, labels) # add the new columns back into
# sobol_seq_subset

# Remove the unnecessary sp_param column and re-format sobol_seq_subset to create a
# separate column for each parameter
sobol_seq_subset <- select(sobol_seq_subset, -sp_param)
sobol_seq_subset <- spread(sobol_seq_subset, param, parameter_value)
```

We can then merge `prod_SS` and `sobol_seq_subset` by `parameter_set` and `species`:

```r
# Merge prod_SS and sobol_seq_subset
SS <- merge(prod_SS, sobol_seq_subset, by = c("parameter_set", "species"))
```

```r
# Display the first 10 rows of SS
head(SS, 10)
```

```
##    parameter_set        species             SS          b F0
```

```
## 1              1            Cod 1.306142e+05 -1.825647  1
## 2              1     Cuckoo ray 2.093225e+03 -2.380617  1
## 3              1            Dab 1.293788e+10  5.145189  1
## 4              1        Gurnard 5.817832e+10  3.568945  1
## 5              1        Haddock 7.084931e+06  2.827683  1
## 6              1        Herring 3.168762e+04  7.369946  1
## 7              1 Horse mackerel 1.355125e+09  5.342162  1
## 8              1     Lemon sole 2.427217e+10  2.233962  1
## 9              1 Long rough dab 3.623867e+11  4.336953  1
## 10             1       Mackerel 2.274672e+05  4.432549  1
```

The final step is to examine plots of the parameter values used in each model evaluation against the corresponding measure of model fit `SS` for each species. The aim is to identify parameter values that result in the **lowest** `SS`:

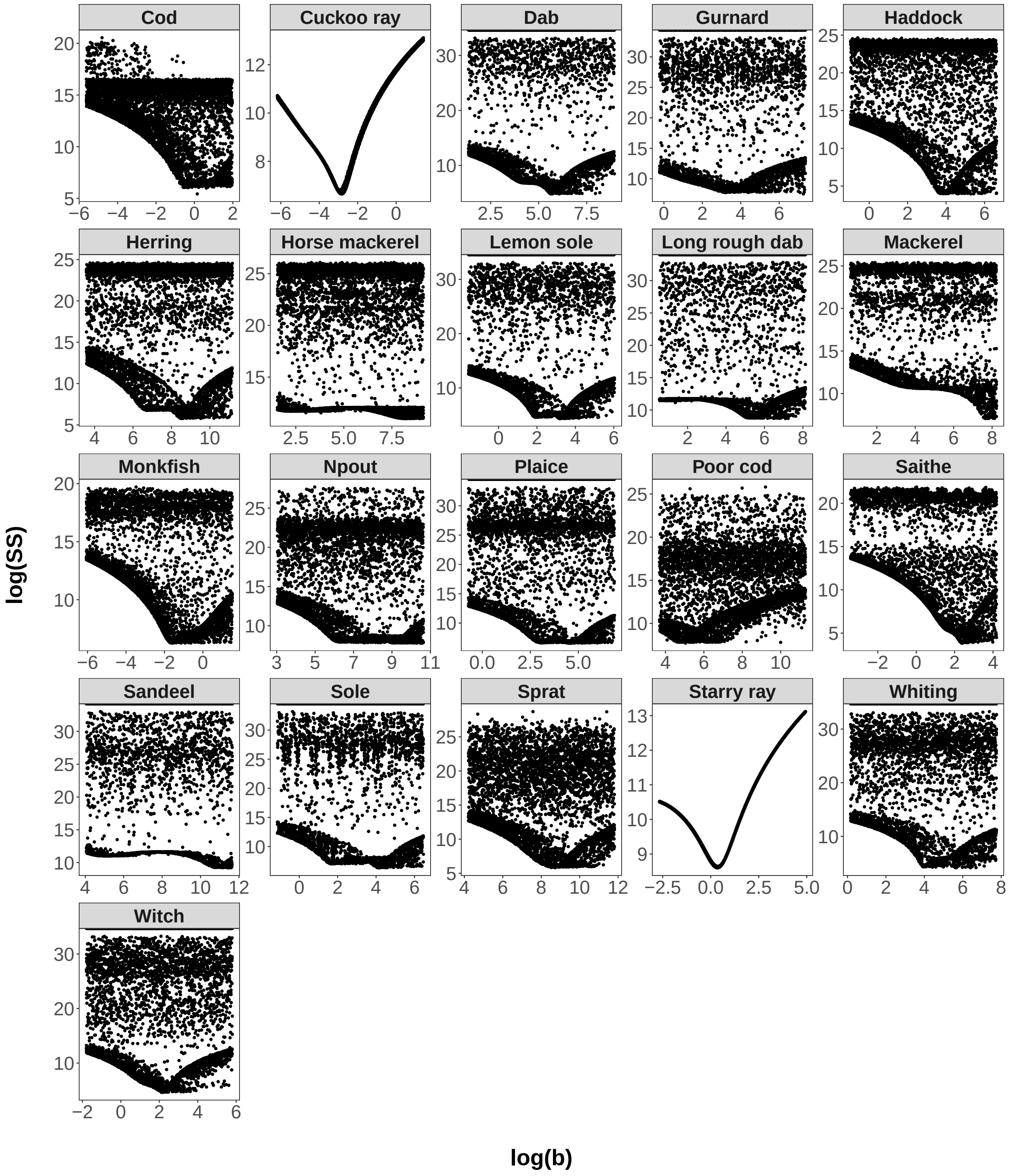

Despite there being a lot of noise, there is a clear pattern in the `SS` associated with each species. The majority have a well-defined minimum that is highlighted by a line in the plot below:

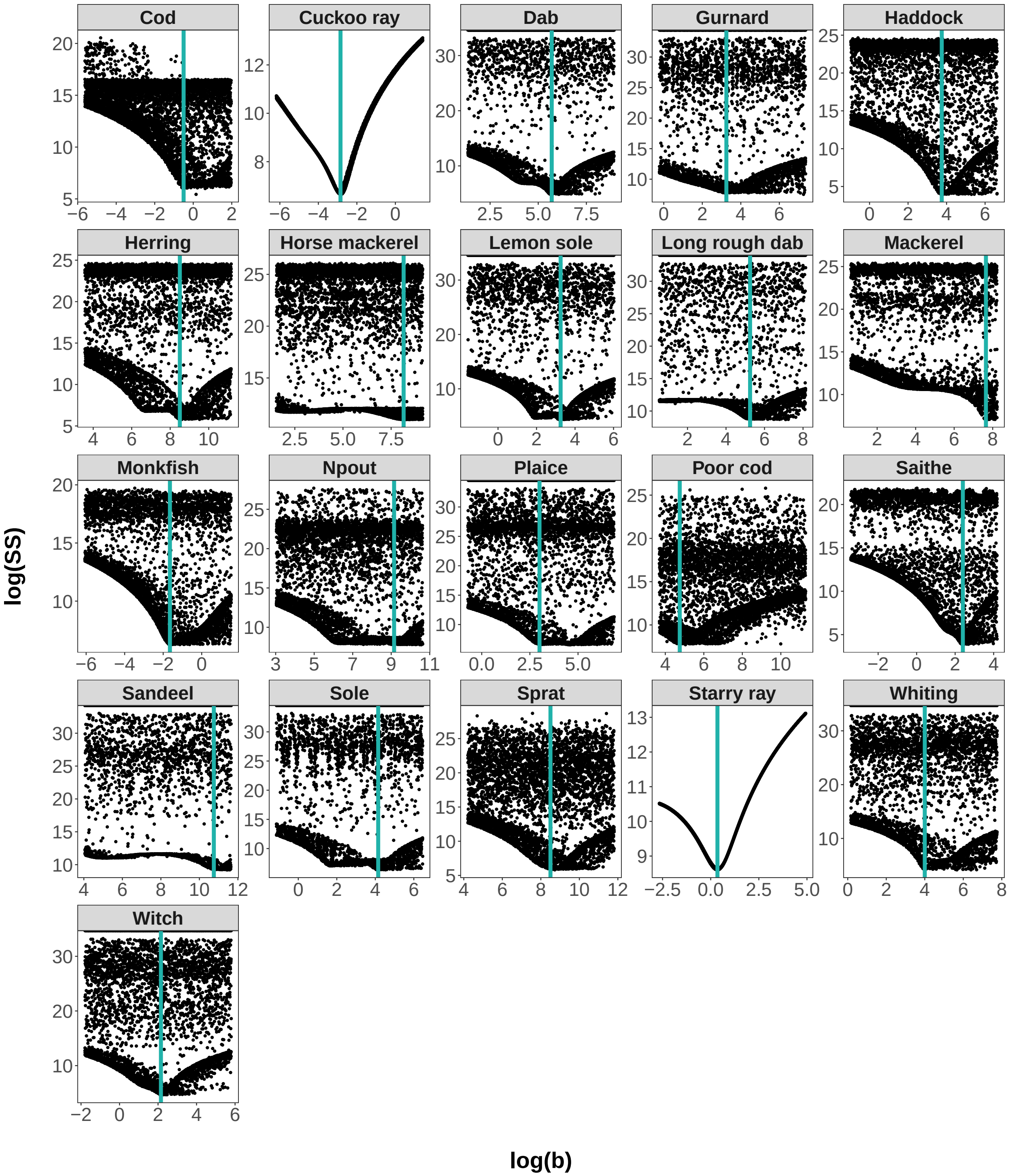

We can now assess a similar plot for `F0`:

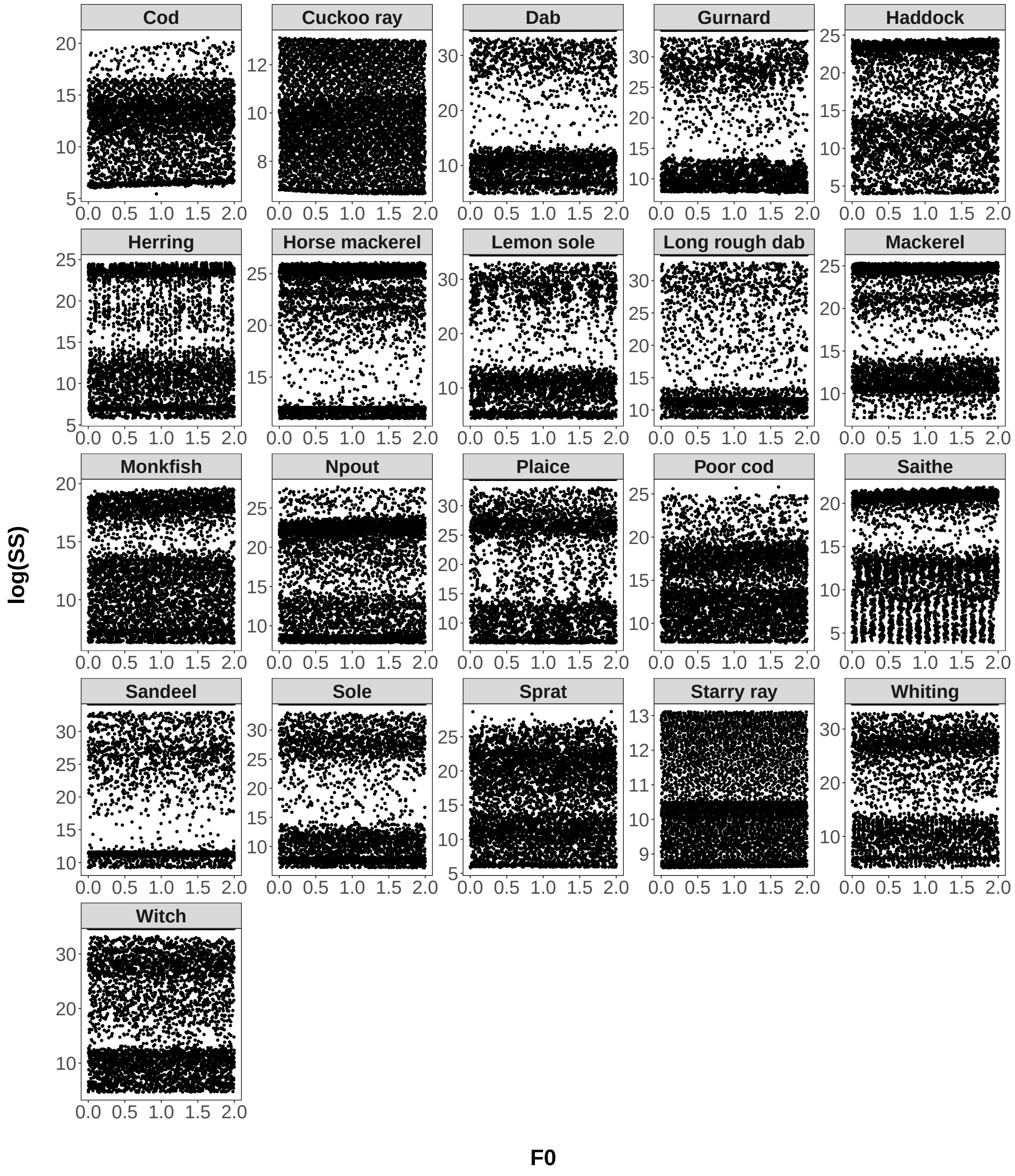

For most species, `F0` can take almost any value without having an effect on model fit. However, for some species the model fit declines with increasingly large values of `F0`. An intermediate value of one may thus be a good starting point for the MCMC algorithm for all species.

To produce the same plot as above for `other`, we must again merge our measure of model fit `prod_SS` with the corresponding values of `other`:

```r
# Extract the last two columns of sobol_seq as they represent the parameter 'other'
# and the ID number of the parameter set
sobol_seq_other <- data.frame(other = sobol_seq[, (ncol(sobol_seq)-1)],
                              parameter_set = sobol_seq[ncol(sobol_seq)])

# Merge prod_SS and sobol_seq_other
SS_other <- merge(prod_SS, sobol_seq_other, by = c("parameter_set"))
```

```r
# Display the first 10 rows
head(SS_other)
```

```
##   parameter_set species          SS other
## 1             1   Sprat  2531732384    20
## 2             1 Gurnard 58178322591    20
## 3             1 Whiting 74140080278    20
## 4             1 Haddock     7084931    20
## 5             1     Dab 12937882723    20
## 6             1  Plaice 17195931869    20
```

We can now plot `other` against our measure of model fit `SS`:

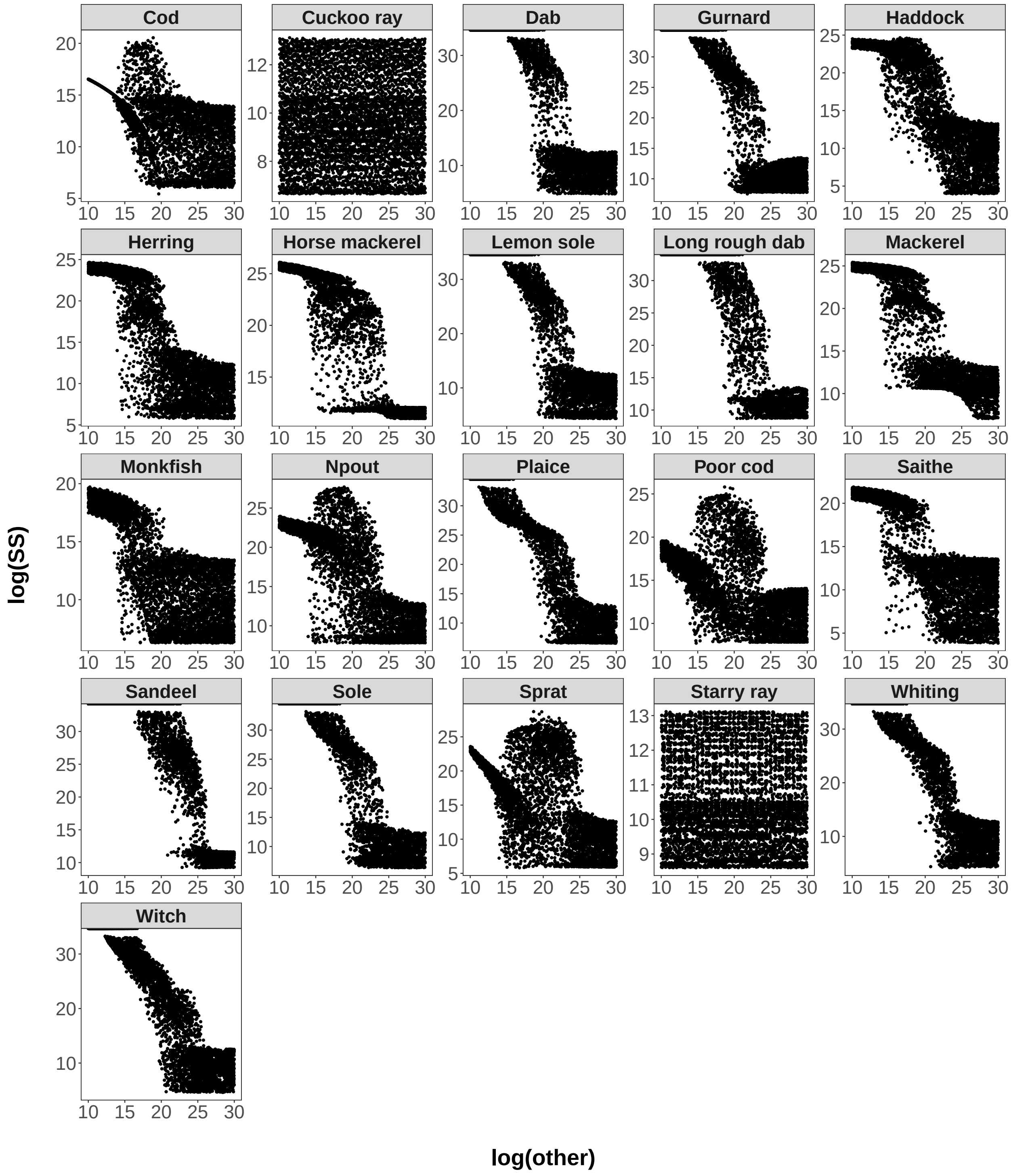

As you can see, `other` cannot be smaller than 27.5.

MCMC algorithm

Initial parameter values

Now we have a set of initial values with which to begin with in the MCMC algorithm:

```r
b <- c(8.5, 9.15, 10.75, 4.75, 5.25, 5.7, 8.5, 8.15, 3.25, 4.15, 7.65,
       4, 2.15, 3.25, 3, 0.35, 3.75, -2.85, -1.65, -0.5, 2.4) # on the logarithmic scale
F0 <- rep(1, 21)
other <- 27.5 # on the logarithmic scale
```

For the purpose of running the algorithm, we will combine these parameter values into a single vector `theta`:

```r
theta <- c(b, F0, other)
```

Next, we need to define the initial variance parameters for the survey biomass `sigma_sq_biomass` and fisheries landings `sigma_sq_catch` data (on the logarithmic scale). To begin with, we will assume the variance of the error is equal to one for all species:

```r
# Define the initial variance parameters
sigma_sq_biomass <- rep(1, 21)
sigma_sq_catch <- rep(1, 21)
```

However, we will increase `sigma_sq_biomass` for pelagic species as they are not well sampled during trawl surveys. We will also increase `sigma_sq_catch` for species that are not subject to a full stock assessment:

```r
# Increase the initial variance parameters for some species
sigma_sq_biomass[c(1, 3, 7, 8, 11)] <- 2 # pelagic species
sigma_sq_catch[c(4, 5, 6, 9, 13, 16, 18)] <- 2 # species without full assessments
```

This results in the following variance parameters:

```
##            species sigma_sq_biomass sigma_sq_catch
## 1            Sprat                2              1
## 2            Npout                1              1
## 3          Sandeel                2              1
## 4         Poor cod                1              2
## 5   Long rough dab                1              2
## 6              Dab                1              2
## 7          Herring                2              1
## 8   Horse mackerel                2              1
## 9       Lemon sole                1              2
## 10            Sole                1              1
## 11        Mackerel                2              1
## 12         Whiting                1              1
## 13           Witch                1              2
## 14         Gurnard                1              1
## 15          Plaice                1              1
## 16      Starry ray                1              2
## 17         Haddock                1              1
## 18      Cuckoo ray                1              2
## 19        Monkfish                1              1
## 20             Cod                1              1
## 21          Saithe                1              1
```

We also need to define parameters that can be used to determine the step size when proposing a new `theta` within the algorithm. During each iteration of the algorithm, we will first propose a new `theta` that includes

only a change in other. We will then choose whether to accept or reject the new theta. Next, we will propose a new theta that includes a change in both the b and F0 of a given species and we will repeat this step for all 21 species in the model. This means that we need two step size parameters - one for the change in other (sigma_other) and one for the change in both b and F0 (sigma_spec):

```
# Define sigma_other and sigma_spec
sigma_other <- 0.9230153
sigma_spec <- cbind(c(0.31393400, 0.28181273, 0.33200897, 0.32037283, 0.16427169,
                      0.12908588, 0.18226098, 0.47733376, 0.09342795, 0.11632263,
                      0.15524919, 0.19488468, 0.12578285, 0.23721283, 0.21863908,
                      0.66168103, 0.28455524, 0.20124415, 0.19455011, 0.38354228,
                      0.09573732),
                    c(1.1160157, 1.1190475, 1.1323313, 1.0646912, 1.0958114,
                      1.1092258, 1.0900947, 1.1376135, 0.8328927, 1.1060205,
                      0.9178471, 0.5646299, 0.5068310, 0.8367190, 0.6196585,
                      1.0539679, 0.8717514, 0.6735339, 1.1180255, 0.1734129,
                      0.5110077))
```

These values were determined by trial-and-error based on running the algorithm several times and evaluating how well the algorithm moves around the parameter space using different step sizes.

We can now set the maximum number of iterations to run the algorithm for:

```
# Set the maximum number of iterations
iter <- 5000
```

Prior and likelihood functions

Now we need to define the prior and likelihood functions:

```
# Define the prior function
l_prior <- function(theta) {
  # F0
  sum(dunif(theta[22:42], min = 0, max = 2, log = TRUE)) +

  # other
  sum(dunif(theta[43], min = 20, max = 30, log = TRUE))
}

# Define the likelihood function
l_likelihood <- function(model_outputs, fisheries_landings, survey_biomass,
                         sigma_sq_catch, sigma_sq_biomass) {
  # Landings/catch
  sum(dnorm(x = 0,
            mean = t(log(fisheries_landings[, -1])-
                       log(model_outputs[["catch"]][, -1])),
            sd = sqrt(sigma_sq_catch), log = TRUE), na.rm = TRUE) +

  # Biomass
  sum(dnorm(x = 0,
            mean = t(log(survey_biomass[, -1])-
                       log(model_outputs[["biomass"]][, -1])),
            sd = sqrt(sigma_sq_biomass), log = TRUE))
}
```

The prior represents our subjective beliefs about the unknown parameter `theta`. In this example, we believe `F0` (which is defined in `theta[22:42]`) lies between zero and two for all species and `other` (which is defined in `theta[43]`) lies between 20 and 30 (on the logarithmic scale). In both cases, we specify a uniform distribution such that all values between the specified ranges are equally likely.

The likelihood represents the probability of the observed data (i.e. the survey biomass and fisheries landings data) conditional on `theta`. The aim of the MCMC algorithm is to sample from the posterior distribution.

Initialisation

In the final step before running the algorithm, we must initialise the model using the starting values defined in `theta` and calculate the likelihood. To run the model, we will use a slightly modified version of the `run_LeMaRns()` function that we defined earlier:

```
# Define the modified version of the run_LeMaRns() function
run_LeMaRns_MCMC <- function(NS_params, parameters, Fs) {
  # Load required packages
  require(LeMaRns)
  require(dplyr) # NB: dplyr comes from the tidyverse package

  # Insert 'other'
  NS_params@other <- exp(parameters[43])

  # Insert the recruitment parameters
  a <- NS_par$a
  b <- exp(parameters[1:21])
  NS_params@recruit_params <- do.call("Map", c(c, list(a = a, b = b)))

  # Insert the Fs for the spin-up fishing period
  spin_up <- 50
  Fs[,,501:((1/NS_params@phi_min)*(no_fish+spin_up))] <-
    Fs[,,((1/NS_params@phi_min)*(no_fish+spin_up))+1] %*%
    diag(parameters[22:42])

  # Run the model
  model_run <- run_LeMans(NS_params, Fs = Fs, tot_time = dim(Fs)[3])

  # Extract catch and biomass outputs and convert to tonnes
  catch <- data.frame(t(colSums(model_run@Catch)))/1e6
  biomass <- data.frame(get_biomass(NS_params, model_run))/1e6

  # Extract model outputs between 1986 and 2017
  catch <- catch[1002:nrow(catch), ]
  catch$Year <- rep(1986:2017, each = 10)

  biomass <- biomass[1002:nrow(biomass), ]
  biomass$Year <- rep(1986:2017, each = 10)

  # Calculate the total catch and biomass of each species per year
  catch <- catch %>%
    group_by(Year) %>%
    summarise_all(list(sum), na.rm = T) %>%
    as.matrix()
```

```
  biomass <- biomass %>%
    group_by(Year) %>%
    summarise_all(list(sum), na.rm = T) %>%
    as.matrix()

  # Combine the outputs into a list
  out <- list(catch = catch, biomass = biomass)
  return(out)
}
```

One of the most notable changes in the modified version of the function defined above is that it accepts only one parameter set (i.e. `theta`) rather than a Sobol' sequence. This means the function no longer requires the parameter `x`. As both the 'no fishing' and 'observed fishing' periods do not change between model evaluations, the fishing mortalities `Fs` may be partially defined outside the function to reduce the run time of the algorithm. The modified version of the function therefore also takes `Fs` as input. These `Fs` can be defined using a similar method as in the original `run_LeMaRns()` function:

```
# Set the number of years and timesteps ('tot_time') for the main model run
no_fish <- 50
spin_up <- 50
fishing <- length(1986:2017)
years <- no_fish + spin_up + fishing
n_timesteps <- years*(1/NS_params@phi_min)

# Set up an empty array to store the Fs
Fs_array <- array(NA, dim = c(NS_params@nsc, NS_params@nfish, n_timesteps))

# Set up the no fishing period (50 years)
Fs_array[,,1:((1/NS_params@phi_min)*no_fish)] <- 0

# Set up the observed fishing period (1986 - 2017)
load("ICES_Fs.RData")
for (t in 1:fishing) {
  max_ts <- ((1/NS_params@phi_min)*(no_fish+spin_up))+(1/NS_params@phi_min*t)
  min_ts <- (max_ts - (1/NS_params@phi_min))+1
  Fs_array[,,min_ts:max_ts] <- as.matrix(ICES_Fs[[t]]/(1/NS_params@phi_min))
}
```

Next, we initialise the model and calculate the likelihood:

```
# Initialise model
model_outputs <- run_LeMaRns_MCMC(NS_params, theta, Fs_array)

# Calculate likelihood
l_like <- l_likelihood(model_outputs, fisheries_landings, survey_biomass,
                       sigma_sq_catch, sigma_sq_biomass)
l_like
```

```
## [1] -3237.093
```

Parallel tempering

As we suspect the posterior distribution is multi-modal, we will use an MCMC algorithm with parallel tempering [8]. Parallel tempering involves running several chains in parallel with different 'temperatures'. The 'temperature' determines the shape of the distribution, with greater values resulting in a more peaked distribution and a value of zero resulting in a flat line [14]. Lower temperatures allow the algorithm to make larger steps whilst still maintaining good mixing properties [14]. Note: 'mixing' refers to the amount of time required for the chain to reach a steady-state. After a specified number of iterations, the chains may exchange points, thus allowing the algorithm to move around and between the modes of the posterior distribution more easily [14].

In order to implement this procedure we must first define a function that can be used to run several chains in parallel. This function requires the following inputs: the parameter values `theta`, the model outputs and corresponding log-likelihood (`model_outputs` and `l_like`), the initial variance parameters (`sigma_spec`, `sigma_other`, `sigma_sq_catch`, and `sigma_sq_biomass`), the temperature of the distribution `temper`, and the number of iterations required before we propose an exchange of points `times`.

```
# Define function to run parallel tempering
metropolis_hastings <- function(theta, model_outputs, sigma_sq_catch, sigma_sq_biomass,
                                l_like, sigma_spec, sigma_other, temper, times = 5) {
  for (time in 1:times) {
    ################################################################################
    # Start by updating other
    ################################################################################
    # Propose a new theta from a normal distribution
    new_theta <- theta
    new_theta[43] <- as.numeric(theta[43]+sigma_other*rnorm(n = 1, mean = 0, sd = 1))

    # Run the model with the new theta
    model_outputs_new <- run_LeMaRns_MCMC(NS_params, new_theta, Fs_array)

    # Calculate the likelihood
    l_like_new <- l_likelihood(model_outputs_new, fisheries_landings, survey_biomass,
                               sigma_sq_catch, sigma_sq_biomass)

    # Calculate the Hasting's ratio (based on temper)
    l_alpha <- sum(l_prior(new_theta))+l_like_new*temper-
      sum(l_prior(theta))-l_like*temper

    # Decide whether to accept the new theta
    if (exp(l_alpha) > runif(1)) {
      # If accepted, save the model outputs, the new theta and the likelihood
      model_outputs <- model_outputs_new
      theta <- new_theta
      l_like <- l_like_new
    }

    ################################################################################
    # Loop through the species and update b and F0 at the same time
    ################################################################################
    for (sp in 1:21) {
      # Propose new theta from a normal distribution
      new_theta <- theta
      new_theta[c(sp, 21+sp)] <- as.numeric(new_theta[c(sp, 21+sp)]+sigma_spec[sp,]*
                                              rnorm(n = 2, mean = 0, sd = 1))
```

```
      if (sum(l_prior(new_theta)) != -Inf){
        # Run the model with the new theta
        model_outputs_new <- run_LeMaRns_MCMC(NS_params, new_theta, Fs_array)

        # Calculate the likelihood
        l_like_new <- l_likelihood(model_outputs_new, fisheries_landings, survey_biomass,
                                   sigma_sq_catch, sigma_sq_biomass)

        # Calculate the Hasting's ratio (based on temper)
        l_alpha <- sum(l_prior(new_theta))+l_like_new*temper-
          sum(l_prior(theta))-l_like*temper

        # Decide whether to accept new theta
        if (exp(l_alpha) > runif(1)) {
          # If accepted, save the model outputs, the new theta and the likelihood
          model_outputs <- model_outputs_new
          theta <- new_theta
          l_like <- l_like_new
        }
      }
    }
  }
  # Return theta, model_outputs and l_like
  return(list(theta = theta, model_outputs = model_outputs, l_like = l_like))
}
```

For each value of `temper`, we propose a new `theta` (referred to as `new_theta`) that includes only a change in `other`. The size of the move is determined by `sigma_other` multiplied by a randomly selected number that is sampled from a normal distribution with a mean of zero and a standard deviation of one. The model is then run with `new_theta` and the likelihood and Hasting's ratio is calculated based on `temper`. The algorithm chooses to accept `new_theta` if the Hasting's ratio exceeds a randomly selected number that is sampled from a uniform distribution with lower and upper limits of zero and one respectively. If `new_theta` is accepted, `theta` is replaced by `new_theta`. `model_outputs` and the log-likelihood `l_like` are also updated.

In the next step, a new `theta` is proposed with a change in the `b` and `F0` associated with a given species. Similar to the above, the size of the move for each parameter is determined by `sigma_spec` multipled by a randomly selected number that is sampled from a normal distribution with a mean of zero and a standard deviation of one. Assuming the prior on `F0` is not zero (which may happen if the parameter value moves outside the bounds we specified in the prior function), the model is run with `new_theta` and the likelihood and Hasting's ratio is calculated. `new_theta` is accepted or rejected following the same rule as above. This process is repeated for each species in the model.

This process continues for a number of iterations (as defined by `times`) and the function returns `theta`, `model_outputs`, and `l_like`.

Running the algorithm

In order to run the MCMC algorithm, we therefore need to define the temperatures to be used in the parallel tempering, adjust `sigma_spec` and `sigma_other` based on these temperatures (as greater temperatures allow us to move further within the parameter space), and set up a new cluster as shown below:

```
# Define the temperatures to be used
tmps <- c(1, 0.75, 0.5, 0.25, 0.001)
```

```r
# Repeat theta, l_like, model_outputs, sigma_spec etc. based on the number of
# temperatures that we're using
theta <- rep(list(theta), length(tmps))
l_like <- rep(list(l_like), length(tmps))
model_outputs <- rep(list(model_outputs), length(tmps))
sigma_spec <- rep(list(sigma_spec), length(tmps))
sigma_other <- rep(list(sigma_other), length(tmps))
sigma_sq_biomass <- rep(list(sigma_sq_biomass), length(tmps))
sigma_sq_catch <- rep(list(sigma_sq_catch), length(tmps))

# Adjust sigma_spec and sigma_other based on the different temperatures to
# be used
sigma_spec[[1]] <- sigma_spec[[1]]
sigma_spec[[2]] <- sigma_spec[[1]]*1.1
sigma_spec[[3]] <- sigma_spec[[1]]*1.25
sigma_spec[[4]] <- sigma_spec[[1]]*1.5
sigma_spec[[5]] <- sigma_spec[[1]]*5

sigma_other[[1]] <- sigma_other[[1]]*0.9
sigma_other[[2]] <- sigma_other[[1]]*1.1
sigma_other[[3]] <- sigma_other[[1]]*1.25
sigma_other[[4]] <- sigma_other[[1]]*1.5
sigma_other[[5]] <- sigma_other[[1]]*5

# Set up a new cluster
clus <- makeCluster(5)

# Export variables to the cluster
clusterExport(clus, list("l_likelihood", "Fs_array", "run_LeMaRns_MCMC", "NS_params",
                         "no_fish", "fisheries_landings", "survey_biomass", "l_prior"))

# Load the LeMaRns package on each core
clusterEvalQ(clus, library(LeMaRns))
```

The MCMC algorithm can then be run using the following `while` loop:

```r
# Initialise i
i <- 1

# Set the number of times to exchange points between chains
swaps <- 3

# Run the algorithm until i reaches iter
while (i < iter) {
  # Run the parallel tempering algorithm
  temp <- clusterMap(cl = clus, metropolis_hastings, theta = theta,
                     model_outputs =  model_outputs, l_like = l_like,
                     sigma_spec = sigma_spec, sigma_other = sigma_other,
                     temper = tmps, sigma_sq_catch = sigma_sq_catch,
                     sigma_sq_biomass = sigma_sq_biomass,
                     MoreArgs = list(times = 5))

  # Store theta, model_outputs and l_like from each chain
```

```r
for (j in 1:length(tmps)) {
  theta[[j]] <- temp[[j]]$theta
  model_outputs[[j]] <- temp[[j]]$model_outputs
  l_like[[j]] <- temp[[j]]$l_like
}

# Exchange points between chains based on the number of swaps defined previously
for (tt in 1:swaps) {
  # Choose the chains to exchange points
  j1 <- sample(x = 1:(length(tmps)-1), size = 1)
  j2 <- j1 + 1

  # Calculate the Hasting's ratio
  l_alpha <- (l_like[[j2]]-l_like[[j1]])*tmps[j1]+(l_like[[j1]]-l_like[[j2]])*tmps[j2]

  # Decide whether to accept the exchange
  if(exp(l_alpha) > runif(1)){
    # If accepted, save the model outputs, the new theta and the likelihood
    print("switch") # optional progress report
    theta[c(j1, j2)] <- theta[c(j2, j1)]
    l_like[c(j1, j2)] <- l_like[c(j2, j1)]
    model_outputs[c(j1, j2)] <- model_outputs[c(j2, j1)]
    sigma_sq_biomass[c(j1, j2)] <- sigma_sq_biomass[c(j2, j1)]
    sigma_sq_catch[c(j1, j2)] <- sigma_sq_catch[c(j2, j1)]
  }
}

# Implement the Gibbs step on the first chain (sigma conditional on theta)
for (tp in 1:length(tmps)) {
  sigma_sq_catch[[tp]] <-
    1/rgamma(n = 21, shape = 0.1+(colSums(!is.na(fisheries_landings))/2),
             rate = 0.1+rowSums((t(log(fisheries_landings[,-1])-
                                   log(model_outputs[[tp]][["catch"]][ , -1])))^2,
                                na.rm = T)/2)
  sigma_sq_biomass[[tp]] <-
    1/rgamma(n = 21, shape = 2+(nrow(survey_biomass)/2),
             rate = 2+rowSums((t(log(survey_biomass[, -1])-
                                 log(model_outputs[[tp]][["biomass"]][ , -1])))^2)/2)
}

# Update the likelihoods
l_like <- mapply(model_outputs = model_outputs, sigma_sq_catch = sigma_sq_catch,
                 sigma_sq_biomass = sigma_sq_biomass, FUN = l_likelihood,
                 MoreArgs = list(fisheries_landings = fisheries_landings,
                 survey_biomass = survey_biomass), SIMPLIFY = FALSE)

# Store the results
cat(theta[[1]], sigma_sq_catch[[1]], sigma_sq_biomass[[1]], l_like[[1]],
    file = "LeMaRns_PT1.txt", "\n", append = T)
cat(theta[[2]], l_like[[2]], file = "LeMaRns_PT2.txt", "\n", append = T)
cat(theta[[3]], l_like[[3]], file = "LeMaRns_PT3.txt", "\n", append = T)
cat(theta[[4]], l_like[[4]], file = "LeMaRns_PT4.txt", "\n", append = T)
cat(theta[[5]], l_like[[5]], file = "LeMaRns_PT5.txt","\n", append = T)
```

```r
  print(l_like[[1]]) # optional progress report
  i <- i+1
}

# Stop the cluster
stopCluster(clus)
```

Within the loop, we first implement the parallel tempering algorithm and store the `theta`, `model_outputs`, and `l_like` in several list objects. We then propose several exchanges of points between chains (defined by `swaps`) and re-calculate the Hasting's ratio based on each exchange. A given exchange is accepted if the Hasting's ratio exceeds a randomly selected number that is sampled from a uniform distribution with lower and upper limits of zero and one respectively. If the exchange is accepted, `theta`, `model_outputs` and `l_like` are again updated.

The final step in the algorithm is the Gibb's step, which updates the variance parameters `sigma_sq_catch` and `sigma_sq_biomass`. `l_like` is updated once more and the stationary distribution of the first chain, which is stored in `LeMaRns_PT1.txt`, is the posterior distribution that we are interested in. The `theta` and `l_like` of the other four chains are stored in similarly-labelled text files for diagnostic purposes. The iteration number `i` is increased by one and the algorithm continues until `i` reaches `iter`.

Results

We discard the first 100 iterations as burn-in:

```r
# Load the results of the algorithm
res <- read.table("LeMaRns_PT1.txt")

# Discard the first 100 iterations
post <- res[-(1:100), ]
```

We can now plot the marginal posterior distributions of each of the uncertain parameters:

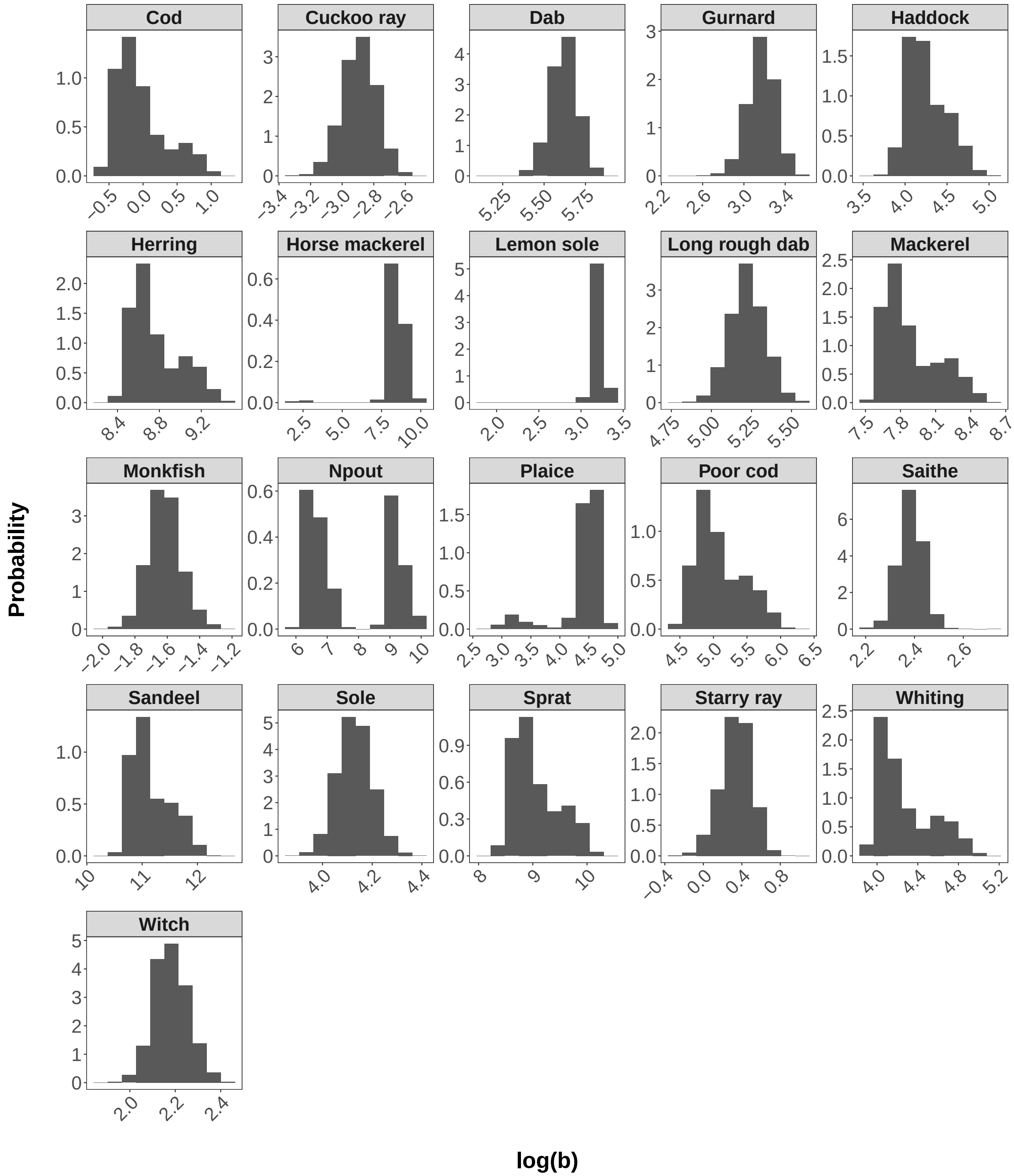
Cod
Cuckoo ray
Dab
Gurnard
Haddock
Herring
Horse mackerel
Lemon sole
Long rough dab
Mackerel
Monkfish
Npout
Plaice
Poor cod
Saithe
Sandeel
Sole
Sprat
Starry ray
Whiting
Witch
Probability
log(b)

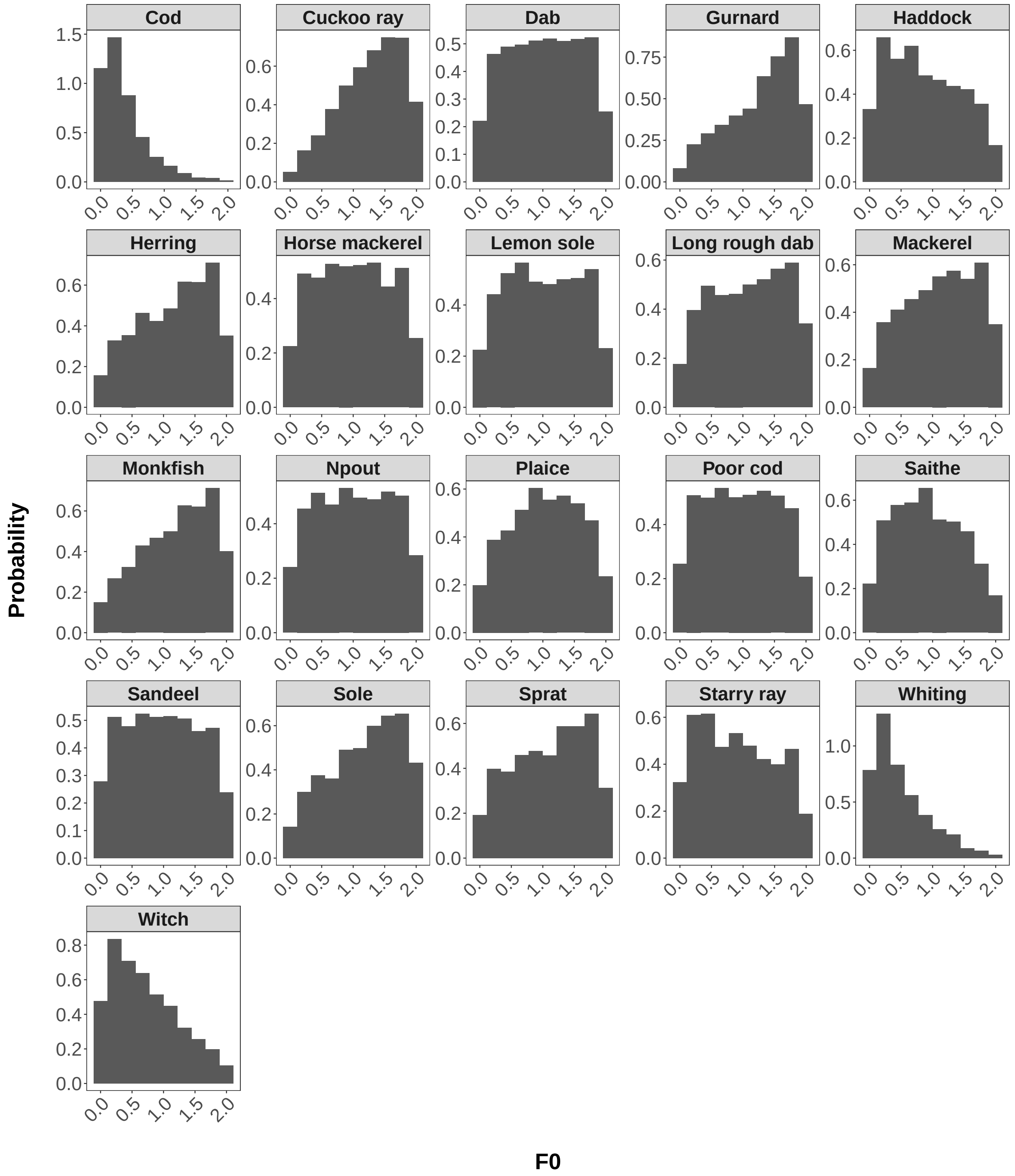

Cod
Cuckoo ray
Dab
Gurnard
Haddock
Herring
Horse mackerel
Lemon sole
Long rough dab
Mackerel
Monkfish
Npout
Plaice
Poor cod
Saithe
Sandeel
Sole
Sprat
Starry ray
Whiting
Witch
Probability
F0

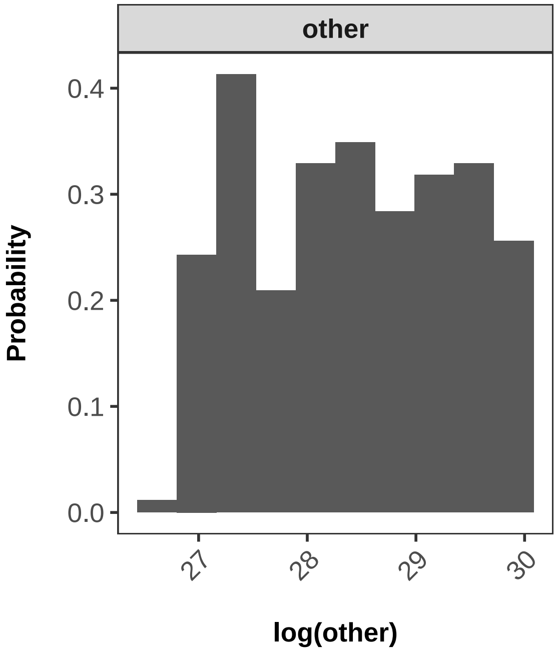

Finally, we can identify the parameter values associated with the highest likelihood:

```
# Identify the parameter values associated with the highest likelihood
b <- as.numeric(post[which.max(post[, ncol(post)]), 1:21])
exp(b)
```

```
##  [1] 8.038261e+03 7.322485e+02 8.249558e+04 1.756348e+02 1.848772e+02
##  [6] 2.599582e+02 7.507404e+03 5.379469e+03 2.518504e+01 6.446078e+01
## [11] 2.872764e+03 7.492533e+01 8.760097e+00 2.423226e+01 9.366251e+01
## [16] 1.774109e+00 6.599579e+01 4.997712e-02 2.105690e-01 7.656718e-01
## [21] 1.066107e+01
```

```
other <- as.numeric(post[which.max(post[, ncol(post)]), 43])
exp(other)
```

```
## [1] 1.205734e+12
```

All values of `b` are similar to the values identified during the history matching process. We might therefore surmise that if time constraints are an issue, the history matching process alone may be useful in improving model fit.

We can now run the model with the new parameter values, which results in the following outputs:

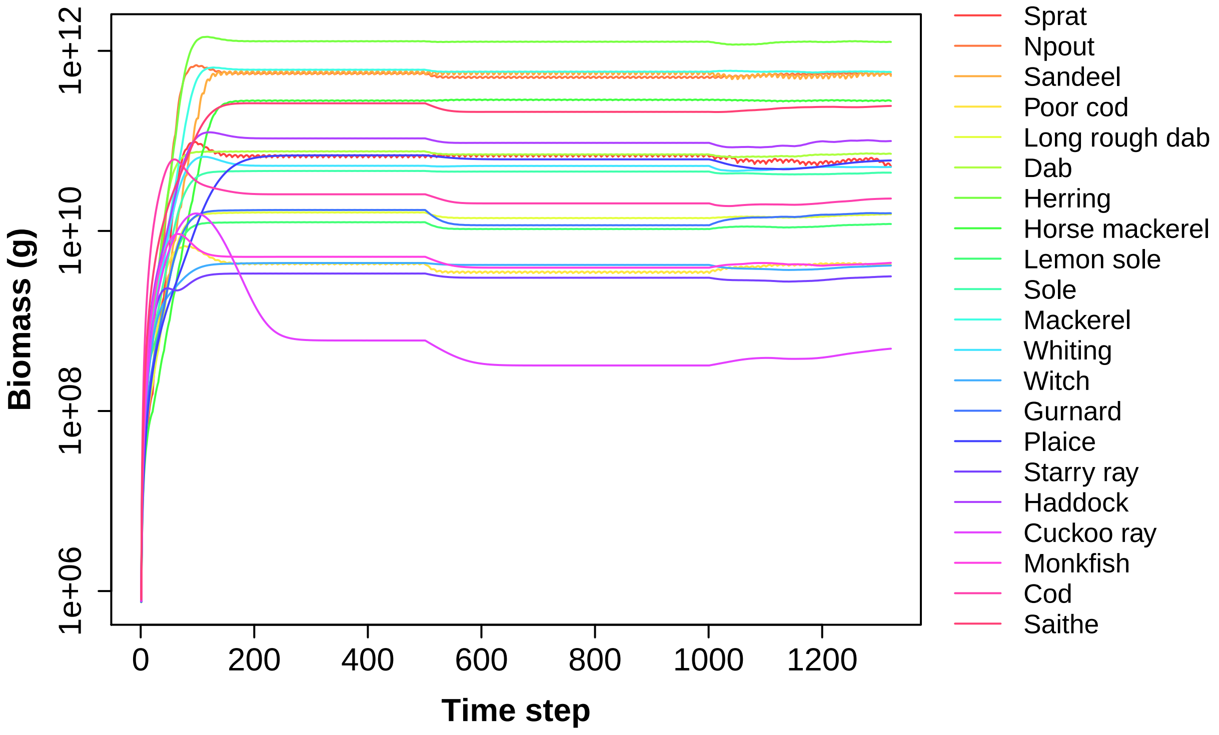
Biomass (g)
1e+06
1e+08
1e+10
1e+12
Time step
0
200
400
600
800
1000
1200
Sprat
Npout
Sandeel
Poor cod
Long rough dab
Dab
Herring
Horse mackerel
Lemon sole
Sole
Mackerel
Whiting
Witch
Gurnard
Plaice
Starry ray
Haddock
Cuckoo ray
Monkfish
Cod
Saithe

References

1. Plagányi ÉE. Models for an ecosystem approach to fisheries. FAO Fisheries Technical Paper 477, Rome, Italy; 2007.

2. Thorpe RB, Le Quesne WJ, Luxford F, Collie JS, Jennings S. Evaluation and management implications of uncertainty in a multispecies size-structured model of population and community responses to fishing. Methods in Ecology and Evolution. 2015;6: 49–58.

3. Rochet M-J, Collie JS, Jennings S, Hall SJ. Does selective fishing conserve community biodiversity? Predictions from a length-based multispecies model. Canadian Journal of Fisheries and Aquatic Sciences. 2011;68: 469–486.

4. Ward BA. Marine ecosystem model analysis using data assimilation. PhD thesis, University of Southampton.

5. Spence MA, Blackwell PG, Blanchard JL. Parameter uncertainty of a dynamic multispecies size spectrum model. Canadian Journal of Fisheries and Aquatic Sciences. 2015;73: 589–597.

6. Mackinson S, Platts M, Garcia C, Lynam C. Evaluating the fishery and ecological consequences of the proposed north sea multi-annual plan. PloS ONE. 2018;13: e0190015.

7. Gelman A, Carlin J, Stern H, Dunson D, Vehtari A, Rubin D. Bayesian data analysis. Third edition. Chapman and Hall; 2013.

8. Swendsen RH, Wang J-S. Replica monte carlo simulation of spin-glasses. Physical Review Letters. 1986;57: 2607.

9. Vernon I, Goldstein M, Bower R. Galaxy formation: Bayesian history matching for the observable universe. Statistical Science. 2014; 81–90.

10. ICES. Report of the working group on assessment of demersal stocks in the north se and skagerrak. 2017.

11. ICES. Database of trawl surveys (datras). 2017.

12. Walker ND, Maxwell DL, Le Quesne WJ, Jennings S. Estimating efficiency of survey and commercial trawl gears from comparisons of catch-ratios. ICES Journal of Marine Science. 2017;74: 1448–1457.

13. ICES. Official nominal catches. 2017.

14. Spence MA. Statistical issues in ecological simulation models. PhD thesis, University of Sheffield.
