# Supplementary material for: LeMaRns: A Length-based Multi-species analysis by numerical simulation in R
Source: PLoS One. 2020 Feb 3;15(2):e0227767. doi: 10.1371/journal.pone.0227767 (PMC6996808; doi:10.1371/journal.pone.0227767)
Supplement: Attachment — Submitted filename: Reviewer_resp.pdf [file pone.0227767.s004.pdf]

We thank both reviewers for their comments and feel that their input has greatly improved the paper. Our major changes are that we have rewritten the model overview section to better describe the LeMans model. We have also calibrated the model presented here and changed the data that comes with the package to the calibrated inputs. This has not changed the paper much but we have included the calibration as an additional appendix.

Below are out specific responses to the reviewers comments.

1 Reviewer 1

The manuscript describes the implementation of the LeMans model as package for R, LeMaRns. It includes several examples to demonstrate how the package can be used. Most of the details on the model and its implementation are in the accompanying vignette. The paper is generally well written and the example code seems to do what it is supposed to do. I think that the manuscript is generally suitable for publication but there are some relatively small issues that I would like to see first addressed.

I appreciate that the vignette provides most of the details about the model and how to use the package. However, some more model details in the main manuscript would be good so that readers didn't have to go digging too far to find out some of the basic model assumptions. For example, is the model single area? I assume so given the use of the predator-prey matrix for spatial overlap, i.e. a proxy for multiple areas.

We have re-written the 'Model overview' section in order to be more explicit about the LeMans model, pointing to the vignette for further details.

Additionally, the time step of the model is not clear from reading the manuscript. For example, the equation for fishing mortality is described using time step (line 95), but the arguments for the 'run_LeMans()' function includes year. Can the user determine the size of the time step? I see in the vignette that this is possible by using the 'phi_min' argument but a note about this could be added to the main text.

We have added the following sentence to the 'Model overview' section:

> A year in the model is subdivided into a number of equal time steps of length δt.

In the 'Setting up the model' section we also state that:

> The `LeMansParam()` function takes parameters described in the previous section as well as optional arguments: ... and δt, the time step of the model in years (`phi_min`, the default is `0.1`).

Following this, there is some inconsistency in how the time axes are labelled. The axes for Figures 1 and 5 are in time steps (the scale implying 10 time steps per year), whereas the axis for Figure 4 is year.

We have changed the axes labels so that 'Time step' is used in all plots.

Line 105, the predator-prey matrix, NS-tau, is described as containing information regarding the diet of the species in the model. It would be good to know what kind of information this is and how users might go about getting this information for their own models.

We have included some information about how users might define τ. We state that:

> Users may also input a predator-prey interaction matrix, `tau`, which describes the diet information and spatial overlap of predators and prey. `tau` defaults to one for all predator-prey combinations, although we recommend that this is replaced with an ecosystem-specific matrix based on available diet information, spatial overlap and/or expert judgement.

The supplementary R script works as it is supposed to and recreates the figures in the manuscript (tested using R 3.6.1). I note that the model does run very fast (certainly quicker than the initial implementation of the mizer model used to).

Thank you.

1.1 Specific points

Line 6: "However, over longer timescales models that take account of multi-species interactions, such as predation and competition for resources are required for meaningful predictions."

This is a strong statement. I don't necessarily disagree with it but it would be good see to some additional exploration and justification of this point. For example, it is not always the case that multi-species models are

better than single-species models for "meaningful" predictions. Multi-species models generally have higher data requirements, make more assumptions and are more difficult to parameterise than single-species models. This can result in an increase in uncertainty in the model predictions. Depending on the type of advice that is required for management it may be better to use a simple model, whose limitations are well understood, than a more complex model. Additionally, multi-species interactions may only become important when fishing mortality is relatively low, i.e. when fishing pressure is high it is the dominant source of mortality, in which case single-species models may be adequate. There are many challenges when using multi-species models, including LeMans, for example the estimation of an initial population abundance (as noted in the manuscript, which states that the provided data for the North Sea ecosystem has not been calibrated). This is simpler with a single-species model as they can often be used for stock assessment as well as projections.

I understand that the focus of the paper is not about the pros and cons of single- vs multi-species models but I think the introduction could benefit from a small discussion about when using a multi-species model might be appropriate and the kind of management advice it is able to provide, including the robustness of that advice.

We have amended this sentence. It now reads:

> However, these models assume that inter-stock interactions (predation and competition for resources) are either fixed or vary only in a simple way with time, and so on longer timescales their use becomes increasingly problematic. Multi-species models that explicitly represent some or all of these interactions are generally more suited to making predictions as the timescale of interest increases (1).

(1) is a paper discussing the issue in question. We have included a sentence that points the reader to this:

> For a discussion regarding the relative merits of single and multi-species approaches see (1).

Line 8: "several multi-species models have been developed".

There are certainly more than several multi-species models. Do the authors mean several multi-species modelling approaches?

We have changed ‘several’ to ‘a large number’. It now reads:

> As a result, a large number of multi-species models have been developed. The models take various approaches:...

Line 97: “..catch at length..”

Do the authors mean “catchability at length”?

We do mean ‘catchability at length’ and we have edited the sentence to reflect this.

Line 108: “Note that due to the generalisations of the LeMans model in the LeMaRns R-package, the provided data is not calibrated to the North Sea ecosystem and is therefore used for demonstration purposes only.”

This is slightly concerning. Does this means that the implementation here is not the full LeMans model? What are these generalisations and what are the key differences between the LeMans model (as described and reviewed in references 8 - 13) and this implementation as LeMaRns? How do they impact the data in the package so that it is essentially uncalibrated?

It’s a shame that the data has not been calibrated. Some notes on how calibration could be performed would be good, rather than just pointers to references, i.e. what data is required to perform tuning?

We have removed this sentence as we were being overly cautious about the model in the package. The package can recreate the models in (2) and (3) exactly. However, the model in the package is not the same as the model in (4; 5; 6) and (7) (all of which share the same parameterisation), as there is an *ad hoc* function related to recruitment that is specific to the North Sea fish community in the previously-published version of the LeMans model. We decided that we wanted `LeMaRns`to be for more general use of the LeMans model, rather than specifically tailored to the North Sea LeMans model, and we therefore did not include the *ad hoc* function in the package. It is possible to add this function and recreate the model of (4; 5; 6) and (7) exactly (we did this during development of the package).

That said, the outputs of our model and that of the model in (4; 5; 6) and (7) are very similar. Furthermore, any future work on the North Sea LeMans model will be done without this *ad hoc* function.

We have now calibrated the recruitment parameter `b` in the `NS_data` object by fitting the model to landings and survey data. We have included a description of this in S3. In the manuscript we now state that:

> An illustrative example, based on (8), can be found in S3.

The model is now calibrated to the North Sea.

** Line 117: Is it necessary to include the *other* argument? It isn't described in the main text and the default value (according to the man page for LeMansParam()) is 1e12 anyway.*

We have created an object, `NS_other`, that was calibrated. We have included the sentence:

> We also include `NS_other` that has been calibrated to represent other food for the North Sea model.

** Figure 1: There should be units on the y-axis for SSB.*

We have changed the package to include a `units` argument for `plot_SSB`, `plot_biomass` and all of the indicator plots. The default is set to grams for weights and centimeters for length. This enables the user to display units on the plots. It does not re-calculate the model outputs based on the units provided as we do not want to restrict the units that can be used in the model.

** Figure 2: Why are the axes inverted (i.e. go from high to low)? Also, there should be units on these axes.*

We believe the plot may have appeared oddly in the proof as it was plotted on a landscape page, but the axes go from low to high. Figure 1 is displayed in portrait in this document for reference. The units are yr^{-1} and the plot has been updated to reflect this.

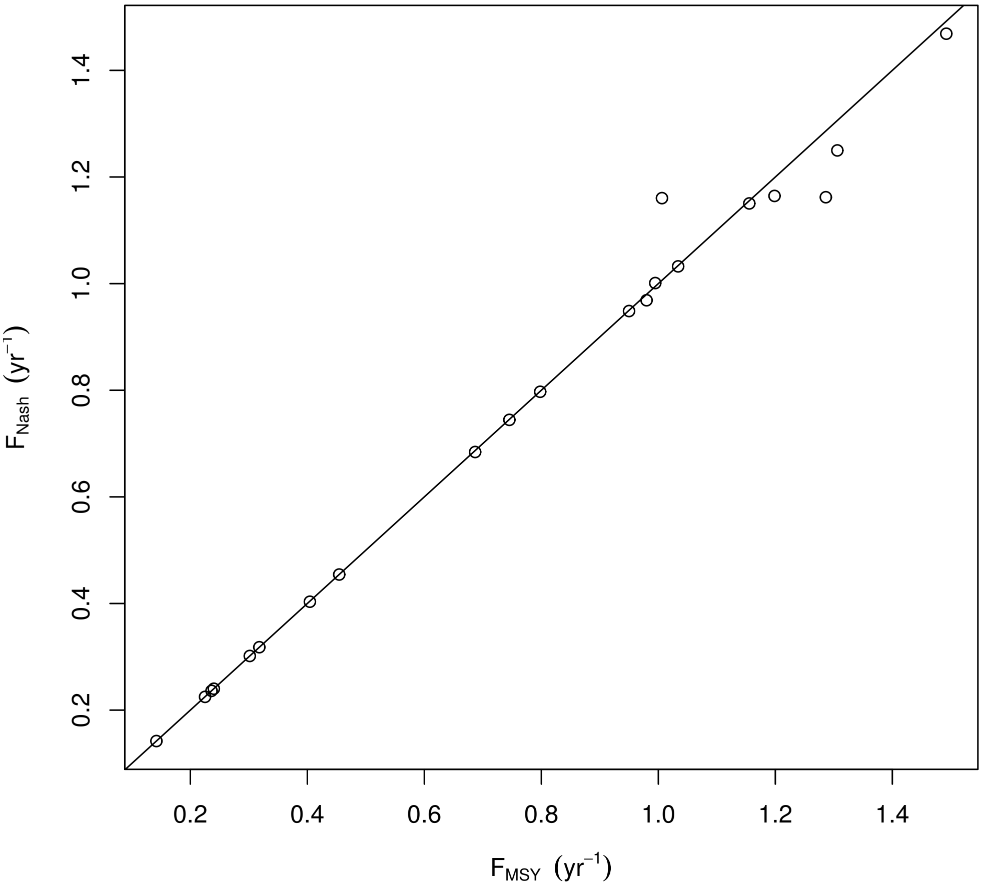

Figure 1: F_{MSY} **and** F_{Nash} **calculated using the LeMans model for the 21 species.** The solid line is the 1-1 line.

Line 186: "Using a factorial design..."

It is not immediately clear what this means. I can figure it out from the attached R script but this could be expanded in the text to add clarity.

We have changed this. It now reads:

> In a scenario, the effort of each of the four fishing fleets, `Industrial`, `Otter`, `Beam`, and `Pelagic`, was one of five levels, `c(0,0.5,1,1.5,2)`, which was held constant for 50 years. We ran all possible combinations of these levels resulting in 625 different scenarios.

There is a possible mistake in the package vignette. On page 13 of the vignette it says "Below we run the model for 10 years" but then the variable years is set to 50 (with a comment saying run for 10 years).

We have changed this to 50 years.

2 Reviewer 2

This manuscript introduces an R version of the ecosystem model LeMans first developed by Hall et al. 2006. The authors provide a very brief introduction to the model and a few examples of ways the model can be applied. The manuscript is well written but falls very short of any actual detail. The authors have decided to push almost all of the meat of this study into the supplemental materials. Although this may be what the journal wants, as a potential end-user of the package I find it very frustrating to not have the most pertinent information in the main text. As a result, the manuscript reads more like a vignette that could be included with the package on CRAN rather than the primary literature source for the model.

We have re-written the 'Model overview' section in order to be more explicit about the LeMans model. However, as the paper is only intended as an introduction to the package, we provide the full details of the model in the vignette in the supplementary material.

The statement on lines 108 - 110 concerns me quite a bit. It sounds like it is saying that the R package can not reproduce the LeMans outputs using the same data set? If so, that is not good. If a few more parameters need to be adjusted so that it will produce the same results than they should be included. The premise of the R package is that it is a easier more transparent version of LeMans but that may not be the case it it can't reproduce the results using the same data.

We have removed this sentence as we were being overly cautious about the model in the package. The package can recreate the models in (2) and (3) exactly. However, the model in the package is not the same as the model in (4; 5; 6) and (7) (all of which share the same parameterisation), as there is an *ad hoc* function related to recruitment that is specific to the North Sea fish community in the previously-published version of the LeMans model. We decided that we wanted `LeMaRns`to be for more general use of the LeMans model, rather than specifically tailored to the North Sea LeMans model, and

we therefore did not include the *ad hoc* function in the package. It is possible to add this function and recreate the model of (4; 5; 6) and (7) exactly (we did this during development of the package).

That said, the outputs of our model and that of the model in (4; 5; 6) and (7) are very similar. Furthermore, any future work on the North Sea LeMans model will be done without this *ad hoc* function.

We have now calibrated the recruitment parameter `b` in the `NS_data` object by fitting the model to landings and survey data. We have included a description of this in S3. In the manuscript we now state that:

> An illustrative example, based on (8), can be found in S3.

The model is now calibrated to the North Sea.

Another small editorial note, I wonder why the title of the package is LeMaRns, which stands for a Length-based Multi-species analysis by numerical simulation in R. Why not LeMansR? Not only is LeMansR easier to say it makes it obvious that it is an R version of LeMans and not a completely different model.

Thank you for the suggestion. Although we somewhat agree with this comment, the package is already available on CRAN and some people have already started using it. After a discussion with colleagues we have decided to keep the name `LeMaRns`. The package is pronounced Le-Marhh-ns.

2.1 Other detailed notes:

Line 40 - 41 - claim that LeMans is less complicated than Ecopath or Atlantis. It is true that it is less complicated than Atlantis but Ecopath does not require length data, stock-recruitment relationships, or catchability information.

We have removed Ecopath from this sentence.

Line 66- 69 - I don't think this is necessary to describe the model unless stepping through all of the code. A simple mention tht it is available of CRAN in the introduction should be sufficient.

We have re-written this to include GitHub as well. It now reads:

> `LeMaRns` is available on CRAN (`https://cran.r-project.org/web/packages/LeMaRns/index.html`) and GitHub (`https://github.`

`com/CefasRepRes/LeMaRns`).

Line 75 - Are you using the specialized or general k form of the von Bertalanffy growth equation (See Essington et al. 2001 CJFAS 58(11): 2129-2138)?

Thank you very much for this comment. It is the specialised von Bertalanffy growth function. We have changed the manuscript to read:

> ...the growth parameter from the specialised von Bertalanffy growth function (`k`) (9).

Line 85 - States that users may input a predator-prey matrix. Is there a default if they do not?

The default is 1 and we have included a sentence to make this explicit:

> `tau` defaults to one for all predator-prey combinations, although we recommend that this is replaced with an ecosystem-specific matrix based on available diet information, spatial overlap and/or expert judgement.

Line 96 - Effort and catchability need to be applied to a biomass or number which I assume is the Lj but the way it is written is not clear. Also, should the catchability term be specific to the length group so qkij instead of qik?

We have re-written the 'Model overview' section and in it we describe how F is applied to N. Specifically:

> The number of individuals after the mortality phase of the time step is

$$N''_{j,i,t} = N'_{j,i,t} \exp(-(M1_{j,i} + M2_{j,i,t} + F_{j,i,t})\delta t).$$

We have also adopted the reviewers notation suggestion.

Line 102 - A better sub-heading would be "Test Data Set"

We have renamed the sub-heading 'Test dataset'

Line 103 - While I'm sure the test data is in a data frame that is probably too technical and should just be referred to as the data set instead. It would also be helpful to flesh out why the test data set is included.

We have changed the text to read:

> In LeMans, we provide a dataset (`NS_par`) for 21 species in the North Sea based on (4).

The dataset is included for demonstrative purposes as well as for users to use when exploring or extending the package. We have calibrated the dataset so that it better represents the North Sea.

In addition in the mixed-fishery section we have changed *data frame* to "dataset" when talking about `NS_mixed_fish`.

Line 121 - What are the default values based on?

We have re-written this section to include the default values:

> The `LeMansParam()` function takes the parameters described in the previous section, as well as optional inputs including: n_l, the number of length classes (`nsc`, the default is `32`); the boundaries of the length classes (`bounds`, the default depends on `max(Linf)`) and δt, the time step of the model in years (`phi_min`, the default is `0.1`).

The default values are based on (4). This is stated at the end of the 'Setting up the model' section:

> All default values, with the exception of `tau`, are the same as those used in (4).

Lines 122 - 125 - Which is the default and why?

We have moved this text to the 'Biological data' section as we feel it belongs better there. We have included the default options. It now reads:

> In `LeMaRns` there are five built-in recruitment functions: `hockey-stick` (10) (the default option), `Ricker` (11), `Beverton-Holt` (9), `linear`, or `constant`(the default option is `hockey-stick)`,as well as three background mortality functions: `std_RNM` (the default option), `constant`, and `linear` (see S2, pages 5 and 7).

The defaults are the same as those used in (4). At the end of the 'Setting up the model' section we state that:

> All default values, with the exception of `tau`, are the same as those used in (4).

Line 139 - I'm guilty of this as well but it is bad form to use the same name for a variable as an argument (e.g. effort = effort).

We have changed this to:

```
no_of_gears <- dim(NS_params@Qs)[3]
effort_mat <- matrix(0.25, 50, no_of_gears)
model_run <- run_LeMans(NS_params, years=50, effort=effort_mat)
```

In addition we have changed the supplementary R-script and updated much of the vignette. However, we found this very difficult at the start of the vignette (S2) when we discuss how the package works. This is because when we run through the details of what the `LeMans_param` and `run_LeMans` functions do, we explicitly mean that the variable name and the argument are exactly the same thing.

Line 177 - 178 - Is this an expected result based on other Nash equilibrium studies?

Apart from (6), a study in the Baltic with three species (12), and a study with a theoretical size-spectrum model (13), we are unaware of any other studies that seek to find a Nash equilibrium, and in these they do not find single-species MSY. We have added a comment that states that single species MSY is sensitive to the fishing mortality of other species:

> In this study we arbitrarily chose to hold the fishing mortality of the other species at the F_{MSY} values given in (5). However, if we had chosen to hold them at $F_{Nash,-i}$, then $F_{MSY,i} = F_{Nash,i}$, $\forall i$, as $F_{Nash,i}$ is a solution of $F_{MSY,i}$. This highlights the importance of the fishing mortality on the other species when calculating $F_{MSY,i}$.

Line 213 - I'm not sure that this manuscript is as transparent as the authors intended. Other than calculations for Nash equilibrium there are no equations.

We have re-written the 'Model overview' section to provide a more explicit description of the model using equations. This section also points to details in the vignette, which provides all model formulations. Furthermore, the code is available on GitHub, which we now point out in the 'Using `LeMaRns`' section.

References

[1] Hollowed AB, Bax N, Beamish R, Collie J, Fogarty M, Livingston P, et al. Are multispecies models an improvement on single-species models for measuring fishing impacts on marine ecosystems? ICES Journal of Marine Science. 2000;57(3):707–719.

[2] Hall SJ, Collie JS, Duplisea DE, Jennings S, Bravington M, Link J. A length-based multispecies model for evaluating community responses to fishing. Canadian Journal of Fisheries and Aquatic Sciences. 2006;63(6):1344–1359.

[3] Rochet MJ, Collie JS, Jennings S, Hall SJ. Does selective fishing conserve community biodiversity? Predictions from a length-based multispecies model. Canadian Journal of Fisheries and Aquatic Sciences. 2011;68(3):469–486.

[4] Thorpe RB, Le Quesne WJF, Luxford F, Collie JS, Jennings S. Evaluation and management implications of uncertainty in a multispecies size-structured model of population and community responses to fishing. Methods in Ecology and Evolution. 2015;6(1):49–58.

[5] Thorpe RB, Dolder PJ, Reeves S, Robinson P, Jennings S. Assessing fishery and ecological consequences of alternate management options for multispecies fisheries. ICES Journal of Marine Science. 2016;73(6):1503–1512.

[6] Thorpe RB, Jennings S, Dolder PJ, editor: Shijie Zhou H. Risks and benefits of catching pretty good yield in multispecies mixed fisheries. ICES Journal of Marine Science. 2017;74(8):2097–2106.

[7] Thorpe RB, De Oliveira JAA. Comparing conceptual frameworks for a fish community MSY (FCMSY) using management strategy evaluation

- an example from the North Sea. ICES Journal of Marine Science. 2019;76(1):813–823.

[8] Spence MA, Blackwell PG, Blanchard JL. Parameter uncertainty of a dynamic multispecies size spectrum model. Canadian Journal of Fisheries and Aquatic Sciences. 2016;73(4):589–597.

[9] Beverton RJH, Holt SJ. On the Dynamics of Exploited Fish Populations. Springer Science + Business Media, Dordrecht; 1957.

[10] Ogle DH. Introductory Fisheries Analyses with R. CRC Press; 2016.

[11] Ricker WE. Stock and Recruitment. Journal of the Fisheries Research Board of Canada. 1954;11(5):559–623.

[12] Norrström N, Casini M, Holmgren N. Nash equilibrium can resolve conflicting maximum sustainable yields in multi-species fisheries management. ICES Journal of Marine Science. 2017;74(1):78–90.

[13] Farcas A, Rossberg AG. Maximum sustainable yield from interacting fish stocks in an uncertain world: two policy choices and underlying trade-offs. ICES Journal of Marine Science. 2016;73(10):2499–2508.
