# Supplementary material for: LeMaRns: A Length-based Multi-species analysis by numerical simulation in R
Source: PLoS One. 2020 Feb 3;15(2):e0227767. doi: 10.1371/journal.pone.0227767 (PMC6996808; doi:10.1371/journal.pone.0227767)
Supplement: Attachment — Submitted filename: reviewer_resp.pdf [file pone.0227767.s005.pdf]

We thank both reviewers for their comments and feel that their input has greatly improved the paper. We have addressed the comments of the second reviewer and changed the acknowledgements.

Below are out specific responses to the reviewers comments.

1 Reviewer 1

No comments

2 Reviewer 2

Abstract – final sentence says "...leading to increase uptake by fisheries management community". This is an optimistic statement that should say "we hope will lead to...". Which is how the authors say it in the main text (Line 36)

We have changed this to

> which we hope will lead to increased uptake by the fisheries management community

Line 63 – Can you provide a citation for how spatial information is included implicitly? We included a reference to the mizer package.

Line 78 – States that predation mortality is size- and species-dependent. I think there is a term missing from M2. Right now there is only a i (species), j (length), and t (time). Nothing for predator or predator size.

The equation below

$$N''_{j,i,t} = N'_{j,i,t} \exp(-(M1_{j,i} + M2_{j,i,t} + F_{j,i,t})\delta t),$$

is the number of individuals at time t of species i in length class j after mortality. $M2_{j,i,t}$ is the mortality caused by predation of the ith species in the jth length class. This is the sum of all predation for this species in this length class. The equations for this can be found on pages 4, 7-8 and 13 of S2. We felt that these equations are quite lengthy, are given in (1), and would dilute the main message of the paper so we decided to leave them out of the main paper. In the paper it says:

> Predation mortality is size- and species-dependent. The size preference of a predator is described using a preference function based upon a log-normal distribution, whilst species preference is described using a predator-prey interaction matrix indicating who eats whom (2; 3) (see S2 pages 4, 7-8 and 13 for more details).

Fishing mortality equation has a number but none of the other equations do.

This is the only equation that is numbered as it is the only equation that is referred to in the text. We follow the convention that only equations mentioned in the text are referenced. This has been done in PLOS ONE (e.g. 4).

Line 122 – delete "(the default option is hockey-stick)" as it is already stated earlier in the sentence.

Done. Thanks

Line 148 – It is unclear how NSother has been calibrated to represent other food. I understand that this was how the authors dealt with my concern over LeMaRns not recreating a published version but this needs further explaination.

The details of the calibration can be found in S3. We have re-written this paragraph to read:

> In `LeMaRns`, we provide a dataset, `NS_par`, for 21 species in the North Sea based on (3). The dataset contains `Linf`, `Lmat`, `W_a`, `W_b`, `k`, and the recruitment parameters (`a` and `b`) for each species. We also include `NS_other` to represent other food. The recruitment parameters, `a` and `b`, and `NS_other` were calibrated to the North Sea (see S3 for details). The predator-prey matrix (`NS_tau`) contains information regarding the diet of the 21 species and is based on (3). In addition, we provide information regarding a number of fishing fleets (`NS_mixed_fish`), with catchability parameters (`NS_eta` and `NS_L50`) that are based on (5).

Line 170 – The statement about the model is run for 10 years with no fishing can be confusing. Better to note that the default parameters are 10 years and 0 fishing effort.

We have re-worded this paragraph. It now reads:

> In LeMaRns a model can be run using the run_LeMans() function:
>
> ```
> run_LeMans(NS_params)
> ```
>
> By default, run_LeMans() uses the get_N0() function to initialise the population and is run for for 10 years with no fishing.

Line 179 – What is the units for effort? Is this relative effort?

The units of F are yr^{-1} and so the units of q are F per unit effort. In the examples provided effort is the maximum F.

We have added this to the manuscript.

> Fishing mortality is constructed from the joint effect of a number of fishing gears exploiting different species with different size and targeting preferences. More specifically, the fishing mortality of the ith species in the jth length class at time step t is
>
> $$F_{j,i,t} = \sum_{k=1}^{H} e_{k,t} q_{j,i,k}, \qquad (1)$$
>
> where $e_{k,t}$ is the effort of the kth gear, for $k = 1 \ldots H$ (the total number of gears in the fishery) and $q_{j,i,k}$ is the catchability. The units for F are yr^{-1} and the units of $q_{j,i,k}$ are F per unit effort.

In the section being referred to by the reviewer it now reads:

> Although gear catchability is calculated in LeMansParam(), fishing effort is an input to run_LeMans(), thus allowing effort to be dynamic. years is also an input to run_LeMans() and is used to define the number of years that the model should be run for. Below we run the model for 50 years with a constant effort of 0.25, which equates to an F of 0.25 on the length-class with highest selection, for each gear:
>
> ```
> no_of_gears <- dim(NS_params@Qs)[3]
> effort_mat <- matrix(0.25, 50, no_of_gears)
> model_run <- run_LeMans(NS_params, years=50, effort=effort_mat)
> ```

Conclusion – The authors should add a paragraph echoing the end of the abstract/ intro to tie everything together.

We thank the reviewer for this insight however after re-reading the end of the abstract and the introduction we are confident that the current 'Conclusions and further work' section appropriately acknowledges the packages many advantages. In order to re-iterate that the package has wide appeal, we have added a sentence acknowledging that the package could lead to an increased uptake by the fisheries management community. Moreover, we have acted to re-emphasise the open-source nature of the package by adding a note about the documentation. The first paragraph of the conclusion now reads:

> `LeMaRns` provides a convenient and user friendly way to run the LeMans model, with comprehensive documentation. The package contains the required functions to explore different fishing scenarios in a mixed-fisheries multi-species model and allows for the customisation and tailoring of inputs to model specific environments, conditions, and scenarios. This, along with the low data requirements, makes `LeMaRns` a transparent, easy to use, and broadly applicable fisheries assessment tool that encourages model development and experimentation. Further, we hope this will lead to an increased uptake of LeMans by the fisheries management community.

3 Additional changes

We have changed the acknowledgements. They now read

> This work was funded by the European Union's Horizon 2020 research and innovation programme under Grant Agreement No 773713 (PANDORA). We would also like to thank Gary Saggers, Finlay Scott, Sean M. Lucey and Athanassios C. Tsikiras for invaluable comments on an earlier version of the paper.

References

[1] Hall SJ, Collie JS, Duplisea DE, Jennings S, Bravington M, Link J. A length-based multispecies model for evaluating community re-

sponses to fishing. Canadian Journal of Fisheries and Aquatic Sciences. 2006;63(6):1344–1359.

[2] Rochet MJ, Collie JS, Jennings S, Hall SJ. Does selective fishing conserve community biodiversity? Predictions from a length-based multispecies model. Canadian Journal of Fisheries and Aquatic Sciences. 2011;68(3):469–486.

[3] Thorpe RB, Le Quesne WJF, Luxford F, Collie JS, Jennings S. Evaluation and management implications of uncertainty in a multispecies size-structured model of population and community responses to fishing. Methods in Ecology and Evolution. 2015;6(1):49–58.

[4] Cesnovar R, Strumbelj E. Bayesian Lasso and multinomial logistic regression on GPU. PLOS ONE. 2017;12(6):1–17. doi:10.1371/journal.pone.0180343.

[5] Thorpe RB, Dolder PJ, Reeves S, Robinson P, Jennings S. Assessing fishery and ecological consequences of alternate management options for multispecies fisheries. ICES Journal of Marine Science. 2016;73(6):1503–1512.
